# Recent highlights in the synthesis and biological significance of pyrazole derivatives

**DOI:** 10.1016/j.heliyon.2024.e38894

**Published:** 2024-10-09

**Authors:** Ziad Moussa, Mani Ramanathan, Shaikha Mohammad Alharmoozi, Shahad Ali Saeed Alkaabi, Salamah Hamdan Mohammed Al Aryani, Saleh A. Ahmed, Harbi Tomah Al-Masri

**Affiliations:** a Department of Chemistry, College of Science, United Arab Emirates University, P. O. Box 15551, Al Ain, United Arab Emirates; b Department of Chemistry, Faculty of Applied Sciences, Umm Al-Qura University, Makkah, 21955, Saudi Arabia; c Department of Chemistry, Faculty of Sciences, Al al-Bayt University, P. O. Box 130040, Mafraq, 25113, Jordan

## Abstract

Aza-heterocyclic scaffolds are privileged cores in the composition of their potential therapeutic profiles and versatile synthetic intermediates. Pyrazole is one of the frequently studied compounds of “azole” family and consists of nitrogen in a 1,2 linking sequence. These motifs possess a wide-spectrum of applications in the field of pharmaceuticals, agrochemicals, polymer chemistry, cosmetics, food industries and more. In addition, functionalized pyrazole derivatives are frequently used as ligands in coordination chemistry and metal-catalysed reactions. As exemplified by numerous recent reports, pyrazoles are highly promising pharmacophores with excellent therapeutic applications. Owing to their aromaticity, the ring structures have many reactive positions, where electrophilic, nucleophilic, alkylation and oxidative reactions might occur. The structural adroitness and diversity of pyrazole cores further emanated numerous fused bicyclic skeletons with various biological applications. In this review, we highlight the recent synthetic methods developed for the preparation of functionalized pyrazole derivatives (From 2017 to present). In addition, we have also covered the notable biological activities (anti-cancer, anti-inflammatory, anti-bacterial and anti-viral) of this ubiquitous core. Herein, we emphasised the synthesis of pyrazoles from variety of precursors such as, alkynes, α,β-unsaturated carbonyl compounds, diazo reagents, nitrile imines, diazonium salts, 1,3-dicarbonyl compounds and etc. Moreover, the recent synthetic methodologies focusing on the preparation of pyrazolines and pyrazolones and variously fused-pyrazoles are also included. Authors expect this review could significantly help the researchers in finding elegant novel tools to synthesize pyrazole skeletons and expand their biological evaluation.

## Graphical abstract

**Figure:**
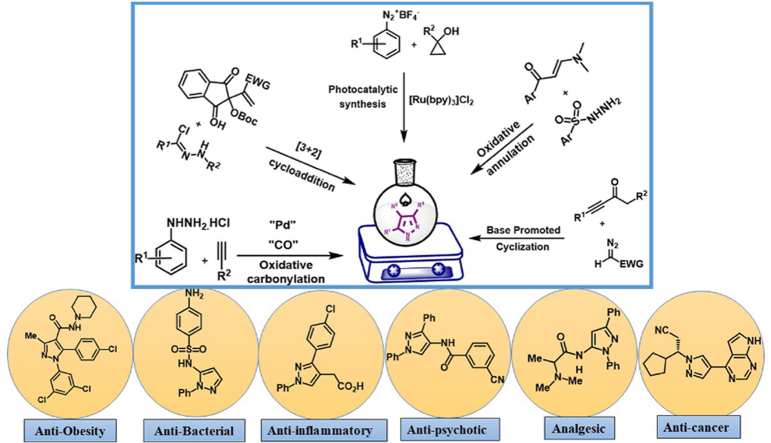

## 1 Introduction

*N*-containing heterocycles are one of the abundant molecules in nature and their synthetic and pharmaceutical efficacy have been well-documented in recent years [[1], [2], [3], [4], [5], [6]]. Subunit of these cores are frequently found in several biologically important natural products and alkaloids such as, serotonin [7], vitamin B1 [8], morphine [9], atropine [10], coniine [11], nicotine [12], caffeine [13] and various NSAIDs [14]. In addition, they are integral parts of various hormones, antibiotics, vitamins and nucleic acids (DNA and RNA) [[15], [16], [17]]. Over 60 % FDA approved drugs comprise *N*-heterocycles in their structural frameworks [18]. The frequent occurrence of *N*-heterocycles in biologically significant compounds could be due to their stability and bonding ability of nitrogen atoms [19]. Among these, Pyrazole derivatives are notable member of “azole” family, acquired special attention both in terms of synthetic design and of wide spectrum biological activities. As exemplified in Fig. 2a, the pharmacological importance of pyrazole could be revealed by its presence in diversified drugs such as, CDPPB (antipsychotic), Celecoxib (anti-inflammatory), Rimonabant (anti-obesity), Betazole (H2-receptor agonist), Difenamizole (analgesic) and Fezolamide (antidepressant) [[20], [21], [22], [23], [24], [25], [26]].

## 2 Aromatic nature and reactivity of Pyrazole core

**Fig. 1:**
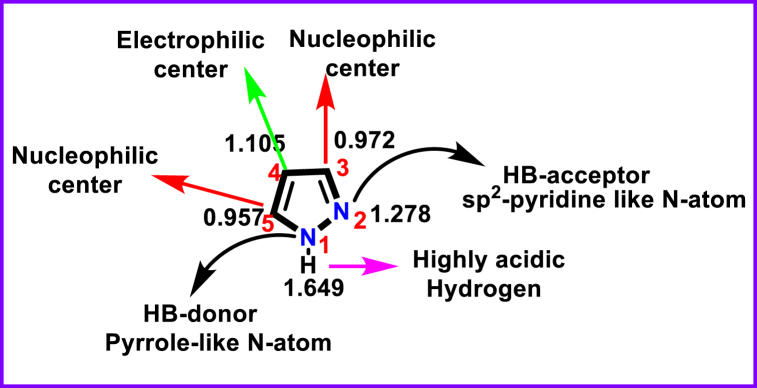
Structure of Pyrazole with π-electron density. Numbers in black refer to relative electron densities stating the electron rich nature of C4 and electron deficient nature of C3 & C5.

**Fig. 2:**
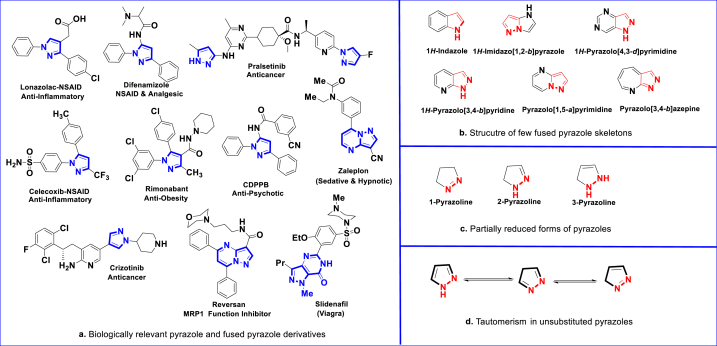
Representative examples of biologically important pyrazoles and fused pyrazoles. Fig. 2a. NSAID refers to non-steroidal anti-inflammatory drugs; CDPPB refer to 3-Cyano-*N*-(1,3-diphenyl-1*H*-pyrazol-5-yl)benzamide; MRP1 refer to multidrug resistance protein 1. Fig. 2b. Various structures of fused pyrazoles. Fig. 2c. Partially reduced forms of pyrazoles. Fig. 2d. Tautomeric structures of unsubstituted pyrazoles.

Within the azole family, pyrazoles are one of the extensively explored π-excess electrons firstly reported in 1883 by Ludwig Knorr [27]. It has a five-membered ring structure possessing two vicinal N-atoms, a pyridine type sp^2^-hybridized nitrogen, a pyrrole type acidic nitrogen and three carbons (Fig. 1). Unshared pairs of electrons on the -NH group and other π-electrons on the ring makes the pyrazole core a potentially aromatic system. Structurally, *N*-unsubstituted pyrazole derivatives act as both acids and bases (amphoteric) [28]. Acidic NH group readily donates the proton, whereas sp^2^-pyridine type *N*-atom easily accept protons. On the other hand, various substitutions on the ring structure can modify these properties. Various fused structures of pyrazole are well-studied and exhibited exceptional biological and material applications (Fig. 2b) [[29], [30], [31]]. Pyrazole also exist in partially reduced forms such as, 1-pyrazoline, 2-pyrazoline and 3-pyrazoline (Fig. 2c) [[32], [33], [34]]. Unsubstituted pyrazoles can exist in tautomeric structures (Fig. 2d).

The diversified chemical reactivity of pyrazole can be explained by the effect of atoms present in its core structure. H^+^ can be easily deprotonated from the N1 position in the presence of a base. sp^2^-nitrogen at N2 position is basic in nature and reacts with electrophilic reagents. C3 and C5 positions are suitable for electrophilic attack due to the decreased charge density caused by two nitrogen atoms [35]. On the other hand, in the presence of strong base, C3 position can be deprotonated and ring opening could occur. Similarly, pyrazolium ions can be formed by the addition of H^+^ ions.

## 3 Synthetic approaches for pyrazoles

**Fig. 3:**
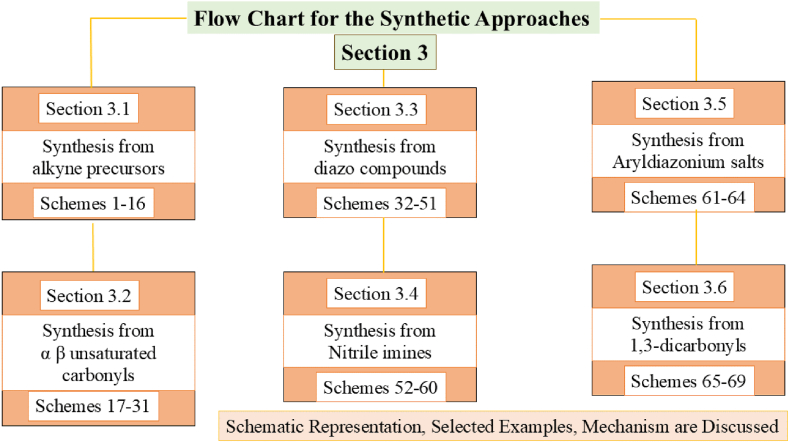
Flow chart of the synthetic approaches discussed under section 3.

Traditionally, pyrazole have been prepared by the reaction of (i) α,β-unsaturated carbonyls with hydrazine and further dehydrogenation [36], (ii) Condensation of 1,3-diketones with hydrazines [22], (iii) [3 + 2] cyclo addition involving 1,3-dipoles [37]. These methods suffer from high reaction temperature, longer times, low atom efficiencies, lower yields. To this end, in recent years variety of elegant methods are designed to prepare pyrazole derivatives from synthetically well-designed precursors under mild conditions. In this review, we highlight the synthesis of pyrazole derivatives from alkynes, α, β-unsaturated carbonyls, diazo compounds, *in situ* generated nitrile-imines, aryldiazonium salts and 1,3-dicarbonyl compounds. In addition, synthetic efforts for the preparation of partially reduced pyrazole derivatives (pyrazolines and pyrazolones) and fused pyrazoles are also included. Overall, key synthetic methods that are developed from 2017 onwards to prepare these pyrazole cores in an efficient and atom-economic manner are covered. An overview of the various synthetic strategies discussed in this review for the preparation of pyrazole derivatives is shown as a flow chart (Fig. 3).

### 3.1 Synthesis of pyrazoles from alkyne precursors

**Scheme 1:**
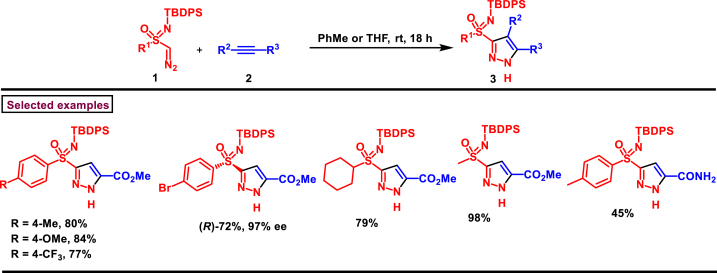
Synthesis of pyrazolesulfoximines *via* a [3 + 2] cycloaddition of alkynes and sulfoximine diazo compounds.

In 2024, Bull et al. reported a novel synthesis of pyrazolesulfoximines **(3)** *via* a [3 + 2] cycloaddition of alkynes **(2)** and sulfoximine diazo compounds **(1)** (Scheme 1) [38]. Novel sulfoximine tethered diazo substrates were prepared through sequential acetylation/diazo transfer/deacetylation. Electron withdrawing alkynes substituted with amides (1°, 2°, 3°) and weinreb amides were compatible under this reaction. Moreover, sulfone and ketone derived alkynes **(2)** were also found viable. Phenylacetylene was not reacted whereas, dimethylacetylne dicarboxylate overreacted to afford conjugate addition product. On the other hand, aryl, heteroaryl and alkyl sulfoximine diazo substrates **(1)** were studied and the corresponding products **(3)** were obtained in excellent yields. Biologically important skeletons such as, Fluoxetine and Nortriptyline were accessed under this process. A range of postsynthetic modifications suchas, *N*-benzylation, carboxy reduction, ester hydrolysis and desilyation were carried out to obtain synthetically valuable compounds.)

**Scheme 2:**
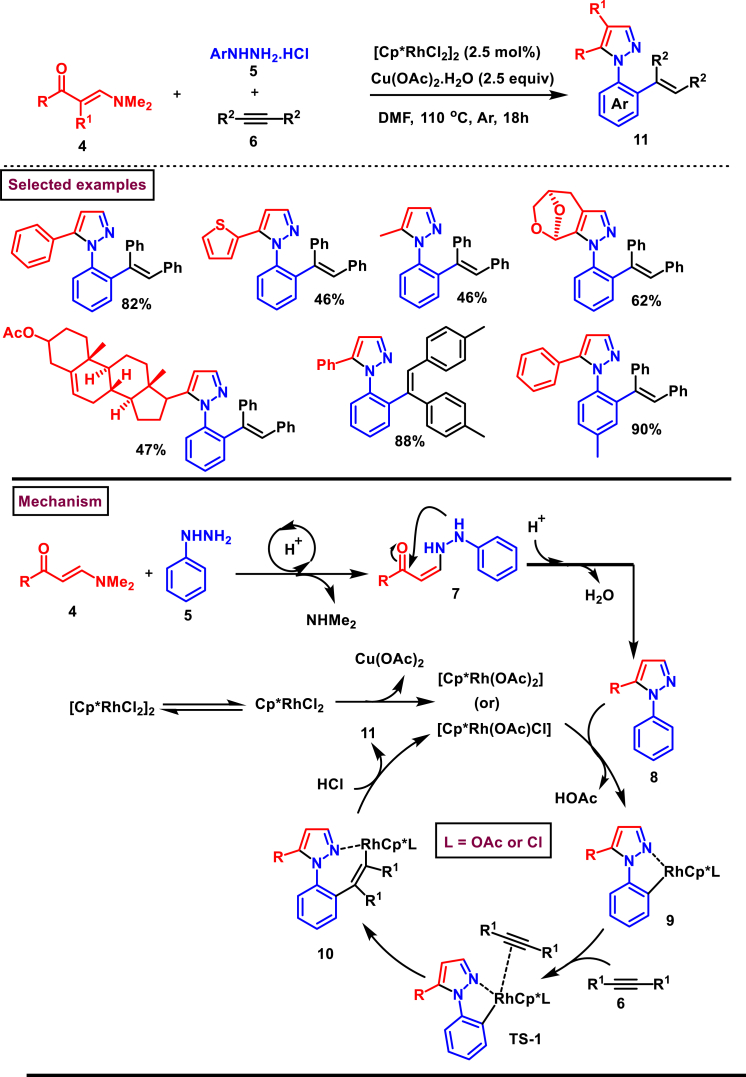
Rh-catalysed chemo-selective synthesis of *N*-(*o*-alkenylaryl)pyrazoles.

In 2023, Wan and Wen et al. have developed an efficient and chemo selective Rh-catalysed route to prepare *N*-(*o*-alkenylaryl)pyrazoles (**11**) from easily available enaminones (**4**), alkynes (**6**) and hydrazine hydrochlorides (**5**) (Scheme 2) [39]. This method tolerated a variety of functionalized enaminones **(4)**, arylhydrazines **(5)** and symmetrical alkynes **(6)** and provided the corresponding *N*-(*o*-alkenylaryl)pyrazoles (**11**) in moderate to excellent yields. When unsymmetrical internal alkynes were employed, isomeric products were observed in 1:1 ratio. Mechanistically acid promoted transamination of **(4)** and **(5)** resulted in the intermediate **(7)** which underwent intramolecular condensation to afford the pyrazole **(8)**. *ortho*-C-H bond insertion *via* active Rh-complex and alkyne insertion to the Rh-center led to complexes **(9)** and **(10)** *via* TS-1. Protonation of C-Rh bond with an acid (HCl) allowed the regeneration of Rh-catalyst and released the desired product **(11)**. It was also observed that, the pyrazole ring **(8)** was mandatory for the C-H alkenylation process as it make a N-coordinating complex with the Rh-catalyst. Moreover, based on the deuterium label studies, C-H activation step was claimed to be a rate determining step in the entire process.

**Scheme 3:**
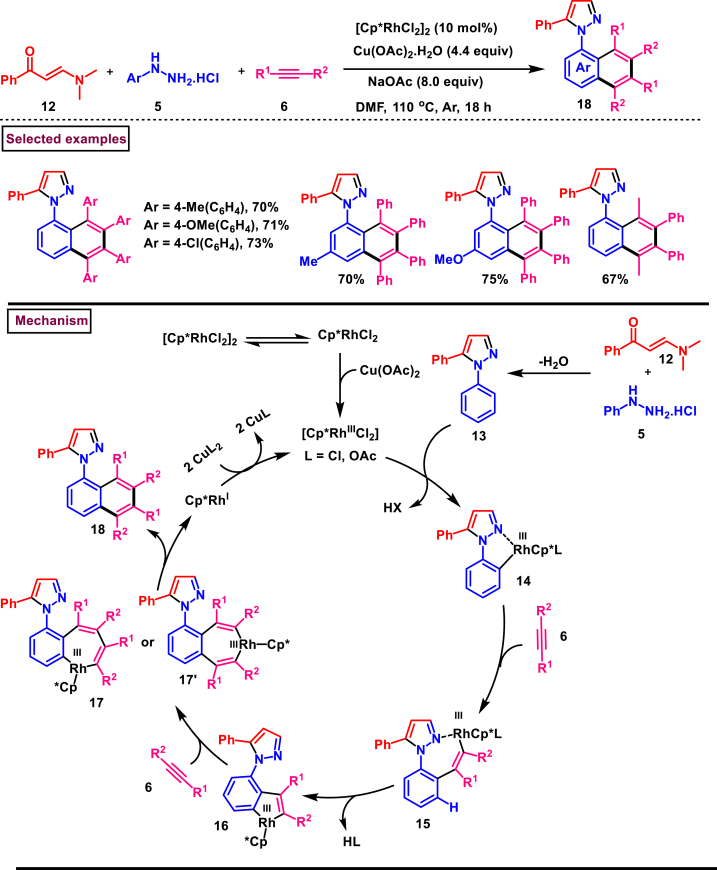
Multicomponent synthesis of pyrazoles *via* Rh(III)-catalysed benzannulation of enaminone, hydrazine and alkyne.

Rh-catalysed novel cascade approch to access pyrazole derivatives bearing naphthalene moiety **(18)** was reported by Wan and Liu et al. (Scheme 3) [40]. This method enabled the synthesis of pyrazoles *via* an one-pot pyrazole formation and sequential benzannulation from alkynes **(6)**, enaminones **(12)** and hydrazine hydrochlorides **(5)**. Variously functionalized aryl, alkyl and heteroaryl based enaminones **(12)** were conveninetly reacted to give the corresponding products **(18)** in moderate to good yields (51%–75 %). Strongly electron-withdrawing pyridinyl enaminone failed to react under the reaction conditions. Moderate scope was exerted with respect to hydrazine and alkynes. Symmeterical and unsymmetrical alkynes **(6)** were suitable and provided the desired product **(18)** in good yields. Intrestingly, enaminal was also found viable substrate and the product was obtained in 72 % yield. Based on the experimental observation, a plausible mechanism stated that, initial condensation of hydrazine chloride **(5)** and enaminone **(12)** provided the pyrazole **(13)**. Pyrazole assisted C-H activation with active Rh^III^-catalyst gave the complex **(14)**. Further coordination with alkyne **(6)** and C-H activation resulted in metallocycle **(15)**. Based on the C-Metal bond cleavage, another set of alkenyl metalation led to two possible intermediates **(17** and **17’)**. Reductive elimination of Rh^I^ yilded the product **(18)** and Cu(II) oxidation regenerated the Rh-catalyst.

**Scheme 4:**
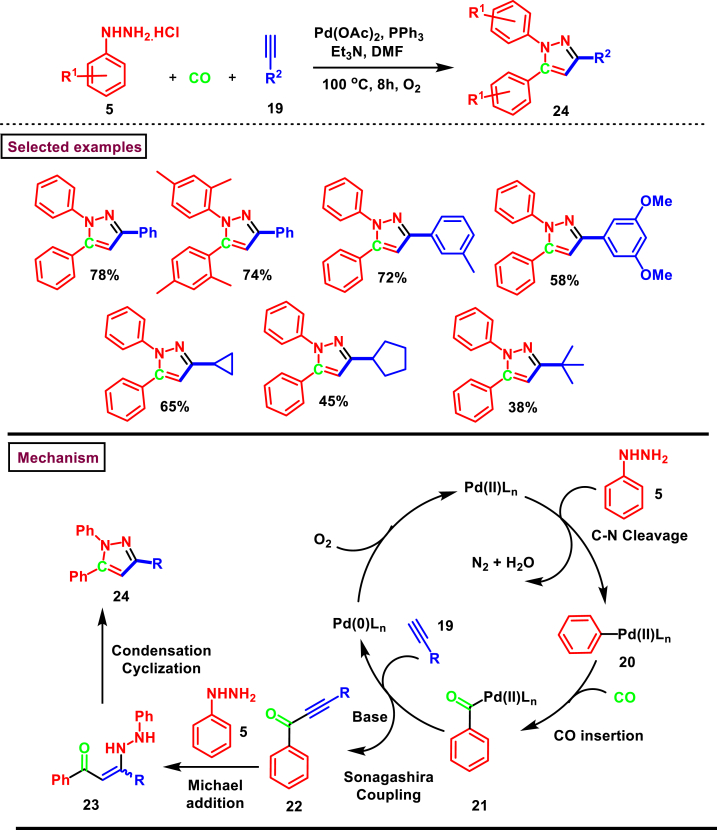
Pd-catalysed synthesis of trisubstituted pyrazoles *via* oxidative carbonylation.

A facile synthesis of trisubstituted pyrazoles (**24**) from arylhydrazines (**5**) and alkynes (**19**) *via* a Pd-catalysed oxidative carbonylation sequences with excellent regioselectivity was reported by Zhao and Wang et al. (Scheme 4) [41]. In this multicomponent strategy, arylhydrazine (**5**) served as an arylating agent and provided ynone **(22)** *via* oxidative Songashira-carbaonylation conditions which again could be trapped by another unit of arylhydrazine **(5)** to yield pyrazoles **(24)**. Mechanistically, five important steps were involved in this tandem procedure such as C-N bond cleavage, CO insertion **(21)**, Sonagashira coupling of acylpalladium complex **(22)** with alkyne **(19)**, Michael addition of hydrazine unit with ynone and cyclization. Varieties of mono and di-substituted arylhydrazines (**5**) were participated in the CO-insertion step except hydrazines with strong electron-withdrawing groups and aliphatic hydrazines. Similarly, both alkyl and aryl alkynes **(19)** afforded the pyrazoles (**24**) in moderate to good yields (see Scheme 2).

**Scheme 5:**
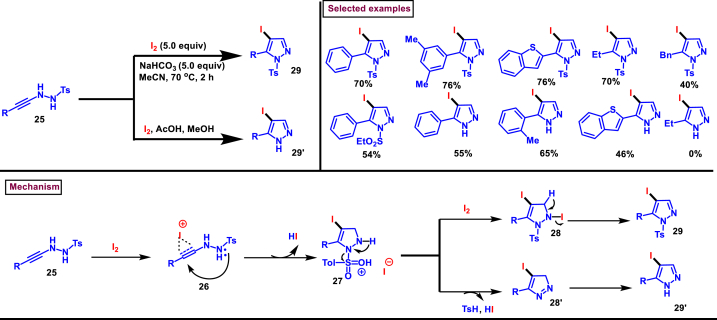
Preparation of 4-iodo pyrazoles from 5-*endo*-dig-iodocyclization of *N*-propargyl-*N*′-tosylhydrazines.

In 2022, Togo et al. disclosed the synthesis of 5-functionalized-4-iodo-1-tosylpyrazoles (**29**) from the reaction of *N*-propargyl-*N*′-tosylhydrazines (**25**) and I_2_ in the presence of a base (Scheme 5) [42]. This transition metal-free iodocyclization proceeded efficiently to give the desired products in good yields. Tosyl functionality could be easily removed and the 5-aryl-4-iodopyrazoles (**29′**) were generated by performing the reaction in presence of acetic acid. Effect of temperature, base, solvent and the durations were studied in detail to find the optimal reaction conditions. *N*-arylpropargyl-*N*′-tosylhydrazines (**25**) with differently substituted aryl rings were participated and the products were obtained in moderate to good yields. Important functional groups transformations such as, de-iodination, Pd-catalysed alkynyl and aryl couplings were performed to showcase the synthetic potential of the pyrazole derivatives. Mechanistically, I_2_ activated the alkyne unit of *N*-propargyl-*N*′-tosylhydrazine (**25**) which underwent 5-*endo-dig* cyclization **(26)** followed by HI elimination in presence of base and furnished 5-functionalized-4-iodo-1-tosylpyrazoles (**29**). On the other hand, when intermediate **(16)** was activated by HI/AcOH, 5-aryl-4-iodopyrazoles **(29′)** were formed *via* elimination of TsOH and isomerization steps (see Scheme 4).

**Scheme 6:**
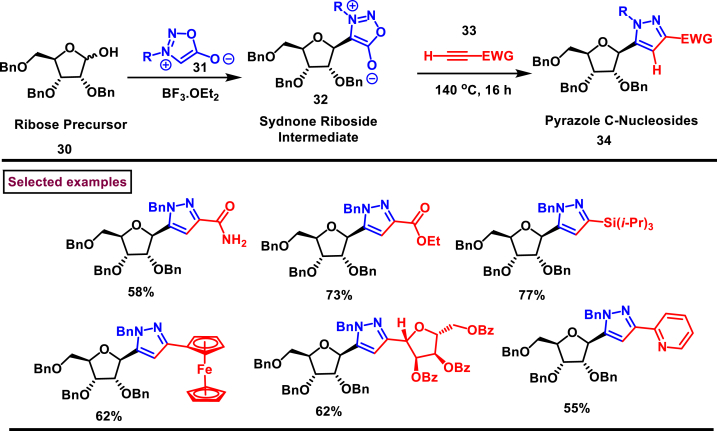
Synthesis of *N*-protected-5-functionalized pyrazoles *via* a [3 + 2] cycloaddition.

Calenbergh et al. reported a practical method to prepare naturally occurring C-nucleosides (**34**) such as formycin B and pyrazofurin *via* the intermediacy of syndone riboside (**32**) (Scheme 6) [43]. This versatile common intermediate was obtained by BF_3_.Et_2_O catalysed dehydrative electrophilic glycosylation which was operationally simple and high yielding compared to nucleophilic C-glycosylation. A series of *N*-protected-5-functionalized pyrazoles (**34**) were accessed *via* a [3 + 2] cycloaddition of alkyne (**33**) and syndone intermediate (**32**) under thermal conditions. Authors further observed the predominant formation of 1,3,5-substituted pyrazoles when employing terminal alkynes. These pyrazole end products were converted into formycin B and pyrazofurin *via* simple steps.

**Scheme 7:**
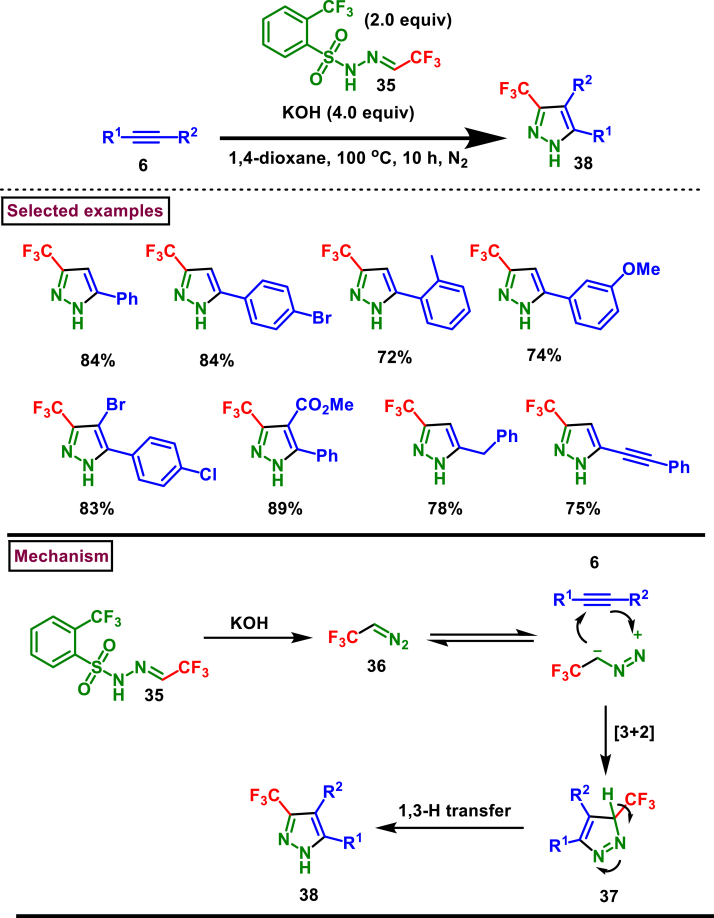
Metal-free synthesis of 3-CF_3_ pyrazoles *via* [3 + 2] cycloaddition of alkynes.

A transition metal-free approach for the synthesis of diverse 3-CF_3_ pyrazoles (**38**) was reported by Bi et al. from the [3 + 2] cycloaddition of alkynes (**6**) and trifluoroacetaldehyde *N*-triftosylhydrazone (**35**) (Scheme 7) [44]. This method provided an operationally safe procedure to access 3-CF_3_ pyrazoles **(38)** using non-toxic TFHZ-Tfs **(35)** which released CF_3_CHN_2_ **(36)** *in situ*. Suitability of this method was investigated with aryl, alkyl terminal alkynes, heteroaryl, internal alkynes and the corresponding products (**28**) were isolated in good to excellent yields. Notably, conjugated diyne and enyne underwent the reaction only at the terminal alkyne part and afforded the desired products regioselectively. A key pyrazole intermediate in the synthesis of antiarthritic drug Celecoxib was also prepared in a gram-scale synthesis (30 mmol). Reaction pathway was initiated by the *in situ* formation of CF_3_CHN_2_ **(36)** and its 1,3-dipolar cyclization with alkynes **(6)**, followed by a 1,3-hydrogen transfer **(37)** delivered the desired pyrazoles (**38**).

**Scheme 8:**
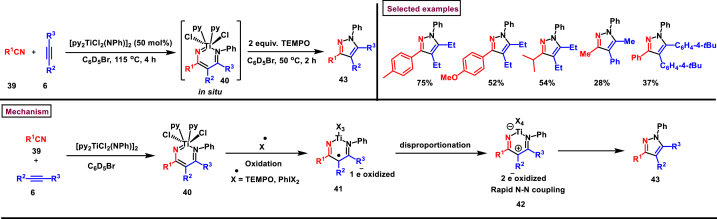
One-pot synthesis of functionalized pyrazoles *via* novel [2 + 2+1] cycloaddition.

In 2020, Tonks and co-researchers were realized a one-pot, multicomponent method to synthesize highly functionalized pyrazole derivatives (**43**) from the oxidative coupling of nitriles (**39**), alkynes (**6**), and Ti-imido complexes (Scheme 8) [45]. This novel [2 + 2+1] cycloaddition proceeded through the oxidation induced N-N reductive elimination from diazatitanacyclohexadienes intermediate **(40)** which in turn can be obtained by the coupling of Ti-imidos, alkynes **(6)** and nitriles **(39)** or by ring opening of 2-imino-2*H*-azirines or by metalation of 4-azadiene-1-amines. Mechanistic analysis suggests that N-N coupling at Ti-metal centre was initiated by 2-electron oxidation of key intermediate diazatitanacyclohexadiene (**42**) *via* electrocyclic processes. Broad range of nitriles **(39)** with electron-rich and electron-withdrawing groups tolerated and the desired pyrazoles (**43**) were obtained in good yields. Similarly, both symmetrical and unsymmetrically substituted alkynes **(6)** were also participated to furnish the desired products in moderate yields. Notably, N-N bond in the pyrazoles was installed by the oxidation induced coupling of Ti, thus avoids the need for hazardous reagents like hydrazine.

**Scheme 9:**
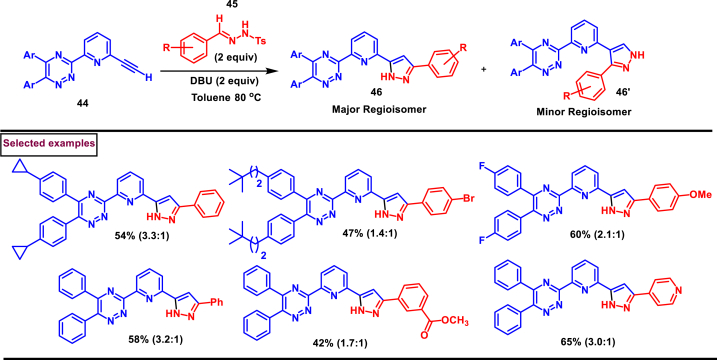
DBU mediated synthesis of pyrazoles *via* [3 + 2] dipolar cycloaddition.

A previously unexplored sets of pyrazoles with pyridine-2-yl- [1,2,4]-triazine was reported by Carrick et al. (Scheme 9) [46]. This intermolecular, DBU-mediated [3 + 2] dipolar cycloaddition of terminal ethyne unit of 3-(6-ethynyl-pyridine-2-yl)-5,6-diphenyl- [1,2,4]triazine (**44**) and the arylhydrazide (**45**) afforded 1*H*-Pyrazol-5-yl-pyridin-2-yl- [1,2,4]triazines (**46** and **46’**) under metal and oxidant free conditions. Moreover, excellent substrate scope with respect to electronically and sterically divergent dipolarophile and dipole was exerted. Ten-fold scale up reactions, post-synthetic derivatization, structural elucidation of major regio-isomer (**46**) by XRD, DFT calculations to support the experimental observations are the notable features of this unique report.

**Scheme 10:**
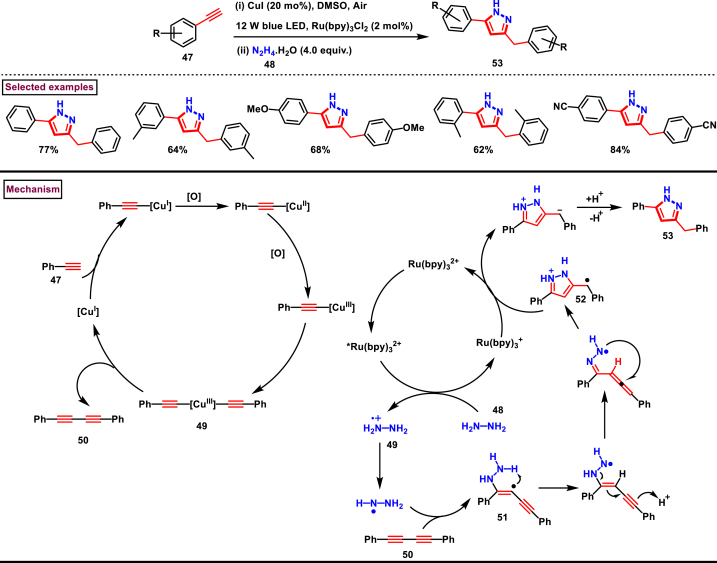
Synthesis of pyrazoles *via* Cu-catalysed Glaser coupling/annulation cascade.

In 2019, Zhu et al. reported an elegant Cu-catalysed Glaser coupling/annulation method to prepare multi-substituted pyrazoles (**53**) by employing alkynes **(47)** and hydrazines **(48)** under visible-light mediated cascade conditions (Scheme 10) [47]. Broad range of phenyl acetylenes (**47**) and hydrazine derivatives were readily involved in the reaction and the desired products (**53**) were isolated in good yields. Mild reaction conditions, environmentally benign conditions (Green oxidant O_2_) are the notable features of this report. Detailed control experiments and optimization studies were carried out to support the reaction mechanism. Under photochemical conditions phenyl acetylene **(47)** was converted into diyne **(50)** *via* several Cu-acetylide complexes. Hydrazine **(48)** was further quenched the excited Ru^II^∗ and converted into radical cation **(49)** which subsequently reacted with diyne **(50)** to give the radical intermediate **(51)**. Sequential intramolecular H-abstraction and tautomerization and cyclization afforded the cyclized radical intermediate **(52)** which further quenched the Ru^I^ oxidatively and converted into desired product **(53)** .

**Scheme 11:**
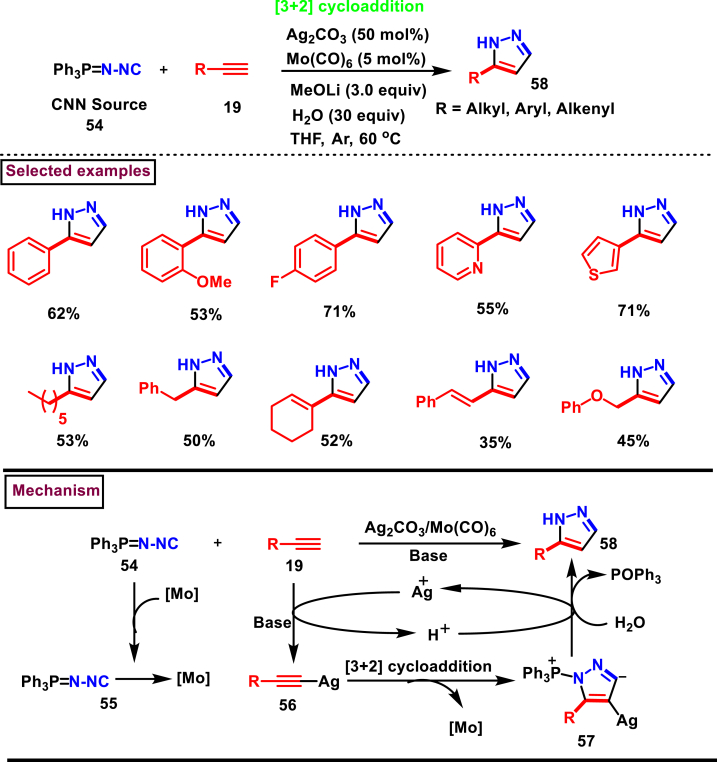
Ag/Mo mediated [3 + 2] cycloaddition of functionalized isocyanides and alkynes.

In 2019, Bi et al. have developed a silver-mediated novel method to access mono-substituted pyrazole derivatives (**58**) *via* a [3 + 2] cycloaddition of *N*-Isocyaniminotriphenylphosphorane, (**54**, a functionalized isocyanide) and an alkyne (**19**) [48]. Under the optimal reaction conditions, scope of this method was studied with varieties of electronically and sterically distinct alkynes (Scheme 11). In all these cases excellent reactivity was exerted and the desired products (**58**) were obtained in good yields. Based on the control experiments and optimization studies, authors have declared that the presence of silver catalyst and Mo(CO)_6_ were necessary to activate the alkyne and isocyanide precursors. A plausible mechanism for the preparation of synthetically useful pyrazole derivatives *via* [3 + 2] cycloaddition sequence was proposed. Accordingly, Silver acetylide **(56)** and molybdenum isocyanide complex **(55)** were generated from **(19)** and **(54)**. [3 + 2] cycloaddition between these intermediates resulted in the formation of cyclized intermediate **(57)**. Facile hydrolysis of **(57)** gave the desired product **(58)**.

**Scheme 12:**
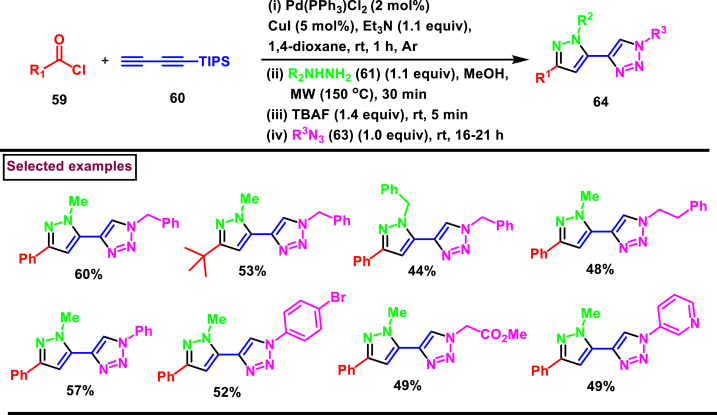
Synthesis of 4-pyrazolyl-1,2,3-triazoles *via* Pd-Cu catalysed CuAAC process.

In 2018, Muller's team reported a four-component synthesis for the preparation of 4-pyrazolyl-1,2,3-triazoles (**64**) *via* a Pd-Cu catalysed one-pot alkynylation-cyclocondensation-desilylation-CuAAC process (Scheme 12) [49]. Intrestingly, triisopropylsilyl butadiyne (**60**) was used as a four-carbon precursor in this process. TIPS-protected pentadiynones were prepared under Sonagashira conditions followed by a cyclocondensation with hydrazine **(61)**, TBAF mediated desilylation and CuAAC with azides **(63)** furnished the title compounds **(64)** without the requirements of addtional Pd or Cu catalysts. Broad range substrates with various functionalities were participated in this one-pot four step sequences except aryl hydrazines due to their lower nucleophilicity. Overall, this method exhibited high bond-forming efficiency and reagents were utilized in stochiometric quantities making this method practical.

**Scheme 13:**
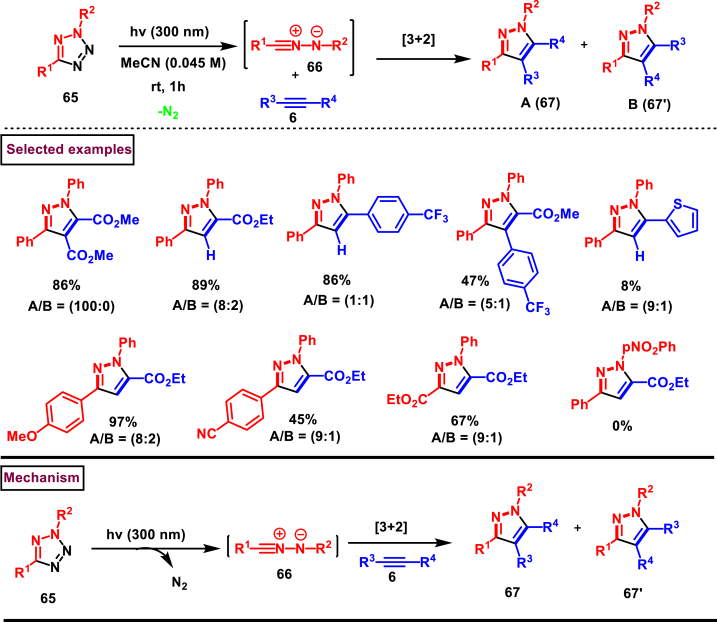
Synthesis of pyrazoles *via* photo-click reaction/1,3-dipolar cycloaddition sequences.

A photo click reaction between 2,5-disubstituted tetrazole derivatives (**65**) and alkynes (**6**) were carried out to yield pyrazole derivatives (**67** and **67′**) by Bochet et al. (Scheme 13) [50]. Extrusion of nitrogen from 2,5-disubstituted tetrazole (**65**) under photochemical conditions resulted in the *in situ* generation of nitrilimines **(66)** (dipoles) which reacted with alkynes (dipolarophile) *via* 1,3-dipolar cycloaddition. Access to broad range of pyrazoles with versatile functionalities (**67** and **67′**) have been showcased under an atom-economic and environmentally benign conditions. A systematic study on substrate scope was done with a range of tetrazoles and alkyne precursors. Photochemically generated nitrilimines **(66)** were trapped in presence of excess concentrated HCl and isolated as hydrazonoyl chloride. Mechanistically, authors have demonstrated that the photochemical N_2_ extrusion occurs either through intramolecular concerted radical recombination followed by a tautomerization of nitrilimine or through a heterolytic cleavage of tetrazole **(65)** and led to the formation of reactive intermediate **(66)**. [3 + 2] cycloaddition between alkyne precursor and **(66)** gave the desired products (**67** and **67’**) as a regioisomeric mixtures. Detailed study on the electronic effects, solvent effects, regioselectivity and gram scale synthesis are the other notable features of this report.

**Scheme 14:**
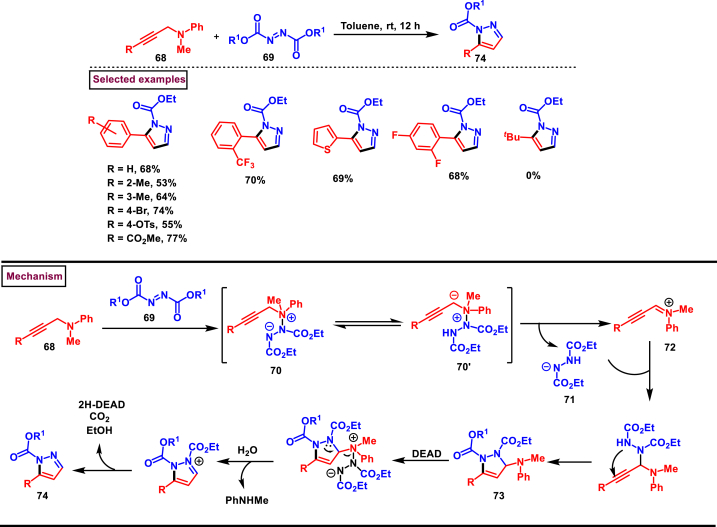
Synthesis of pyrazoles from propargylamines and dialkyl azodicarboxylates.

A phosphine and base free approach for the construction of pyrazole derivatives **(74)** was realized from functionalized propargylamines **(68)** and dialkyl azodicarboxylates **(69)** *via* [3 + 2] cycloaddition process by Jia et al. (Scheme 14) [51]. This process utilized readily available substrates and proceeded at room temperature to yield diverse range of pyrazole derivatives. Presence of electron withdrawing substituents on the *N*-atom led to no desired products. Wide range of aryl groups bearing sensitive functional groups such as, (-Cl, -Br, -CO_2_Me, -CF_3_, -OTs) were studied and the corresponding pyrazoles **(74)** were obtained in moderate to good yields. Alkyl substituted propargylamines were not reacted under this conditions. Mechanistically, nucelophilic addition of propargylamine **(68)** to DEAD **(69)** resulted in intermediate **(70)** which upon intramolecular H-abstraction yielded **(72)**. Imine cation **(72)** and 1H-DEAD **(71)** were generated *via* N-N bond cleavage. Nucleophilic reaction of imine cation with **(71)** followed by an intramolecular cyclization yielded the pyrazoline **(73)**. Further nucleophilic addition of DEAD, protonation and decarboxylation furnished the desired pyrazole **(74)**.

**Scheme 15:**
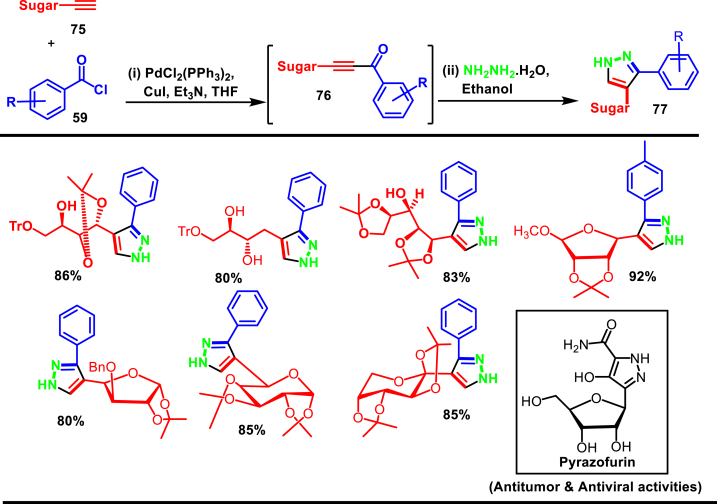
Pd-catalysed synthesis of aryl pyrazole analogues of pyrazofurin.

Zhang et al. disclosed a Pd-catalysed convenient method to synthesize aryl pyrazole analogues of pyrazofurin (**77**) *via* a one-pot coupling of sugar bound alkyne (**75**), hydrazine hydrate and acid chlorides (**59**) (Scheme 15) [52]. Broad range of examples (62) have been reported under mild conditions. Various alkynes with sugar moieties (**75**) such as furanosides, pyranosides and acyclic glycosides with sensitive protecting groups (benzyl, methyl, isopropylidene, triphenylmethyl) were participated in the reaction to provide the corresponding aryl pyrazole derivatives (**77**) in moderate to excellent yields. Authors have isolated a key intermediate **(76)** on a pre-completion stage of the reaction. Mechanistically, this intermediate **(76)** was formed from the alkyne precursor **(75)** *via* Pd/CuI-catalysed benzoylation, which further underwent cyclization with hydrazine to afford the sugar-bound pyrazole derivatives **(77)**. Notably, alkynes with free hydroxy groups, acyclic sugar units with sterically hindering functional groups were successfully screened. Electronic effects of substituted acid chlorides were also studied in detail.

**Scheme 16:**
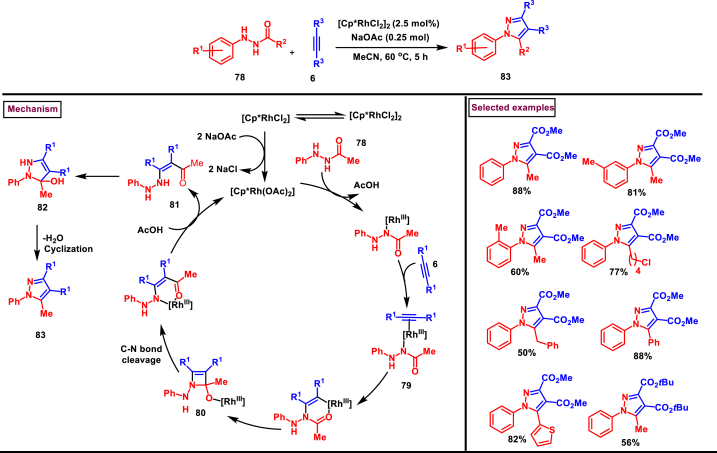
Synthesis of pyrazoles *via* Rh-catalysed cascade addition-cyclization.

A concise and efficient method to prepare synthetically useful and highly substituted pyrazole (**70**) was reported by Liu and co-workers *via* a Rh-catalysed cascade addition-cyclization sequence of hydrazine (**64**) with alkyne precursors (**65**) (Scheme 16) [53]. Hydrazines with various electron-donating substituents are well tolerated and yielded the corresponding pyrazoles (**70**) with excellent yields whereas, the substrates bearing electron-withdrawing groups are resulted in lower yields. On the other hand, symmetrically substituted alkynes reacted well with *N*′-phenylacetohydrazide **(64)** but the unsymmetrical counterparts were failed to react. Mechanistically, *in situ* generated active Rh-catalyst underwent alkyne coordination to afford key six-membered [C-Rh-O] complex **(66)**, which after a series of intramolecular nucleophilic addition **(80)**, ring opening (cleavage of C-N bond), protonation **(81)** and a final cyclization afforded the pyrazole product **(83)**.

### 3.2 Synthesis of pyrazoles from α,β-unsaturated carbonyl compounds

**Scheme 17:**
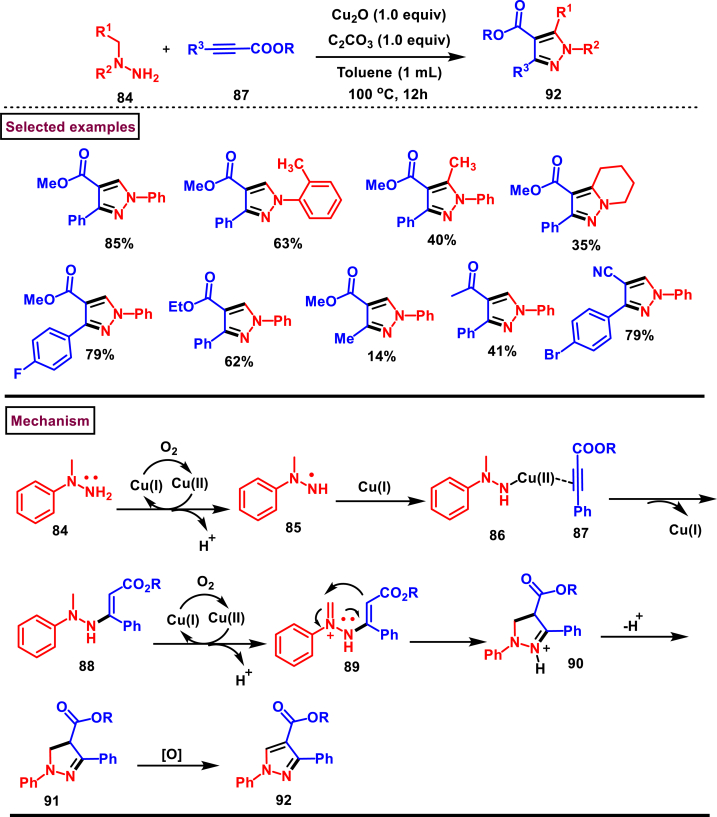
Synthesis of pyrazoles *via* Cu-catalysed oxidative [3 + 2] cycloaddition strategy.

Recently, Guo and Zheng et al. reported a Cu-catalysed oxidative [3 + 2] cycloaddition strategy to prepare multi-substituted pyrazoles (**92**) from the reaction of *N*, *N*-disubstituted hydrazines (**84**) and alkynoates (**87**) (Scheme 17) [54]. Inexpensive Cu_2_O and air are served as a promotor and green oxidant in this protocol. This aerobic oxidative approach involves C(sp^3^)-H functionalization and sequential formation of C-C/C-N bonds. Varieties of aromatic *N, N*-disubstituted hydrazines (**84**) with electronically distinct substituents furnished the pyrazoles (**92**) in good yields. Similarly, a range of alkynoates with 3-aryl group gave the desired products in good yields, however the aliphatic precursor (methyl but-2-ynoate) furnished the pyrazole in 14 % yield. Involvement of radical pathway was confirmed by radical trapping experiments. Mechanistically, single electron oxidation of hydrazine **(84)**, formation of Michael adducts **(88)**, 1,5-electrocyclization process **(90)**, deprotonation **(91)** and aerobic oxidation using oxygen as green oxidant were the key transformations involved in this process to deliver the pyrazole derivatives **(92)**. Wide substrate scope, high step and atom economy, excellent regioselectivity and operational convenience are the major advantages of this report.

**Scheme 18:**
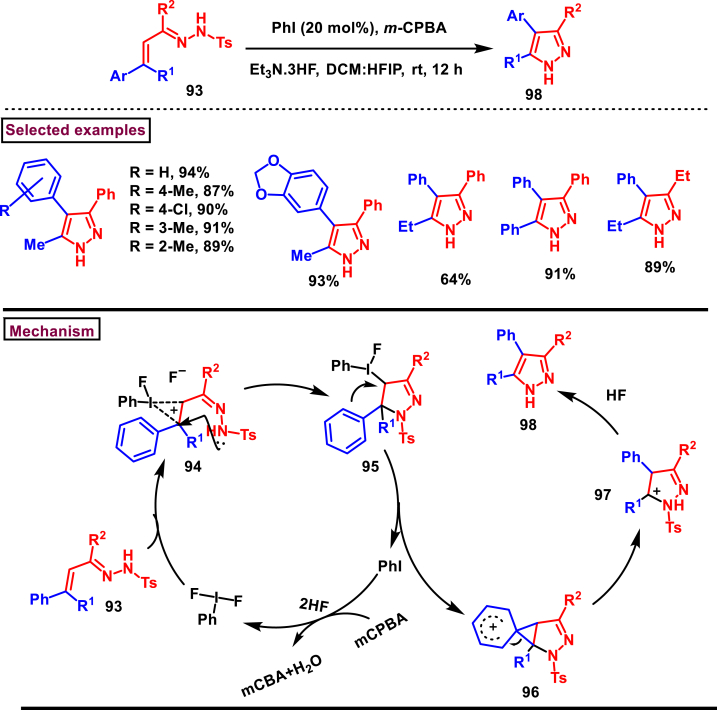
Iodine (III)-catalysed synthesis of pyrazoles from α,β-unsaturated hydrazones.

Iodine (III)-catalysed synthesis of multisubstituted pyrazoles **(98)** was realized from α,β-unsaturated hydrazones **(93)** Tiwari et al. (Scheme 18) [55]. This transformation proceeded *via* sequential cyclization/neighbouring group assisted 1,2-aryl shift/aromatization and *N*-Ts deprotection. A series of hydrazone derivatives **(93)** with electron rich aryl ring were screened to afford the desired product **(98)** in excellent yields. Halogens, benzo[*d*] [1,3]dioxole, β-napththyl and α-napththyl groups at β-position were successfully studied. Substitution effect on the non-migrating group was also carried out, thus alkyl and aryl units were found suitable to afford the pyrazole **(98)**. Substitution at the hydrazone carbon revealed that, wide range of aryl and alkyl units were well tollerated under this condition and the corresponding products were obtained in good yields (74%–94 %). *In situ* generated PhIF_2_ from iodobenzene, mCPBA and TEA.HF reacted with hydrazone and provided the iodonium intermediate **(94)**. Subsequent intramolecular cyclization led to **(95)**. Aryl group assisted the reductive elminiation of PhI and led formation of phenonium ion **(96)** which after aromatization and TEA.HF mediated N-Ts deprotection gave the pyrazole **(98)**. In addition, this method enabled the direct syntehsis of intermediates of Valdecoxib and Parecoxib (COX-inhibitory drugs). A parellel synthesis isoxazole was also discussed in this report.

**Scheme 19:**
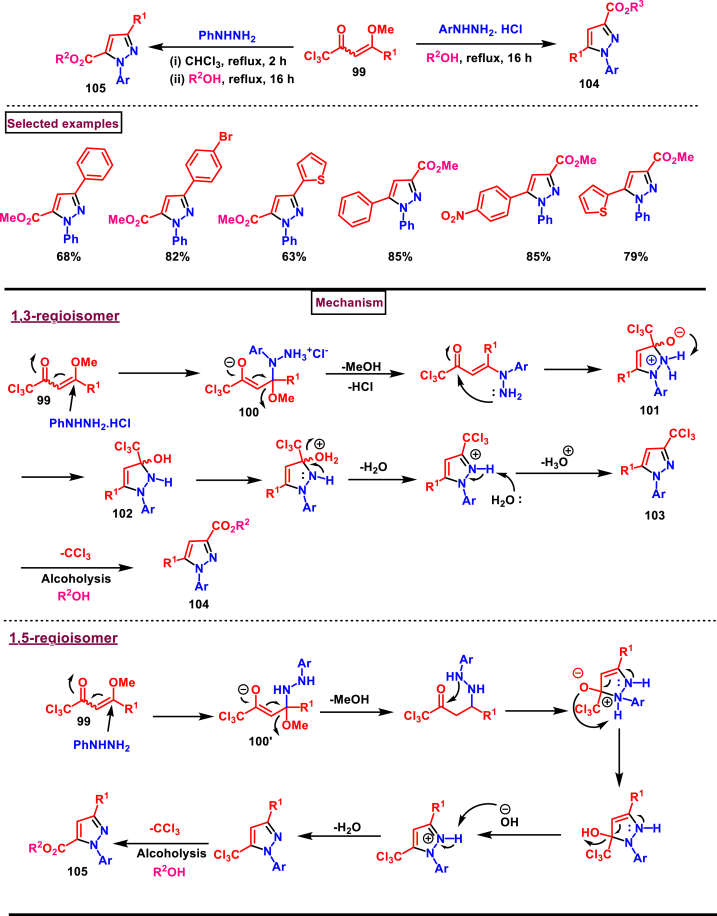
Regiocontrolled synthesis of 1,3- and 1,5-carboxyalkyl-1*H*-pyrazoles enones and arylhydrazines.

A regiocontrolled synthesis of 1,3- and 1,5-carboxyalkyl-1*H*-pyrazoles **(104** and **105)** were prepared from enones **(99)** and arylhydrazines (Zanatta et al. 2023) (Scheme 19) [56]. When the reaction was performed with arylhydrazine hydrochloride in methanol, 1,3-isomer **(104)** was obtained whereas, free hydrazine led to 1,5-isomer **(105)** in chloroform. CCl_3_-group in the enone served as a carboxyalkyl precursor. Variously substituted aryl and heteroaryl groups at β-position of the enone substrate **(99)** led to the desired 3-carboxyalkyl isomer **(104)** in good yields. Notably primary and secondary alkyl units were also found compatible. Different ester functionalities could be derived by employing alkyl, allyl and benzyl alcohols. Alkyl hydrazines were not reactive under this condition. On the other hand, synthesis of *N*-phenyl-3-aryl-1*H*-pyrazole-5-carboxylates was carried out with differently functionalized enones **(99)**. Electronic nature of the substituents (-OMe, -NO_2_, -Br) on the aryl ring of enone did not have any influence on the yield. Similarly, alcoholysis of trichloroacyl groups was performed with different alcoholic solvents and the desired 1,5-regioisomer **(105)** was obtained in good yields. When arylhydrazine hydrochloride was utilized, the most hindered nitrogen attcked the β-position of the enone and gave the intermediate **(100)**. Rapid elimination of methanol followed by a intramolcular cyclization provided the intermediate **(101)**. Further intramolecular H-abstraction resulted in pyrazoline intermediate **(102)**. Sequential dehydration and methanolysis of 3-trichloromethylpyrazole furnished the 1,3-regioisomer **(104)**. In the case of 1,5-regioisomer formation, least hindered nitrogen of arylhydrazine attcked the β-position of the enone and gave the intermediate **(100’)**. Desired product **(105)** was formed *via* the remaining steps discussed for the 1,3-regioisomer.

**Scheme 20:**
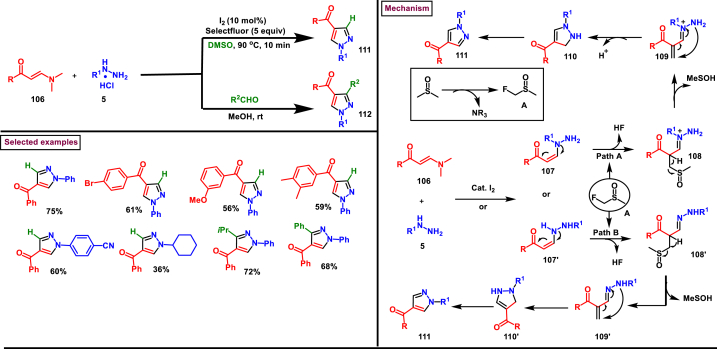
I_2_/Selectfluor mediated synthesis of pyrazoles using DMSO as C_1_-source.

I_2_/Selectfluor mediated strategy to prepare 1,4-disubstituted pyrazoles (**111**) was realized by Wan et al. (2022) from the cascade reaction of hydrazines (**5**), enaminones (**106**) and DMSO (Scheme 20) [57]. A dual role was played by DMSO as a solvent medium and C_1_-source. Additionally, a three-component synthesis of 1,3,4-trisubstituted pyrazoles (**112**) was also reported by using aldehydes as C_1_-source at room temperature. It was found that, no product was observed when I_2_ alone was used. However, the additive selectfluor led to increased yield. Enaminones bearing aryl/heteroaryl groups with varieties of substituents **(106)** afforded the corresponding 4-benzoyl pyrazoles **(111)** in moderate to good yields. Interestingly, enamino ester also took part in this transformation. Similarly, a series of aryl/alkyl hydrazines **(5)** were also screened to provide the corresponding products in moderate yields. The synthetic space was further explored by treating plethora of aldehydes as C_1_-sources and structurally diverse 1,3,4-trisubstituted pyrazoles were isolated with excellent outcome. Rh-catalysed alkenylation and fused aromatic products were prepared to extend the synthetic potential. Deuterium labelling experiments proved that DMSO served as C_1_-source and the radical pathway was ruled-out by TEMPO experiment. Two possible pathways (A and B) were proposed accordingly, I_2_-promoted transamination between hydrazine **(5)** and enaminone **(106)** resulted in the isomeric intermediates (**107** and **107′**) which reacted instantly with fluorinated DMSO **(A)** at the α-carbon to afford intermediates (**108** and **108′**). Further elimination of HSOMe and intramolecular amino addition to alkene bond generated pyrazolines **110** and **110’**. Desired product (**111)** was obtained after an oxidative aromatization.

**Scheme 21:**
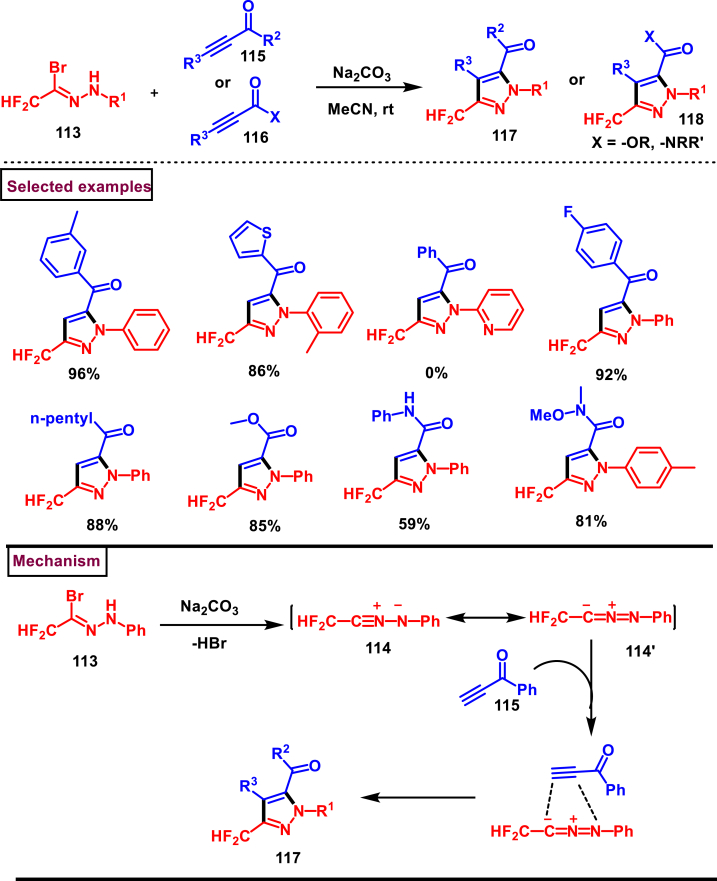
Regioselective synthesis of difluoromethyl pyrazoles *via* a [3 + 2] cycloaddition.

Hu et al. disclosed a simple method to access difluoromethyl pyrazoles (**117** and **118**) *via* a [3 + 2] cycloaddition of difluoroacetohydrazonoyl bromides (**113**) with various alkyne derivatives (**115** and **116**) such as, ynones, ynamides, and alkynoates under mild conditions in 2022 (Scheme 21) [58]. Excellent regioselectivity was observed and the desired products were obtained in moderate to good yields. Difluoroacetohydrazonoyl bromides **(113)** with electron-donating groups were resulted in the desired products in good yields, whereas the electron-withdrawing substituents affected the reaction progress and led to lower yields. Ynones with terminal alkyne **(115)** furnished the pyrazoles **(117)** in good yields whereas, the internal alkynes with aromatic or alkyl groups gave no desired product. Similarly, alkynoates with various alkoxy groups such as methoxy, phenoxy, *tert*-butoxy, 4-Me-phenoxy groups **(116)** gave the corresponding pyrazoles **(118)** in good yields. Though β-Me or β-Ph substituted alkynoates were not resulted in the desired products, dimethyl acetylenedicarboxylate afforded the multi-substituted pyrazole in good yield. In case of ynamides, aliphatic and aromatic amine derived propargyl amides (**87**) were well participated in the [3 + 2] cycloaddition sequence, however propynoic acid amide and β-substituted ynamides were not participated in the reaction. Mechanistically, base promoted formation of nitrile imine (**114** and **114’**) from difluoroacetohydrazonoyl bromides **(113)** followed by its reaction with alkyne unit **(115)** *via* [3 + 2] cycloaddition resulted in the pyrazole product (**117 or 118**).

**Scheme 22:**
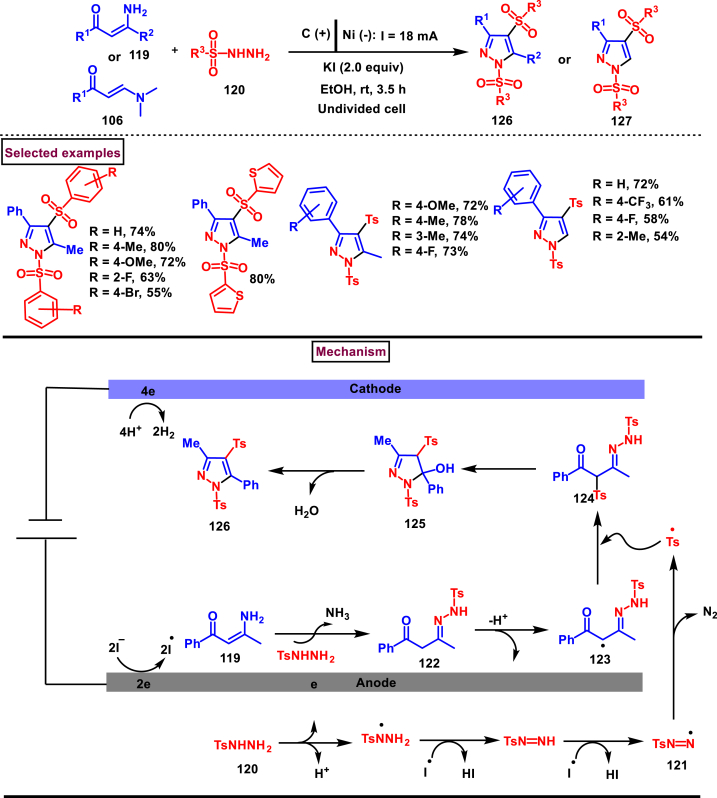
Electrochemical cascade synthesis of multi-functionalized pyrazoles from sulfonyl hydrazides and enaminones.

An electrochemical cascade approach for the synthesis of multi-functionalized pyrazoles **(126** and **127)** from sulfonyl hydrazides **(120)** and enaminones **(119** or **106)** was developed by Huang and Baell et al. (2022) (Scheme 22) [59]. Wide range of aryl sulfonyl hydrazides bearing electron releasing and withdrawing substituents (-Me, -OMe, -Cl) at various positions of the aryl ring were afforded the fully substituted pyrazoles **(126** or **127)** in good yields. In addition, poly-aryl, heteroaryl and alkyl sulfonyl hydrazides were also well participated in this transformation. On the other hand, enaminones bearing aryl/heteroaryl and alkyl (R^1^ = aryl, R^2^ = alkyl) substituents **(119)** were smoothly afforded the product **(126)** in acceptable yields. However, diaryl and dialkyl analogues were having poor reactivity yielding the product in trace amounts. Interestingly, steric effect of aryl substituents had no obvious impact on the yield. Notably, authors have also prepared 1,3,5-trisubstituted sulfonated pyrazoles **(127)** by utilizing N, N-dimethyl enaminone **(106)** as a precursor. A series of control studies and cyclic voltammetry experiments were conducted to gain mechanistic insights. Accordingly, at the anode surface iodide ions were oxidized into iodide radicals which further oxidized the sulfonyl hydrazide **(120)** into a hydrazide radical. A series of electrochemical oxidations resulted in radical intermediate **(121)** which eliminated N_2_ and produced Ts radical. On the other hand, intermediate **(122)** was obtained from the condensation of enaminone **(119)** and sulfonyl hydrazide **(120)** followed by an oxidation and deprotonation. Radical cross coupling between **(123)** and tosyl radical provided the di-tosyl intermediate **(124)** which underwent cyclization and dehydration to afford the pyrazole **(126)**.

**Scheme 23:**
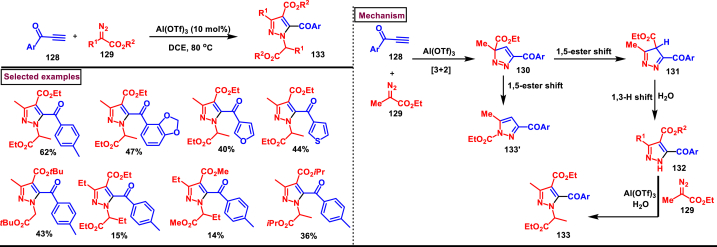
Al-catalysed synthesis of pyrazoles *via* [3 + 2] cycloaddition/rearrangement/N-H insertion sequence.

In 2021, Liu and Feng et al. described an Al(OTf)_3_ catalysed one-pot preparation of *N*-alkyl-3-alkyl-4-ester-5-benzoyl pyrazole derivatives (**133**) *via* [3 + 2] cycloaddition/rearrangement/N-H insertion sequence involving the reactions of α-diazoesters (**129**) and alkynones (**128**) with modest regioselectivity (Scheme 23) [60]. Wide range of terminal alkynone with benzoyl groups (**128**) were suitable for this transformation, however ynones with 3-furyl or 3-thiophenyl groups resulted in lower yield. On the other hand, α-diazoesters with bulky isopropyl or *tert-*butyl groups decreased the yield due to steric hindrance. Deuterium labelling studies, control experiments, kinetic studies were carried out and the plausible mechanism suggested that, a [3 + 2] cycloaddition between α-diazoacetate **(129)** and Lewis acid activated ynones **(128)** resulted in the formation of key intermediate **(130)**. 1,3-ester migration resulted in major isomer **(133)** and minor isomer **(133) was** obtained *via* 1,5-ester shift. H_2_O mediated intermolecular 1,3-*H* shift and subsequent Al(OTf)_3_ assisted N-H insertion furnished the desired *N*-alkyl pyrazole product **(133)**.

**Scheme 24:**
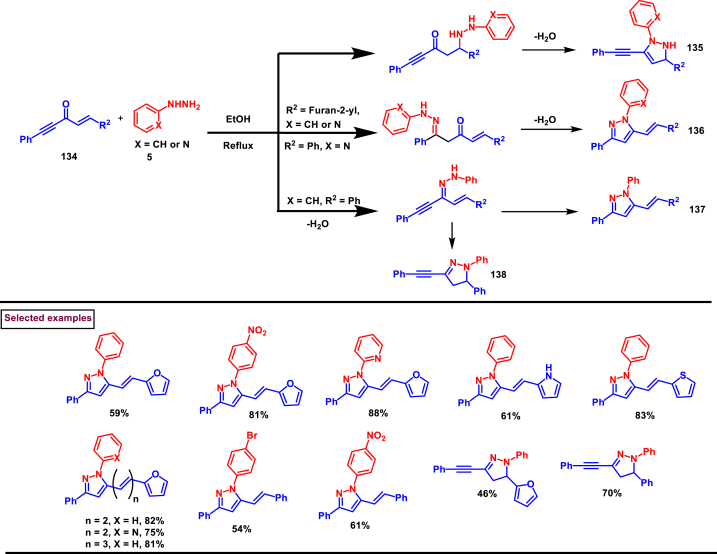
Substituent controlled synthesis of pyrazole derivatives *via* cyclo-condensation strategy.

In the same year, Golovanov et al. reported a substituent controlled cyclo-condensation strategy of cross-conjugated alkynones (**134**) with arylhydrazines (**5**) for the regioselective preparation of pyrazole (**136** or **137**) or 4,5-dihydro-1*H*-pyrazole derivatives **(135** or **138)** in good yield (Scheme 24) [61]. Reaction pathway is decided by the substituents at 1 and 5 positions of alkynone derivatives **(134)**. When the substituents are identical, 4,5-dihydro-1*H*-pyrazole (**135**) is the dominant product whereas, in the presence of substituted alkene and terminal alkyne unit cyclo-condensation occurs at terminal alkyne part and resulted in pyrazole derivatives (**136** or **137**). Various substituents both in alkynone (**134**) and hydrazine derivatives (**5**) were tolerated well and resulted in the corresponding products in good yields under simple conditions. In addition, the pyrazole products possess excellent fluorescent properties.

**Scheme 25:**
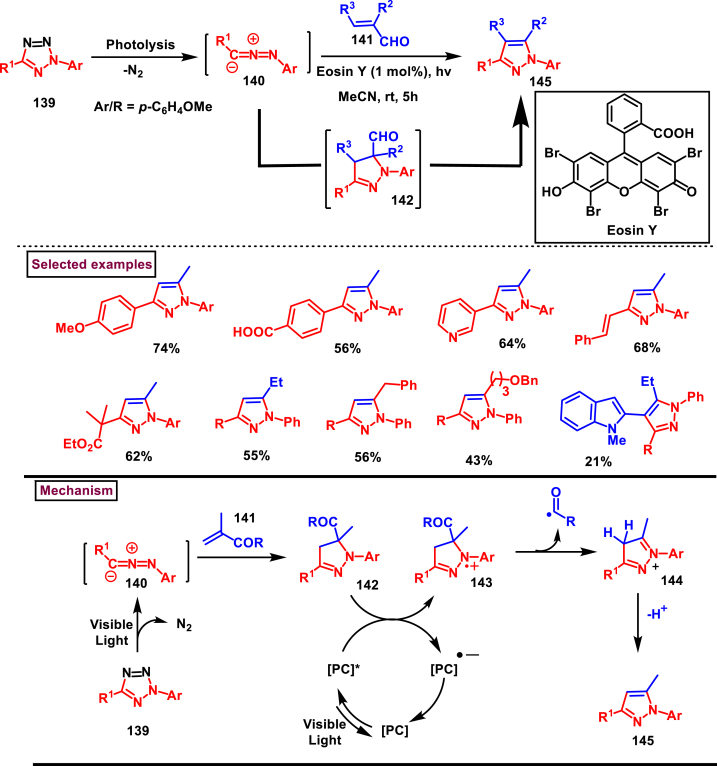
Synthesis of pyrazoles *via* photoclick 1,3-dipolar cycloaddition/Norrish type deformylation sequence.

An innovative domino method to synthesize pyrazoles (**145**) from 1,3-diaryltetrazoles (**139**) and α,β-unsaturated carbonyl derivative (**141**) *via* a photoclick 1,3-dipolar cycloaddition/Norrish type deformylation sequence has been realized by Ribagorda and Adrio et al. in 2021 (Scheme 25) [62]. An α,β-unsaturated aldehyde (**141**) was used as an alkyne synthetic equivalent in which the aldehyde group served as clean photoremovable directing group. Unprecedented photo-redox catalysed deformylation under visible light irradiation is the notable feature of this report. Based on the mechanistic insights, an initial photolysis of 1,3-diaryltetrazole (**139**) resulted in the *in situ* generation of dipole nitrile imine **(140)** which reacted with carbonyl precursor (**141**) *via* 1,3-dipolar cycloaddition and furnished pyrazoline (**142**). An electron (N1) is accepted by excited state oxidant and resulted in the formation of aminyl radical cation **(143)**. Further generation of formyl radical *via* C-C bond fragmentation, aromatization and oxidation of photocatalyst by oxygen completed the catalytic cycle. Utility of 3-methyl-3-buten-2-one resulted the desired product **(145)** only in 30 % yield, whereas the other α,β-unsaturated carbonyl derivatives such as, carboxylic acid, ester, amide yielded only the pyrazolines (**142**) due to the absence of carbonyl fragmentation.

**Scheme 26:**
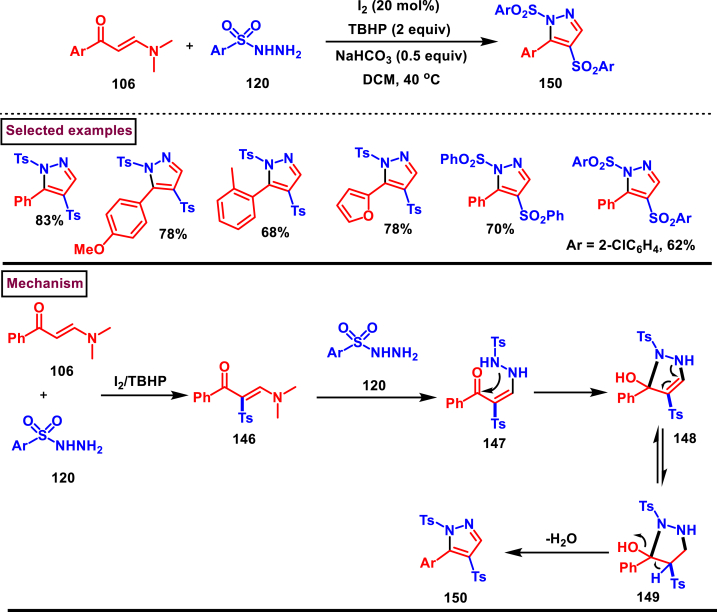
Synthesis of pyrazoles *via* I_2_/TBHP-medidated annulation of sulfonyl hydrazines and enaminones.

I_2_/TBHP-medidated annulation of sulfonyl hydrazines **(120)** and enaminones (**106**) resulted in the formation of 3-functionalzied pyrazoles (**150**) *via* tandem C(sp^2^)-H sulfonylation (Sheng and Wan et al. 2020) under transition metal-free conditions (Scheme 26) [63]. Diverse range of aryl based N, N-dimethyl enaminones (**106**, alkyl, alkoxy, halogens, NO_2_) were smoothly participated, however alkyl analogue was not involved in this transformation. Acceptable functional group tollerance was exerted with respect to sulfonyl hydrazine precursors (**120**). TEMPO-based control experiments clearly proved the free radical C-H sulfonylation. Importantly, the tosyl group conveniently dissociated to afford pyrazoles (**150**) with free -NH group showcasing the synthetic potential of this method. In this process, initial formation of sulfonylated enaminone intermediate **(146)** was observed from the I_2_ catalysed radical C-H sulfonylation. Further transamination of **(146)** yielded the intermediate **(147)**. Intramolecular cyclization **(148)** and subsequent isomerization **(149)** and dehydrative elimination furnished the desired pyrazole **(150)**.

**Scheme 27:**
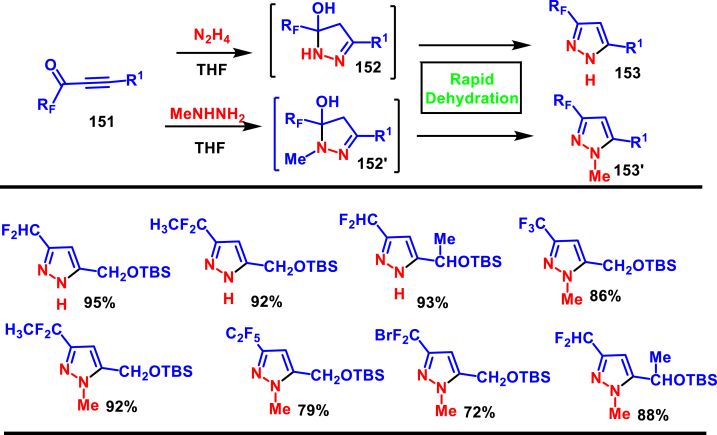
Regioselective synthesis of 3-fluoroalkylated pyrazole derivatives.

In 2019, Grygorenko and co-workers reported a facile synthetic method to prepare 3-fluoroalkylated pyrazole derivatives (**153** and **153′**) with functionalization side chain from the reaction of 1-fluoroalkyl substituted ynones (**151**) and N_2_H_4_ or MeNHNH_2_ (Scheme 27) [64]. This method exhibited excellent regioselectivity and afforded the corresponding products in good yields. The minor isomer (5-fluoroalkyated pyrazoles) was easily separated by a column chromatography or distillation. Prior to workup, 5-hydroxypyrazoline intermediate (**152** and **152′**) was present in the reaction mixture, however after workup, concentration and a silica-filtration led to rapid dehydration and resulted in final products (**153** and **153’**). Additionally, this method also described the synthesis of 3 or 5-fluoroalkylated isoxazoles from the reaction of fluoroalkyl substituted ynones (**151**) and NH_2_OH. In addition to the synthesis of diverse range of pyrazoles, preparation of fluorinated Mepiprazole analogues and multi-gram scale synthesis were demonstrated.

**Scheme 28:**
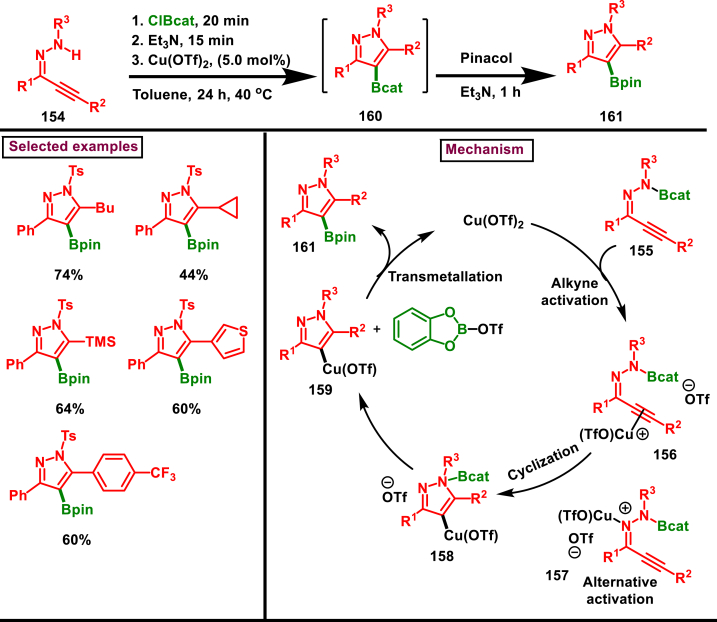
Synthesis of borylated pyrazoles *via* Cu-catalysed aminoboration and borylative cyclization.

A series of borylated pyrazoles (**161**) were prepared *via* Cu-catalysed aminoboration and borylative cyclization (Blum et al. 2019) employing readily available hydrazone derivatives (**154**) (masked α,β-unsaturated carbonyl) (Scheme 28) [65]. Initially, B-N σ-bond formation was realized by a reaction of B-chloro-catecholborane and hydrazone derivative **(154)** in presence of TEA. This intermediate was converted into borylated pyrazoles **(160)** in presence inexpensive Cu(OTf)_2_ *via* a intramolecular borylative heterocyclization which was trans-esterified into more stable pyrazole pinacol boronic esters **(161)**. Variously substituted aryl rings in the hydrazone precursors (**154**) were yielded the desired products (**161**) including sensitive bromo group. However, replacing tosyl functionality into phenyl group and substrates with enolizable protons were failed to give the desired borylated pyrazoles. Proposed mechanism described that, after the initial N-B σ-bond formation **(155)**, alkyne activation by Cu-catalyst **(156)** and intramolecular cyclization of organo-copper intermediate **(158)** was formed which underwent coupling with boron reagent to afford the desired borylated pyrazoles **(161)**.

**Scheme 29:**
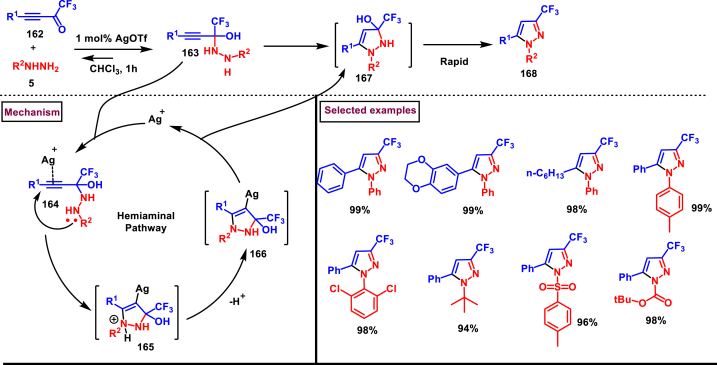
AgOTf-catalysed selective synthesis of 3-CF_3_ pyrazoles.

In 2018, Nenajdenko and co-workers reported a AgOTf-catalysed selective synthesis of 3-CF_3_ pyrazoles (**168**) from the reaction of trifluoromethylated ynones (**162**) and aryl/alkyl hydrazines (**5**) (Scheme 29) [66]. Variety of substituted pyrazoles were prepared in high yields with excellent regioselectivity for 3-CF_3_ isomer whereas, the 5-CF_3_ isomer was formed in <5 %. Broad substrate scope was observed for both ynones (**162**) and hydrazines (**5**). The synthetic utility of this report was demonstrated by preparing well-known pyrazole drugs such as, SC-560 and Celebrex. Mechanistic investigations suggested that, a rapid reversible generation of hemiaminal **(163)** and its Ag-catalysed intramolecular cyclization resulted in pyrazolinol **(167)** intermediate which after elimination of water yielded the 3-CF_3_-pyrazole derivative (**168**).

**Scheme 30:**
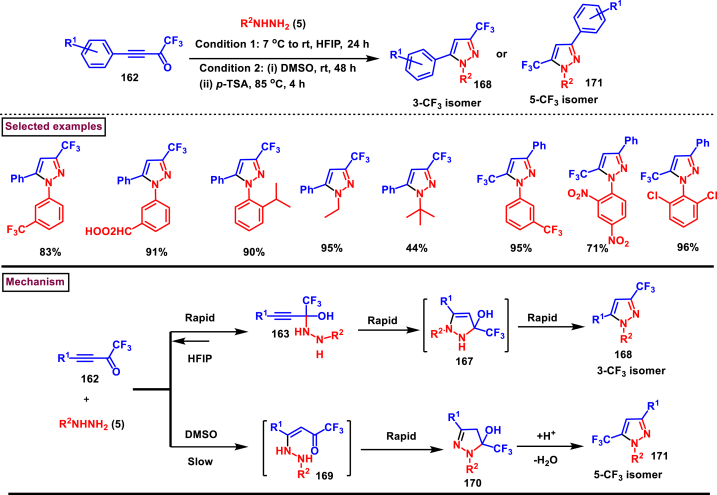
Solvent controlled synthesis of 3-CF_3_ pyrazoles and 5-CF_3_ pyrazoles.

In 2017, Nenajdenko and Rulev et al. reported a solvent controlled approach to prepare both 3-CF_3_ pyrazole (**168**) and 5-CF_3_ pyrazoles (**171**) from the direct reaction of CF_3_-ynones **(162)** and aryl/alkyl hydrazines **(5)** (Scheme 30) [67]. 3-trifluoromethylpyrazoles **(168)** were exclusively formed in hexafluoroisopropanol which is a highly polar protic solvent. On the other hand, polar aprotic solvent (DMSO) resulted in the formation of 5-CF_3_ pyrazoles **(171)**. Both ynones **(162)** and hydrazines **(5)** bearing a range of substituents and functional groups were evaluated under optimal conditions. Electronic nature of arylhydrazine played a crucial role when the reaction was carried out in HFIP solvent. Thus, more electron-withdrawing substituents (2,4-dinitrophenyl, polyfluorophenyl, 4-aminosulfonyl) led to the selective formation of 5-CF_3_ isomer **(171)** whereas, highly nucleophilic aryl/alkyl hydrazines **(5)** resulted in 3-CF_3_ isomer **(168)**. The flexibility and synthetic utility of this method was further demonstrated by preparing pyrazole-based drug molecules such as, Celebrex and SC-560 and their regioisomers. Reaction of phenylhydrazine **(5)** with alkynone substrate **(168)** in HFIP proceeded with a nucleophilic addition at carbonyl center and resulted in rapid formation of hemiaminal **(163)**. Subsequent intramolecular cyclization and dehydration yielded the 3-CF_3_ isomer (**168**). In DMSO, the hemi-aminol intermediate **(163)** dissociated into substrates and their slow recombination resulted in enone **(169)**. Further cyclization **(170)** and dehydration under proton source yielded the 5-CF_3_ isomer **(171)**.

**Scheme 31:**
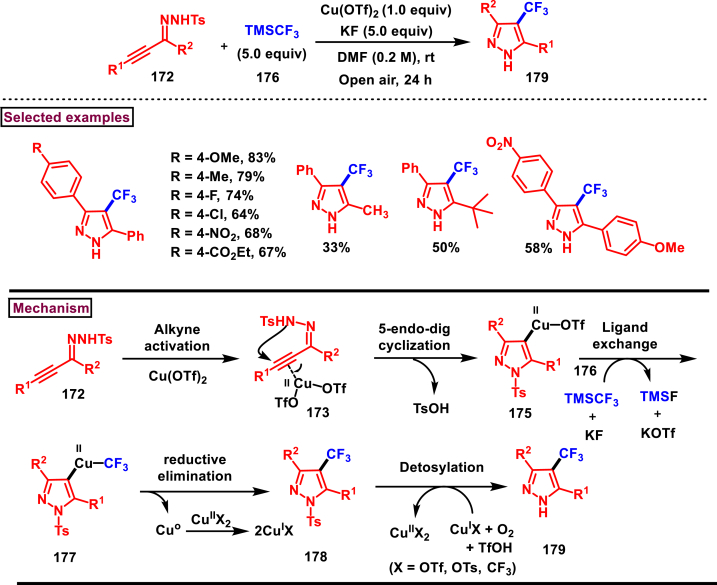
Synthesis of 4-(trifluoromethyl)-pyrazoles from TMSCF_3_ and α,β-alkynic hydrazones.

Pharmaceutically important 4-(trifluoromethyl)-pyrazoles **(179)** were obtained from TMSCF_3_ **(176)** and α,β-alkynic hydrazones **(172)** (Tsui et al. 2017) (Scheme 31) [68]. This transformation proceeded through intramolecular 5-*endo-dig* cyclization, trifluoromethylation, and tosyl deprotection. Electron rich aryl groups connected to alkyne **(172)** were reacted efficiently than electronically poor counterparts. Naphthyl and heteroaryl groups were compatible under this condition and yielded the corresponding products **(179)** in good yields. However, alkyl substituents showed poor reactivity. Substituent effects at the hydrazone carbon were also studied with electronically distinct aryl rings. Alkyl groups were shown lower reactivity than aryl groups. To demonstrate the practical utility, a three-step synthesis of 4-CF_3_ analogue of Celecoxib (anti-inflammatory drug) was performed with overall 37 % yield. Complexation of Cu-catalyst to the alkyne unit resulted in intermediate **(173)**. Intramolecular 5-*endo-dig* cyclization gave the 4-Cu-pyrazole **(175)** with the simultaneous elimination of TfOH. Ligand exchange process in the presence of TMSCF_3_ and KF provided **(177)** which upon reductive elimination Cu(0) afforded the 4-CF_3_-*N*-Ts-pyrazole **(178)**. Upon the oxidation of Cu(0) to Cu(II) in presence of O_2_ and Cu(II), detosylation occurred *via* SET process to furnish the desired pyrazole **(179)**.

### 3.3 Synthesis of pyrazoles from diazo compounds

Diazo compounds are one of the important building blocks in organic synthesis [69,70]. Due to the presence of C-N-N bond, it is often called as synthon of pyrazole core. Several research groups reported a series of [3 + 2] cycloaddition methods involving diazo compounds and alkenes, allenes, alkynes, nitroolefins etc. Hence it is noteworthy to organize some key reports for the synthesis of pyrazole core involving both diazo precursor and/or intermediates.

**Scheme 32:**
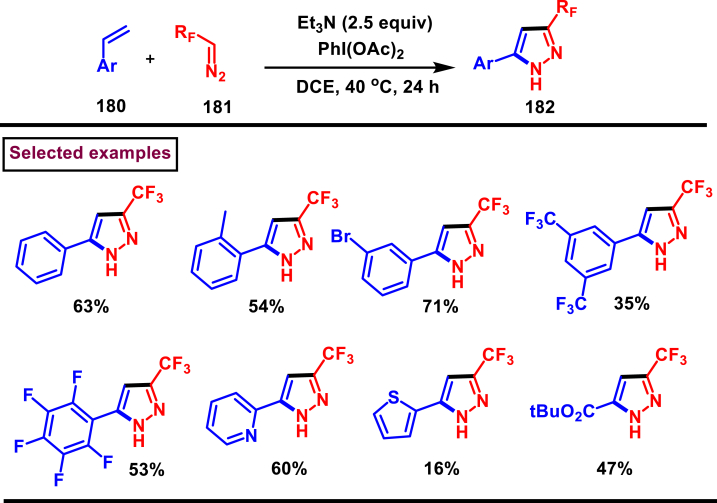
Synthesis of pyrazoles *via* [3 + 2] cycloaddition-isomerization-oxidation sequence.

Recently, Carreras et al. reported a facile method to access 5-aryl-3-trifluoromethylpyrazole (**182**) from styryl derivatives (**180**) and 2,2,2-trifluorodiazoethanes (**181**) *via* a [3 + 2] cycloaddition-isomerization-oxidation sequence (Scheme 32) [71]. This one-pot, metal-free protocol furnished the desired product in presence of Et_3_N and PhI(OAc)_2_ *via* the intermediacy of pyrazoline. Arylacetylene led to lower conversion compared to styrene however, alkyl and disubstituted alkenes gave no product. For the obtained pyrazoles (**182**) a set of post synthetic functionalization such as, *p*-bromination of the aryl group, N-alkylation and arylation were successfully carried out.

**Scheme 33:**
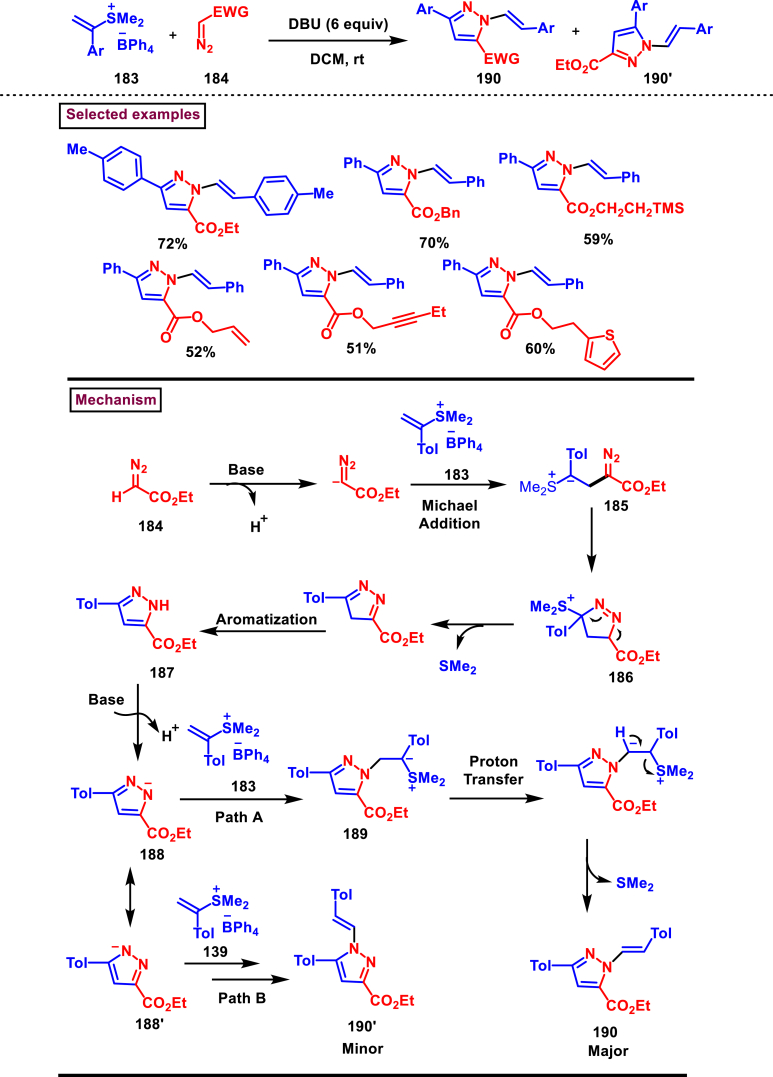
Synthesis of N-vinyl pyrazoles *via* [3 + 2] cycloaddition of vinyl sulfonium salts.

A DBU promoted regioselective synthesis of *N*-vinyl pyrazoles **(190 and 190′)** was reported from vinyl sulfonium salts **(183)** and diazo compounds **(184)** by Li et al. (2023) (Scheme 33) [72]. This metal free approach was proceeded through a [3 + 2] cycloaddition of vinyl sulfonium salts (**183**) and diazo anions followed by *N*-vinylation. Scope of α-aryl vinyl sulfonium salts and β-aryl vinyl sulfonium salts with electron-donating and electron withdrawing substituents worked well under this condition. However, sterically demanding substituents resulted in lower yield and poor regioselectivity. Synthetic potential of this method was showcased by a gram scale synthesis and conversion of *E*-isomer into *Z*-isomer in the presence of fac-Ir(ppy)_3_ (10 mol%). Based on the mechanistic investigation, DBU mediated deprotonation followed by a Michael addition of diazo anion with **(183)** resulted in sulfur ylide **(185)**. Intramolecular cyclization **(186)**, elimination of Me_2_S and further aromatization led to pyrazole **(187)**. In presence of DBU, two isomeric *N*-anions were generated **(188** and **188′)**, in which reaction of **(188)** with **(183)** generated ylide **(189)** which underwent elimination of Me_2_S and furnished the pyrazole **(190)** as a major product. On the other hand, isomer **(188′)** led to minor isomer **(190’)**.

**Scheme 34:**
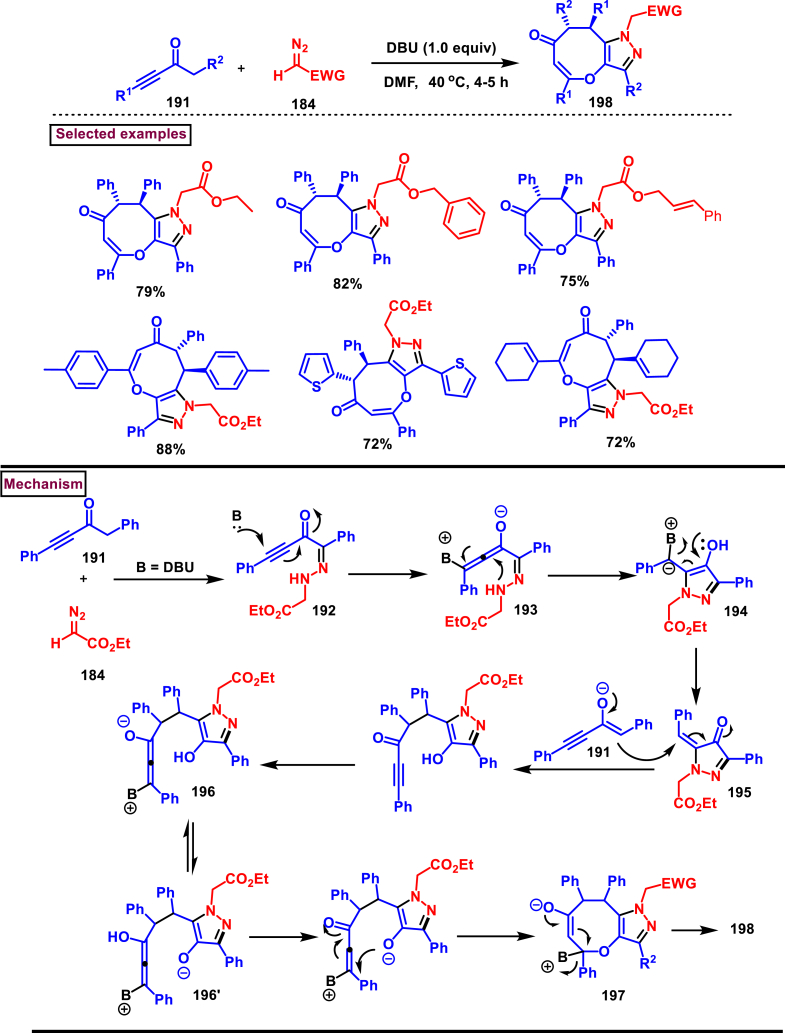
Synthesis of fused oxocino[2,3-*c*]pyrazoles *via* DBU-promoted cyclization.

Zhao et al. reported an elegant and challenging DBU -promoted cyclization method to prepare fused oxocino[2,3-*c*]pyrazoles (**198**) from ynones (**191**) and ethyl diazoacetate (**184**) (Scheme 34) [73]. This tandem process proceeded with excellent regio- and diastereoselectivity from easily available acyclic precursors in a single step. Diazo compounds (**184**) served as N-terminal electrophiles in this domino process with consecutive formation of four new bond and two rings. Broad range of functional groups in substituted ynones (**191**) and α-diazocarbonyl compounds (**184**) including electronically distinct functional group on the aryl group were compatible and resulted the desired products (**198**) in good yields. Mechanistic rational suggested that, hydrazone intermediate **(192)** generated *via* the DBU-assisted nucleophilic addition between **(184)** and **(191)** was converted into zwitterionic species **(193)**. Intramolecular aza-Michael addition and sequential protonation, tautomerization and DBU-elimination resulted the intermediate **(195)**. Intermolecular conjugate addition of **(191)** to **(195)** followed by a oxa-Michael addition gave the zwitterionic species **(196** and **196’)**. Finally, cyclization and elimination of DBU afforded the desired product **(198)**. Intramolecular aza-Michael addition and oxa-Michael addition were proposed to be the key steps which simultaneously allow the generation of two C-N bonds, one C-O and C-C bonds with high regio and stereoselectivity.

**Scheme 35:**
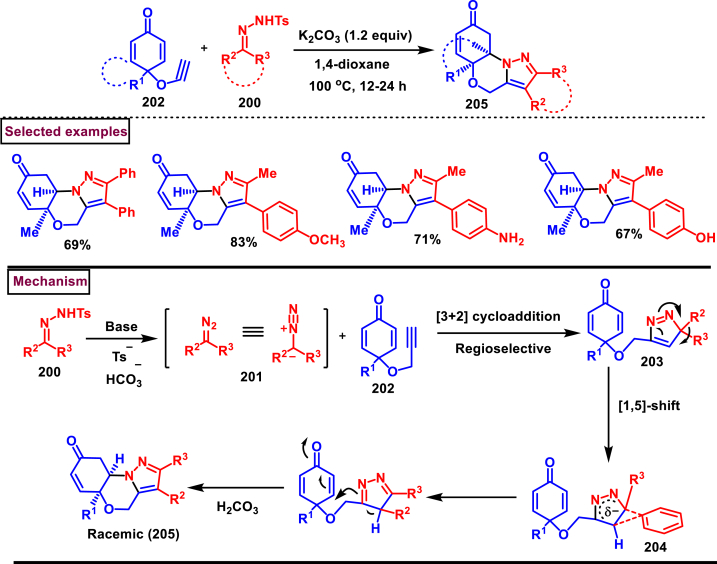
Synthesis of polycyclic-fused pyrazoles from alkyne tethered cyclohexa-2,5-dienones and N-tosylhydrazones.

Earlier this year, Kumar et al. disclosed a straightforward approch for the synthesis of polycyclic-fused pyrazoles **(205)** from alkyne tethered cyclohexa-2,5-dienones **(202)** and *N*-tosylhydrazones **(200)** *via* a cascade process (Scheme 35) [74]. This transformation proceeded through [3 + 2] cycloaddition, sigmatropic shift then aza-Michael addition reaction. Varieties of symmetrical and unsymmeterical diaryl or alkyl aryl *N*-tosyl hydrazones with -OH and -NH_2_ group participated in this process and the corresponding products **(205)** were obtained in good yields. However, dialkyl (acyclic or cyclic) analogues led to no desired products. (+)-carvone derived diazo substrate was also viable and the desired product was isolated as a diasteromeric mixture (dr = 1:1). On the other hand, 3,4-dimethyl substituted and cyclopentyl fused alkyne tethered cyclohexa-2,5-dienones were also tested and the anticipated products **(205)** were isolated in moderate yields. A detailed mechanistic studies revealed that, base assisted *in situ* generation of diazo precurosr **(201)** from *N*-tosylhydrazone **(200)** regioselectively reacted with alkyne unit *via* [3 + 2] cycloaddition and provided the intermediate **(203)**. Selective [1,5]-shift of aryl group resulted in intermediate **(204)**. Sequential aza-Michael addition and aromatization yielded the tricyclic fused pyrazole **(205)**. Similar mechanistic elucidations were provided for diazo substrates of aldehyde hydrazones.

**Scheme 36:**
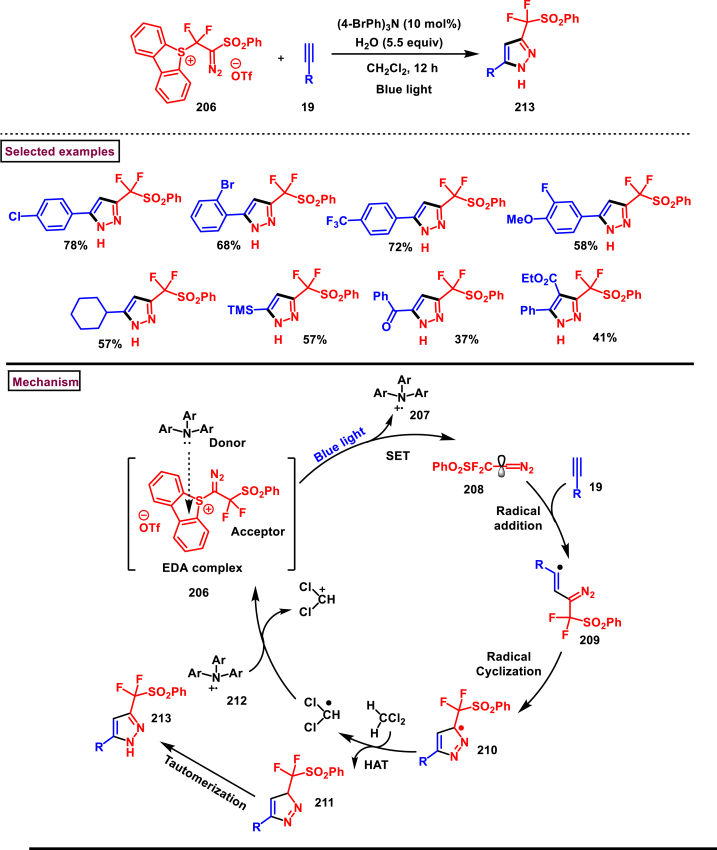
Synthesis of difluoromethylated pyrazoles from unactivated alkynes and α-diazodifluoroethyl sulfonium reagent.

A radical cyclization approach to prepare difluoromethylated pyrazoles **(213)** from unactivated alkynes **(19)** and α-diazodifluoroethyl sulfonium reagent **(206)** under blue light mediated [3 + 2] cycloaddition conditions (Zhang et al. 2023) (Scheme 36) [75]. Mono and disubstituted unactivated aryl alkynes substituted with electron rich (-Me, -OMe, -*t*Bu, Ph) and withdrawing substituents (-CF_3_, -CN, -NO_2_) and halogens (-Cl, -Br, -F) were successfully involved in this transformation and provided the desired products **(213)** in moderate yields. In addition, unactivated aliphatic alkynes **(19)** were also found viable substrates in this process. Tris(4-bromophenyl)amine was used as electron donor in this process. Electron donor-acceptor complex formation between amine and α-diazo sulfonium salt was crucial in this method. A trace amount of water facilitated the reaction by improving the solubility of α-diazo sulfonium salt **(206)**. Mechanistically, formation of EDA complex between amine and sulfonium salt followed by a SET generated difluorodiazomethyl radical **(208)**. Stable intermediate **(209)** was formed by the addition of radical **(208)** to alkyne **(19)** which rapidly underwent cyclization to afford pyrazolyl radical species **(210)**. Hydrogen atom transfer from CH_2_Cl_2_ followed by tautomerization provided the pyrazole **(213)**.

**Scheme 37:**
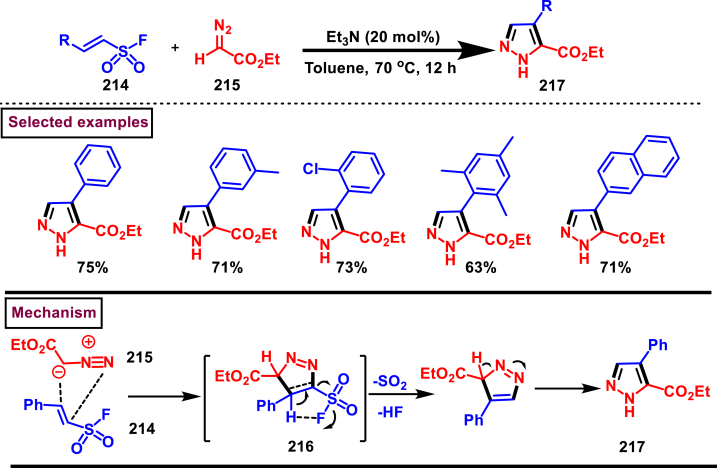
Synthesis of pyrazoles *via* TEA catalysed [3 + 2] thermal cycloaddition.

A set of pyrazole derivatives (**217**) were achieved *via* TEA catalysed [3 + 2] cycloaddition reaction of β-aryl vinyl sulfonyl fluorides (**214**) with ethyldiazoacetate (**215**) (Kumara Swamy et al. 2022) (Scheme 37) [76]. This transformation was initiated by a Michael addition diazo precursor (**215**, anionic form) on the β-carbon of vinyl sulfonyl fluorides (**214**) followed by SO_2_ elimination. Further isomerization of diazo product resulted the pyrazole **(217)**. Regardless of the substitution pattern and electronic nature of the aryl ring, vinyl sulfonyl fluorides were worked well and the desired pyrazoles (**217**) were obtained in good yields showcasing the versatility of this TEA catalysed [3 + 2] cycloaddition. Authors have also demonstrated the synthesis of 1,5-disubstituted triazoles *via* thermal [3 + 2] cycloaddition of β-aryl vinyl sulfonyl fluorides (**214**) with azides.

**Scheme 38:**
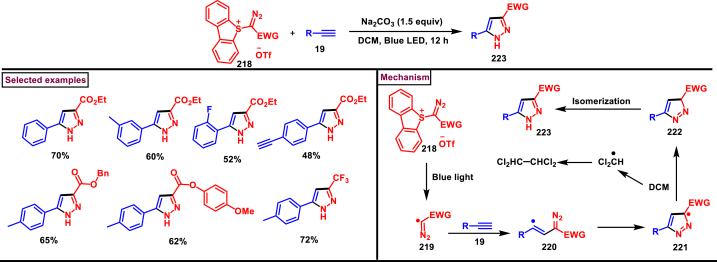
Synthesis of 3,5-substituted pyrazoles *via* visible light induced [3 + 2] cyclization.

In 2022, Zhou and Yuan et al. reported a visible light induced method to prepare 3,5-disubstituted-1*H*-pyrazoles (**223**) from the [3 + 2] cyclization of electron deficient α-diazo sulfonium salts (**218**) and alkynes (**19**) with excellent regioselectivity (Scheme 38) [77]. Interestingly, key intermediate diazomethyl radical **(219)** was generated *in situ* from α-diazosulfonium triflates (**218**) under photocatalyst, metal, and chemical oxidant free conditions *via* radical cleavage of α-diazo sulfonium salts. Various arylalkynes (**19**) with electronically distinct substituents resulted the desired pyrazoles **(223)** in moderate to good yields. However, aliphatic alkynes led to no desired products. A plausible mechanism to support the radical pathway was proposed and supported by various control experiments, deuterium labelling studies and DFT calculations. Accordingly, C-C bond formation between diazo radical and terminal carbon of the alkyne **(19)** followed by an intramolecular cyclization with a C-N bond formation **(220)** was realized. Further radical shift **(222)** and tautomerization yielded the pyrazoles **(223)**. This report also described the synthesis of 2,5-disubstituted 1,3,4-oxadiazoles from the cyclization of α-diazo sulfonium salts and α-oxocarboxylic acids.

**Scheme 39:**
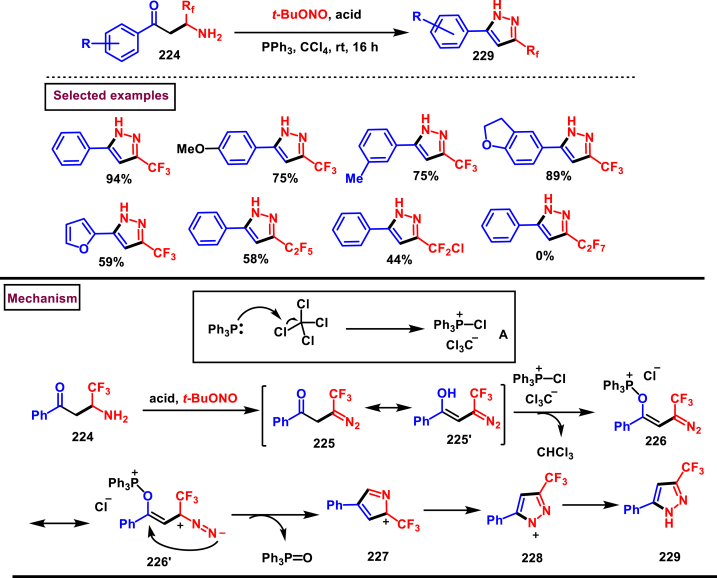
Synthesis of 3-CF_3_ pyrazoles *via* intramolecular Appel type reaction.

Han et al. (2022) reported a novel approach for the preparation of 3-CF_3_ pyrazoles (**229**) *via* an intramolecular Appel type reaction of CF_3_ bearing *β*-ketodiazo unit **(224)** and alkoxyphosphonium moiety (**A**) (Scheme 39) [78]. Gratifyingly, *β*-keto trifluoromethyl diazo entity **(225** and **225′)** was *in situ* generated from the reaction of amine precursor (**224**), *t*-BuONO and PPh_3_. Scope of the reaction was found to be fairly general with various aryl, heteroaryl amine precursors and fluoroalkyl units. Formation of diazo intermediate and the crucial role of PPh_3_ were confirmed by various control experiments and ^19^FNMR. Based on the results of these studies, a plausible mechanism was proposed for this intramolecular cyclization. Formation of intermediate **A** was realized from PPh_3_ and CCl_4_. Diazotization of amine precursor resulted in the tautomeric mixture **(225** and **225’)** which readily reacted with **A** to afford oxophophonium salt **(226)**. Further intramolecular cyclization and proton abstraction led to pyrazole **(229)**. Application of this transformation was further extended to prepare pyrazoles bearing natural product skeletons and a gram scale preparation was also demonstrated.

**Scheme 40:**
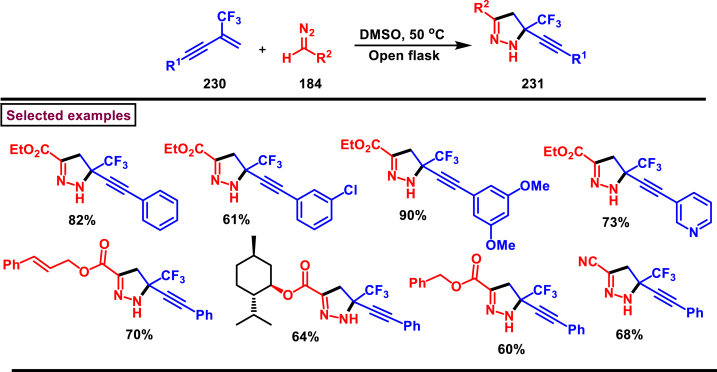
Synthesis of 2-pyrazolines from β-functionalized enyne and diazo compound.

2-Pyrazolines bearing an alkyne and CF_3_ group substituted quaternary carbon atom **(231)** was prepared from a β-functionalized enyne **(230)** and diazo compound **(184)** under transition metal-free mild conditions (Li and Hou et al. 2022) (Scheme 40) [79]. Irrespective of the electronic nature, various aryl and heteroaryl substituted 1,3-enynes were smoothly participated in this process and the anticipated pyrazolines **(231)** were obtained in good yields (53%–88 %). Similarly, diazo precursors bearing sensitive functionalities such as, alkynyl, benzyl, allyl, nitriles and cyclopentyl were survived under the reaction conditions. Importantly, few naturally derived diazoacetates were also involved in this transformation and successfully yielded the products **(231)**. Open flask conditions, broad substrate scope and mild conditions are the notable features of this report.

**Scheme 41:**
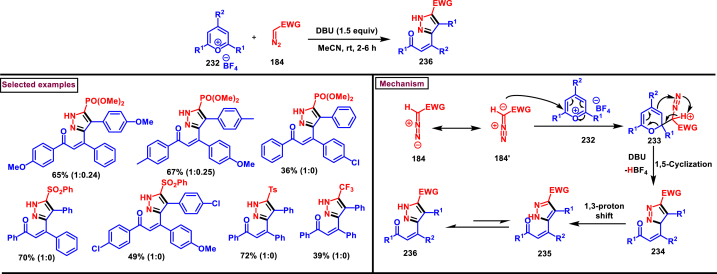
Base mediated synthesis of functionalized pyrazole chalcones.

In 2021, Rastogi et al. developed a base mediated approach for the synthesis of functionalized pyrazole chalcones (**236**) from α-diazo compounds tethered electron withdrawing groups (**184**) and 2,4,6-triaryl/alkyl substituted pyrylium salts (**232**) (Scheme 41) [80]. Sulfones, phosphonates, trifluoromethanes were utilized as electron withdrawing units. Desired products were formed as an inseparable mixture of 3-P-5 and 5-P-5 tautomeric mixture. Gratifyingly, X-ray analysis confirmed that, pyrazole (**236**) exists exclusively as a 5-P-5 tautomer in solid state and chalcone moiety adopts the Z-configuration. A fairly general scope was shown by variety of substituted pyrylium salt (**232**) and α-diazo compounds (**184**). Mechanistic analysis shown that, pyrylium salt **(232)** underwent nucleophilic attack by diazomethyl carbon **(184’)** and exclusively generated 2-diazomethyl pyran isomer (233) which upon further DBU-mediated α-deprotonation, C-O bond cleavage and 1,5-cyclization resulted in pyrazole (**236**) formation. Pyrazole chalcones (**236**) were obtained *via* 1,3-proton shift. Interestingly, the end products were converted into indeno-pyrazoles upon a hydride reduction and acidic workup through a Nazarov-type cyclization.

**Scheme 42:**
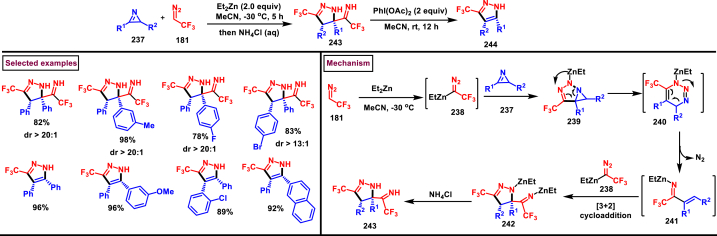
Synthesis of pyrazoles Zn/PhI(OAc)_2_ -mediated annulation-oxidation sequences.

In 2021, Ma and Zhang et al. reported an unconventional synthetic route to access wide range of pyrazolines (**243**) *via* zinc promoted annulation reactions involving 2*H*-azirines (**237**) and 2,2,2-trifluorodiazoethane (**181**) (Scheme 42) [81]. This method involves two sets of [3 + 2] cycloadditions, a ring opening and a nitrogen elimination step. Notably, the pyrazolines (**243**) are obtained with excellent diastereoselectivities and converted into pyrazoles (**244**) by treating with PhI(OAc)_2_ under mild conditions. Broad substitution patterns in 2*H*-azirines (**237**) were tolerated during this transformation and afforded the corresponding pyrazolines (**243**). Mechanistically, Et_2_Zn assisted formation of zinc trifluorodiazoethylide **(238)** followed by its [3 + 2] cycloaddition with 2*H*-azirine **(237)** gave the intermediate **(239)**. Ring opening **(240)** and N_2_-extrusion resulted in intermediate **(241)**. Desired product **(243)** was obtained *via* another set of [3 + 2] cycloaddition.

**Scheme 43:**
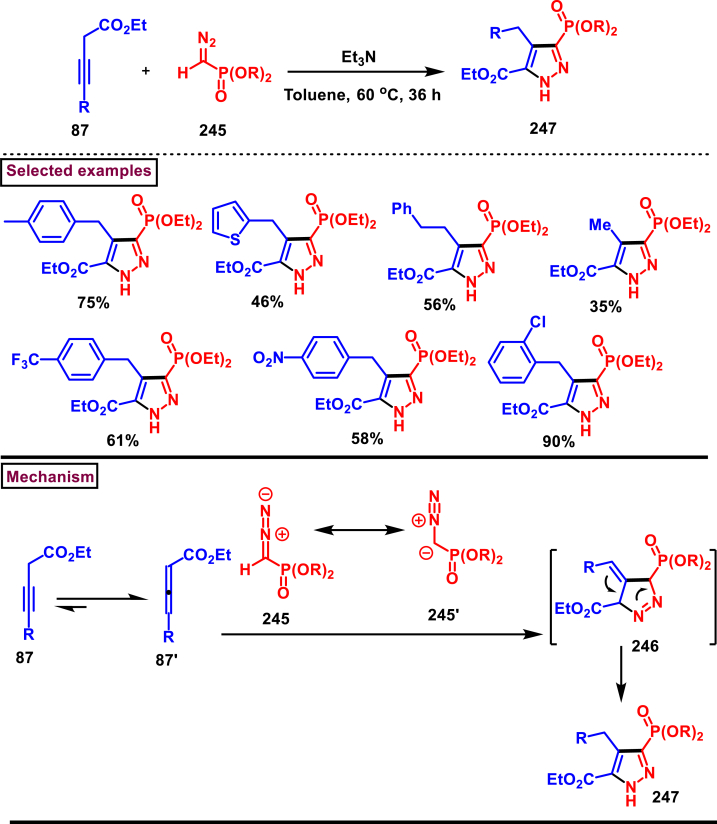
Synthesis of pyrazole-3-phosphonates *via* a regioselective [3 + 2] cycloaddition.

In 2021, Ma and Zhang et al. reported an interesting strategy to access a unique class of trisubstituted pyrazole-3-phosphonates (**247**) from a 3-alkynoates (**87**) and Seyferth-Gilbert reagent (**245**) *via* a regioselective [3 + 2] cycloaddition (Scheme 43) [82]. This transformation upon combining with alkylation sequence in a one-pot fashion afforded the fully substituted pyrazoles. This base-promoted method is a first example of cycloaddition where allenes are *in situ* generated from diazophosphonates (**245**) and served as a dipolarophile for the alkynoates **(87)** in this cycloaddition process. Broad scope was exerted with respect to 3-alkynoates (**87**). Electron-rich substituents in the aryl group resulted the desired products with modest yield, whereas, electron-withdrawing substituents gave the products much easily with less amount of Et_3_N due to the easy isomerization of alkynoates to allenoates. Interestingly, a complementary approach by directly coupling allenoates with Seyferth-Gilbert reagent (**245**) was also developed. Allenoate **(87’)** generated from the Et_3_N-assisted tautomerization of alkynoates **(87)** underwent cycloaddition with **(245)** to give the cyclized intermediate **(246)**. Isomerization of *via* a 1,3-*H* shift afforded the desired pyrazole **(247)**.

**Scheme 44:**
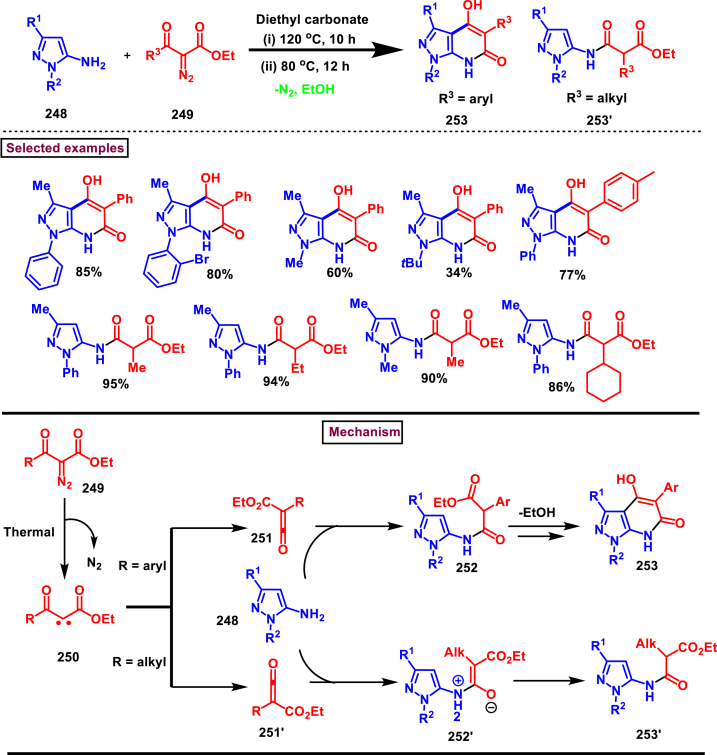
Synthesis of pyrazole derivatives *via* cascade Wolff rearrangement and acylation sequence.

A switchable access of 4-hydroxy-pyrazolo [3,4-*b*]pyridine-6-ones (**253**) and *N*-pyrazole amides (**253′**) were reported from 5-aminopyrazoles (**248**) and diazo precursors (**249**) *via* a cascade Wolff rearrangement and acylation sequence in a chemoselective manner (Zhu et al. 2021) (Scheme 44) [83]. This metal, additive and base free cascade method proceeded with the release of eco-friendly nitrogen. The reaction progress was affected by the sterically hindered substituents in both 5-aminopyrazoles (**248**) and diazo compounds (**249**). Other than this, reaction was tolerant to wide range of substituents on both the precursors. It was found that, α-diazo precursor bearing aryl ketone unit were converted selectively into pyrazolopyridines (**253**), whereas *N*-pyrazole amide (**253′**) was detected when alkyl ketone was present. This difference in chemoselectivity could be owing to the higher reactivity of intermediate in the cyclization step when aryl ketone is employed. Possible reaction pathway was proposed in which thermally generated carbene specie **(250)** was converted into ketene intermediates **(251** and **251′)**. Reaction of 5-aminopyrazole (**248**) with ketene intermediate **(251)** followed by cyclization and aromatization resulted in pyrazolopyridines (**253**). Alkyl ketene intermediate **(251′)** directly resulted in *N*-pyrazole amide (**253’**).

**Scheme 45:**
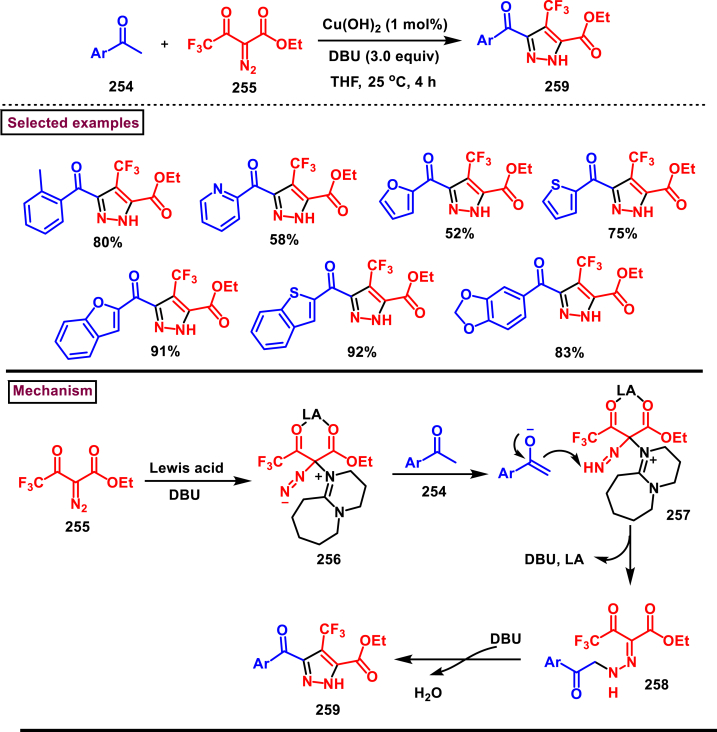
Synthesis of 4-CF_3_ pyrazoles *via* Lewis acid and base involving collaborative activation approach.

In 2020, Weng, Huang, and Xie et al. disclosed a Lewis acid and base involving collaborative activation approach for the synthesis of multi-substituted 4-CF_3_ pyrazoles (**259**) from diazo precursor **(255)** and aryl ketone **(254)** (Scheme 45) [84]. Mechanistically, coordination of Lewis acid and DBU with the diazoester (**255**) has made the terminal nitrogen more electrophilic **(256)** and facilitated the nucleophilic attack by ketone precursor (**254**) thus formation of C-N bond **(258)**. DBU-assisted intramolecular cyclization afforded the desired pyrazole **(259)**. Aryl, heteroaryl and styryl ketones (**254**) were resulted the desired pyrazole derivatives (**259**) in good yields, due to the lower reactivity alkyl ketones were not participated in this transformation. The synthetic utility was further demonstrated by various derivatizations such as, trans-amidation, N-methylation and hydrolysis of ester group. A series of control experiments were conducted to support the mechanism. Notably, a successful large-scale synthesis proved its synthetic potential.

**Scheme 46:**
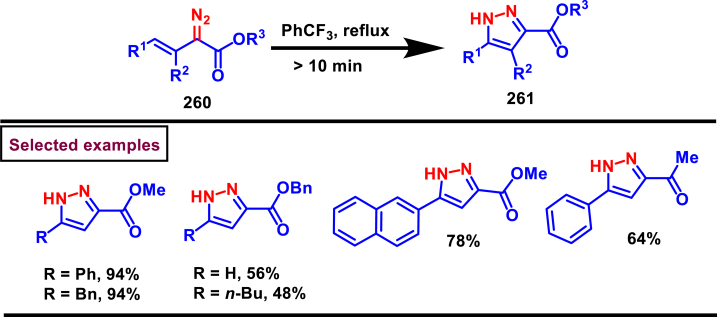
Synthesis of pyrazoles *via* thermal electrocyclization of vinyldiazoacetates.

A versatile route to synthesize multi-substituted pyrazoles (**261**) *via* thermal electrocyclization of vinyldiazoacetates (**260**) under catalyst and base free conditions was developed by Vilotijevic et al. (2020) (Scheme 46) [85]. Synthetic flexibility of this protocol allowed the access for mono, di- and tri-substituted pyrazoles (**261**) in higher yields and shorter reaction time. Vinyldiazoacetate precursors were obtained *via* literature routes. Azo compounds with differently substituted aryl, heteroaryl, alkyl units at C4 were tolerated and the corresponding products (**261**) were achieved in moderate to good yields. Notably, non-substituted diazo substrate was also equally participated in this transformation. Tri-substituted pyrazoles bearing alkyl and aryl groups were also readily prepared demonstrating the unique advantage of this method. Additionally, ester unit of **(260)** was successfully replaced with carboxylic acid and methyl ketone to afford the corresponding products. It was observed that, Z-vinyldiazoacetates and 4,4-disubstituted vinylacetates were not afforded the cyclized pyrazole products. Mechanistically, this transformation proceeded with thermal-electrocyclization followed by a 1,5-*H* shift.

**Scheme 47:**
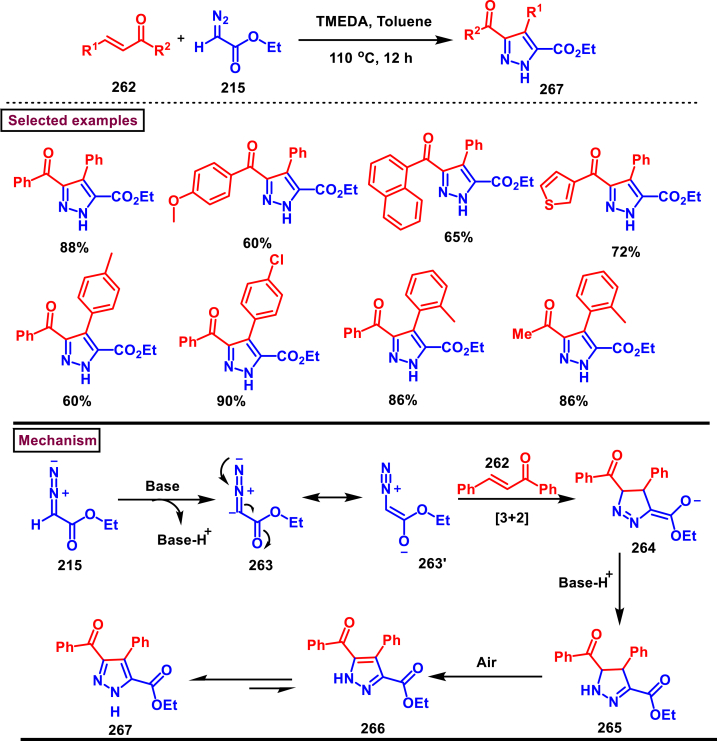
Synthesis of pyrazoles from enones and diazocarbonyl derivatives.

In 2020, Wang-coworkers reported a TMEDA promoted direct [3 + 2] cycloaddition of β-functionalized enones (**262**) and diazocarbonyl derivatives (**215**) to prepare pyrazoles (**267**) in good yields (Scheme 47) [86]. Wide range of α-diazo compounds (**215**) with alkyl, aryl, allylic esters reacted smoothly with chalcones and furnished the corresponding pyrazoles (**267**) in acceptable yields. On the other hand, β-functionalized enones (**262**) bearing aryl, naphthyl, thienyl, furanyl units were successfully converted into the desired products (**267**). Moreover, both alkyl and aryl ketone of the chalcone derivative conveniently yielded the desired pyrazoles. Cyclized intermediate **(264)** was obtained from the [3 + 2] cycloaddition of diazoester anion **(263** and **263’)** and chalcone **(262)**. Further protonation provided intermediate **(265)** which underwent aerial oxidation **(266)** and tautomerized into desired pyrazole **(267)**. Wide substrate scope, scalability and simple operational set-up are the key advantages of this report.

**Scheme 48:**
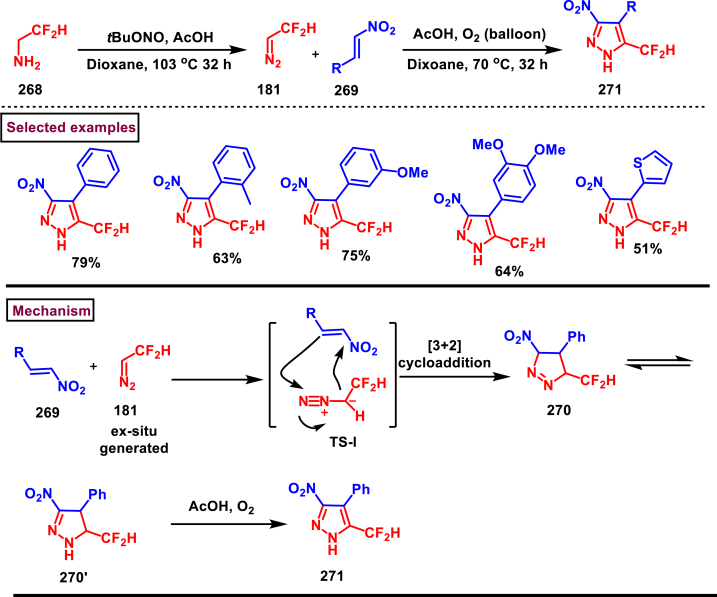
Regioselective synthesis of 5-CF_2_H-1*H*-pyrazoles from *ex situ* generated CF_2_HCHN_2._

In 2019, Chen and Han et al. reported a facile and regioselective synthesis of 5-difluoromethyl-3-nitro-1*H*-pyrazoles (**271**) from *ex situ* generated CF_2_HCHN_2_ (**181**) and nitroolefins (**269**) (Scheme 48) [87]. Generation of CF_2_HCHN_2_ was realized from CF_2_HCH_2_NH_2_ (**268**) and *t*BuONO in one chamber and introduced into other chamber containing nitroolefins in presence of O_2_/AcOH for subsequent [3 + 2] cycloaddition/aromatization. This two-chamber system was adopted to avoid the possible aza-Michael addition process. Aryl nitroolefins (**269**) with electronically, sterically distinct substituents, heteroaryl-nitroolefins, alkyl nitroolefins were participated well in this reaction, however, replacing nitro group into ester had a deteriorating effect. A gram scale-up experiment, derivatizations into useful products were demonstrated. In addition, synthesis of few pyrazole intermediates which are present in some biologically active compounds was also achieved. Reaction pathway proceeded through [3 + 2] cycloaddition of olefine precursor **(269)** and *ex situ* generated diazo compound **(181)** offered the intermediate **(270** and **270’)** *via* the **TS-I**. AcOH/O_2_-mediated oxidation furnished the desired pyrazole **(271)**.

**Scheme 49:**
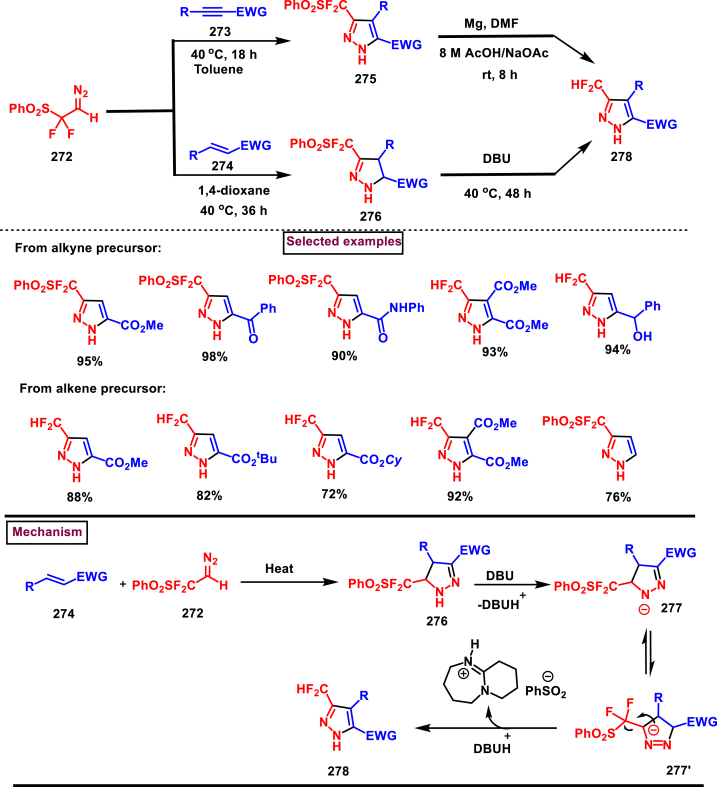
Regioselective synthesis of pyrazoles from phenylsulfone difluorodiazoethane *via* [3 + 2] cycloaddition.

In 2018, Ma and Zhang et al. demonstrated the potential utility of bench-stable phenylsulfone difluorodiazoethane (**272**) for the regioselective synthesis of difluoromethyl bearing pyrazoles (**278**) in high yields (Scheme 49) [88]. This unique reagent was served as masked difluorodiazoethane and prepared in five steps from thiophenol and ethyl bromodifluoroacetate. Electron deficient alkenes (**274**) and alkynes (**273**) were utilized as reacting partner in this [3 + 2] cycloaddition sequence. The synthetic utility of Ps-DFA was studied against various electron deficient alkynes precursors and the phenylsulfone difluoromethyl pyrazoles (**275** and **276**) were obtained in good yields. Smooth removal of phenylsulfone moiety was realized in the presence of magnesium at room temperature. Reaction pathway stated that, an isolable pyrazoline intermediate **(275** or **276)** was obtained *via* a [3 + 2] cycloaddition reaction between diazo compound **(272)** and alkene precursor **(273** or **274)**. DBU-assisted deprotonation, isomerization of anionic intermediate and elimination of PhSO_2_^-^ species delivered the pyrazole **(278)**.

**Scheme 50:**
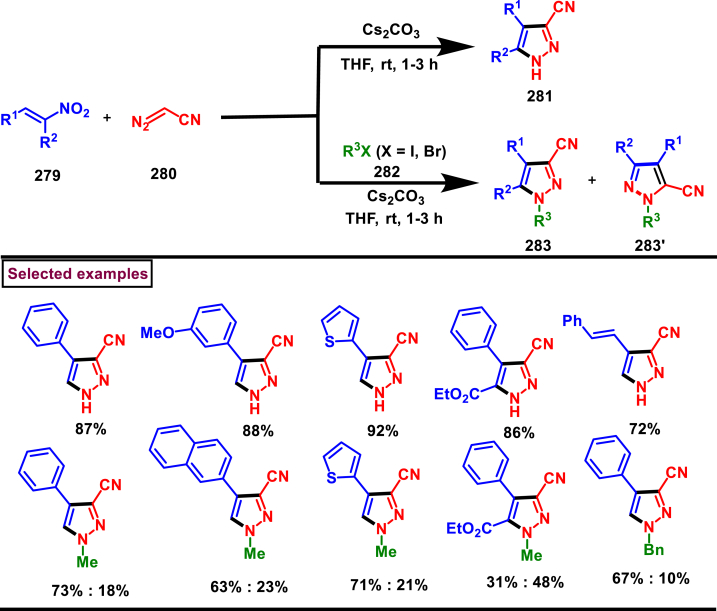
Synthesis of cyanopyrazoles from diazoacetonitrile and nitroolefines.

A base promoted [3 + 2] cycloaddition strategy for the preparation of diverse cyanopyrazoles (**281**, **283**–**283′**) from the reaction of diazoacetonitrile (**280**) and nitroolefines (**279**) was investigated by Ma et al. (Scheme 50) [89]. A series of 3-cyano pyrazoles (**281**) were obtained in high yields with notable regioselectivity. Additionaly, a three-component, one-pot method was also performed to prepare tetra substituted 3- and 5-cyanopyrazoles (**283** and **283’**) from nitro-olefines (**279**), alkyl halides (**282**) and diazoacetonitriles (**280**) in moderate regioselectivity. Irrespective of the substitution pattern and steric environment, various nitro-olefines were participated well in this transformation and the corresponding products were obtained in good yields. Nitrodienes and nitroeneynes were chemoselectively underwent cycloaddition at the α-position and provided the products with sensitive styryl and alkynic group. Wide substrate scope was excerted with respect to each reagent employed. The cyano group was converted into synthetically useful functionalities such as, aldehyde, amine and amide under various established conditions.

**Scheme 51:**
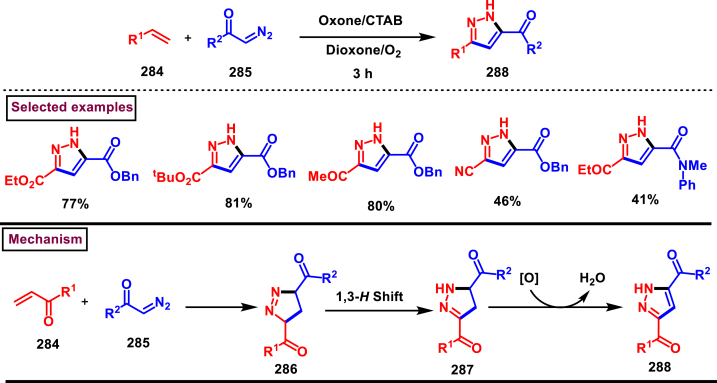
Oxone promoted synthesis of pyrazoles from α-diazoesters and olefines.

Oxidative cycloaddition of α-diazoesters (**285**) and electron deficient olefines (**284**) was promoted by oxone and 3,5-difunctionalized pyrazoles (**288**) were obtained (Vajjiravel et al. 2018) (Scheme 51) [90]. Wide range of alkenes (**284**) bearing cyano, ketone, esters, sulfones and tosyl groups were successfully involved in this transformation and the corresponding products (**288**) were obtained in moderate to good yields. In the case of acrylic acid precursor, decarboxylative cycloaddition afforded the monosubstituted pyrazoles. On the other hand, α-diazoesters with benzyloxy, ethoxy and amide linkages were smoothly engaged to afford the desired products (**288**) in good yields. Intermediate **(286)** was obtained *via* [3 + 2] cycloaddition of diazocarbonyl precursor **(285)** and alkene substrate **(284)**. Sequential *H*-shift and oxidation resulted the desired pyrazole **(288)**.

### 3.4 Synthesis of pyrazoles from nitrile imines

**Scheme 52:**
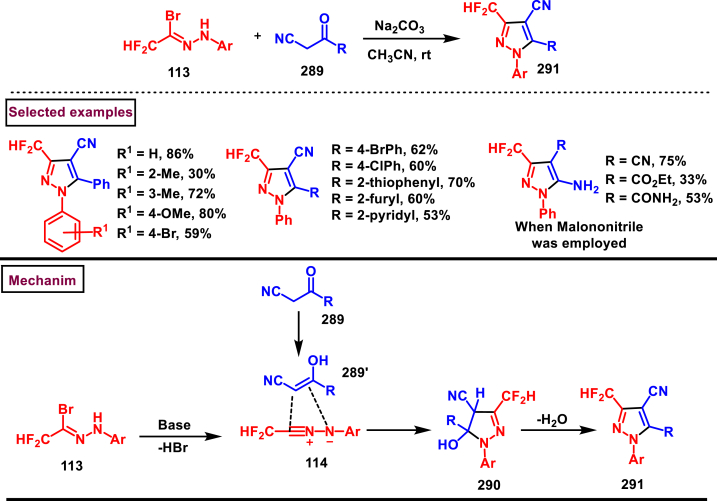
Synthesis of difluoromethylpyrazoles *via in situ* generated nitrilimines and nitriles.

Recently Hu and Huang et al. (2024) reported an efficient cyclization approach to prepare difluoromethylpyrazoles **(291)** from difluoro functionalized hydrazonoyl halides **(113)** and malononitrile or 2-acylacetonitrile **(289)** (Scheme 52) [91]. Various *N*-aryl-difluoroacetohydrazonoyl bromides **(113)** with electron rich aryl groups (-Me, -OMe, -^*t*^Bu) delivered the desired product in higher yields. In addition, sterically hindered aryl groups on **(113)** also impacted the yields and hydrazonoyl substrate with heteroaryl unit (2-Py) was failed to involve in this transformation. On the other end, 2-acylacetonitriles **(289)** bearing aryl and heteroaryl units were reacted well and the corresponding products were obtained in moderate yields, whereas, alkyl bearing 2-acylacetonitriles led to no desired products. Additionally, ethyl cyanoacetates malononitriles and cyanoacetamides were also screened, corresponding 2-aminopyrazoles were obtained. Mechanistically, enol form of 2- acylacetonitrile **(289’)** was reacted with *in situ* generated nitrile imine **(114)** and provided the cyclized intermediate **(290)**. Further dehydration furnished the desired pyrazole **(291)**. Overall, diverse range of fully substituted pyrazoles were obtained in good yields under mild conditions.

**Scheme 53:**
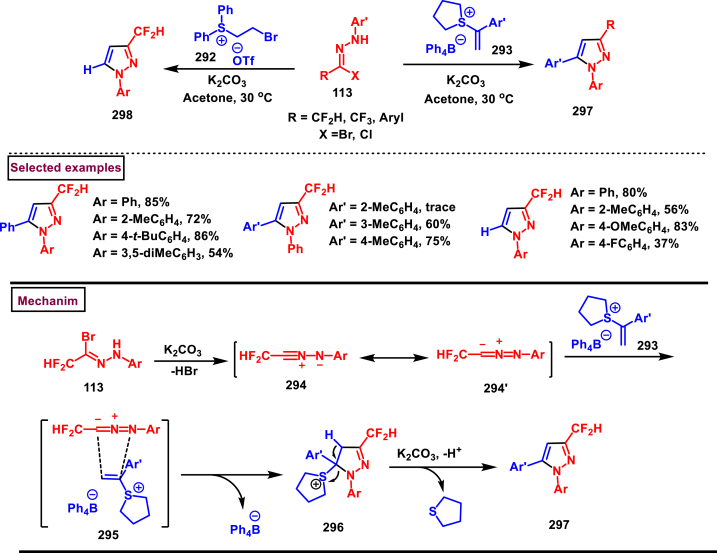
Synthesis of functionalized pyrazoles from *in situ* generated nitrilimines and vinyl sulfonium salts *via* a [3 + 2] cycloaddition.

A series of 5-aryl-3-difluoromethyl **(297)** and 5-unsubstituted-3-difluoromethyl pyrazoles **(298)** were prepared from the *in situ* generated nitrilimines and vinyl sulfonium salts **(293)** *via* a [3 + 2] cycloaddition Hu and chen et al. (2024), (Scheme 53) [92]. Both electronic and steric nature of the fluoroalkyl acetohydrazonoyl halide **(113)** were found crucial. Thus, electron rich aryl group in **(113)** led to higher yields and *ortho*-substitutions lowered the overall yields of the products **(297)**. On the other hand, electronic properties of aryl ring in vinylsulfonium salts **(293)** did not affect the yield and the desired products were obtained (35%–96 %) in excellent yields. Additionally, diphenyl (vinyl)sulfonium salts were generated *in situ* from (2-bromoethyl)diphenylsulfonium salts and efficiently annulated with nitrilimines **(294’)** and 5-unsubstituted-3-difluoromethyl pyrazoles **(298)** were obtained in (37%–83 %) yields. Base assisted generation of nitrilimines from the hydrazonoyl chloride **(113)** followed by a [3 + 2] cycloaddition with α-arylsulfonium salt **(293)** provided the pyrazoline intermediate **(296)**. Base mediated elimination of tetrahydrothiophene afforded the desired pyrazole **(297)**. Broad range of substrate scope, functional group tolerance and mild conditions are the appreciable features of this report.

**Scheme 54:**
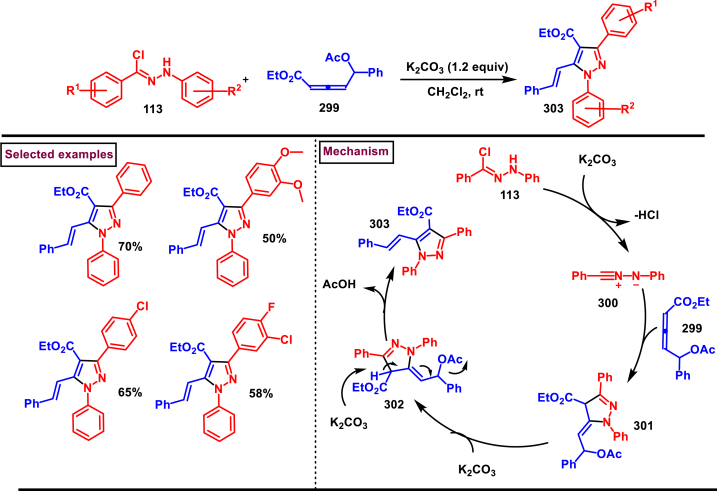
Synthesis of pyrazoles from *δ*-allenoates and nitrilimines *via* 1,3-dipolar cycloaddition.

Zhou et al. (2022) described a convenient method to prepare multi-substituted pyrazole derivatives (**303**) *via* a 1,3-dipolar cycloaddition of *δ*-allenoates (**299**) and *N*-phenyl-benzohydrazonoyl chloride (**113**) (Scheme 54) [93]. Nitrilimines bearing strong electron-withdrawing groups hampered the reaction and less yield was observed whereas, electron-donating groups, moderately electron-withdrawing substituents furnished the desired products (**303**) in good yields. In the case of *δ*-allenoates (**299**), acetate unit at C4 underwent elimination as AcOH and facilitated the cycloaddition. Synthetic utility was further demonstrated by converting the acetate group into a carboxylic acid, an alcohol and an amide under suitable conditions. Authors further evaluated the anticancer activity on lung cells (A549), human prostate (DU145) and carcinoma cells. Mechanistically, base assisted formation of nitrilimine **(300)** followed by a [3 + 2] cycloaddition with *δ*-allenoate generated intermediate **(299)** afforded the cyclic intermediate **(301)**. Sequential *H*-abstraction and elimination of AcOH generated the tetrasubstituted pyrazole **(303)**.

**Scheme 55:**
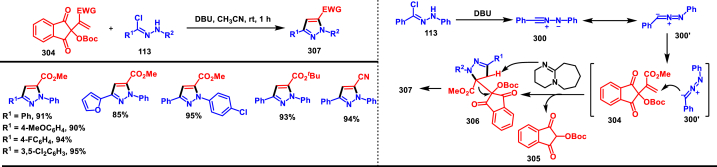
Synthesis of pyrazoles *via* [3 + 2] cycloaddition of carbonates and nitrilimines.

Another efficient method for the synthesis of 1,3,5-trifunctionalized pyrazoles (**307**) was developed by Chen et al. (2022) from ninhydrin-derived carbonates (**304**) and nitrilimines (**300′**) *via* [3 + 2] cycloaddition (Scheme 55) [94]. This 1,3-dipolar cycloaddition sequence offered wide range of pyrazoles (**307**) in high yielding with excellent regioselectivity. Alkyl, aryl, heteroaryl and poly-aryl bearing hydrazonyl chlorides **(113)** were smoothly reacted with ninhydrin-derived carbonate (**304**) and the desired pyrazoles (**307**) were obtained in excellent yields (85%–95 %). Especially, the substitution pattern and electronic nature of the substituents on the hydrazonyl chlorides hardly affected the outcome. Subsequently, the carbonate precursor bearing ester, ketone, cyano groups worked well to afford the pyrazoles (**307**) in good yields. Interestingly, tetrasubstituted pyrazoles were achieved by treating maleimide incorporated carbonates and spirocyclic pyrazolines were obtained with isatin derived carbonates. According to the proposed mechanism, DBU-mediated *in situ* generation of nitrilimine resulted in zwitter ionic forms **(300** and **300′)**. β-attack of **300’** on the MBH carbonate followed by elimination of dicarbonyl intermediate resulted in the formation of pyrazole **(307)**.

**Scheme 56:**
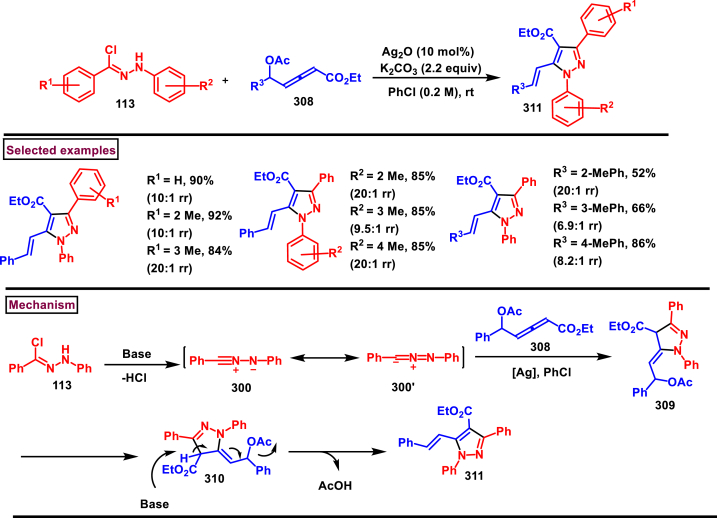
Ag-catalysed synthesis of pyrazoles from nitrilimines and allenoates.

K_2_CO_3_-mediated and Ag-catalysed synthesis of multi-substituted pyrazoles **(311)** was achieved *via* Huisgen cycloaddition of hydrazonyl chloride **(113)** and *δ*-allenoates **(308)** in a highly regioselective fashion (Li and Gao et al. 2022) (Scheme 56) [95]. Diverse range of pyrazoles including an intermediate of Lonazolac (anti-inflammatory drug) were accessed in good to excellent yields. According to the mechanistic proposal, base mediated initial formation of nitrile imine **(300** and **300’)** was realized which subsequently underwent [3 + 2] cycloaddition with *δ*-allenoates **(308)** to provide the intermediate **(309)**. Aromatization with the elimination of AcOH afforded the pyrazole **(311)**. Importantly, Ag-catalyst acted as a Lewis acid and coordinated both the substrates and facilitated the nucleophilic addition to the allene precursor **(308)**. Scope of the substrates were studies in detail and wide range of functionalized pyrazoles were accessed. In addition, growth-inhibition activity of Huh-7 cells were also evaluated for selected pyrazole derivatives.

**Scheme 57:**
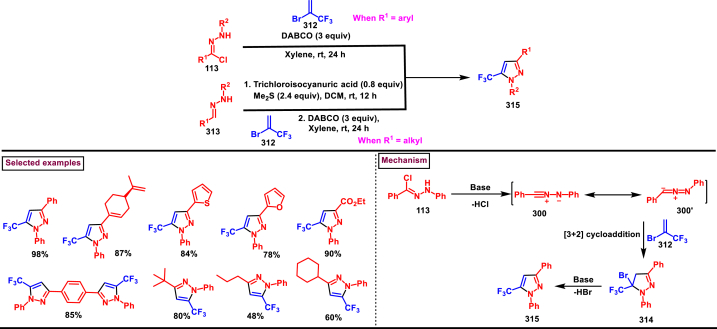
Base promoted synthesis of 5-CF_3_ pyrazoles *via* [3 + 2] cycloaddition.

In 2021, Zhu et al. reported a practical method to regioselectively synthesize 5-CF_3_ pyrazoles (**315**) from 2-bromo-3,3,3-trifluoropropene (**312**) and hydrazonyl chlorides (**113**) *via* [3 + 2] cycloaddition (Scheme 57) [96]. Base promoted dehydrochlorination of hydrazonyl chlorides generated nitrile imines **(300** and **300′)** which served as C-N-N synthons for the [3 + 2] cycloaddition process. Regardless of the electronic nature of the substituents on the aryl ring, wide range of hydrazonyl chlorides (**113**) were found to be excellent substrates and delivered the corresponding pyrazoles **(315)** in good yields. Owing to the unstable nature of alkyl hydrazonoyl chlorides, a two-step, one pot method was developed by reacting directly hydrazones (**313**) and the alkylated products (**315**) were isolated successfully in high yields. Application of this method has been extended by preparing a key intermediate of sphingosine-1-phosphate receptor. Base-assisted dehydrochlorination of **(113)** generated nitrilimines **(300** and **300’)** as a zwitter ionic mixture. Further regioselective [3 + 2] cycloaddition with 2-bromo-3,3,3-trifluoropropene **(312)** delivered the pyrazoline intermediate **(314)** which underwent aromatization *via* base promoted dehydrobromination to furnish the pyrazoles **(315)**. Operational simplicity, scalability, environmentally-friendly feedstock (BTP), wide substrate scope are the merits of this report.

**Scheme 58:**
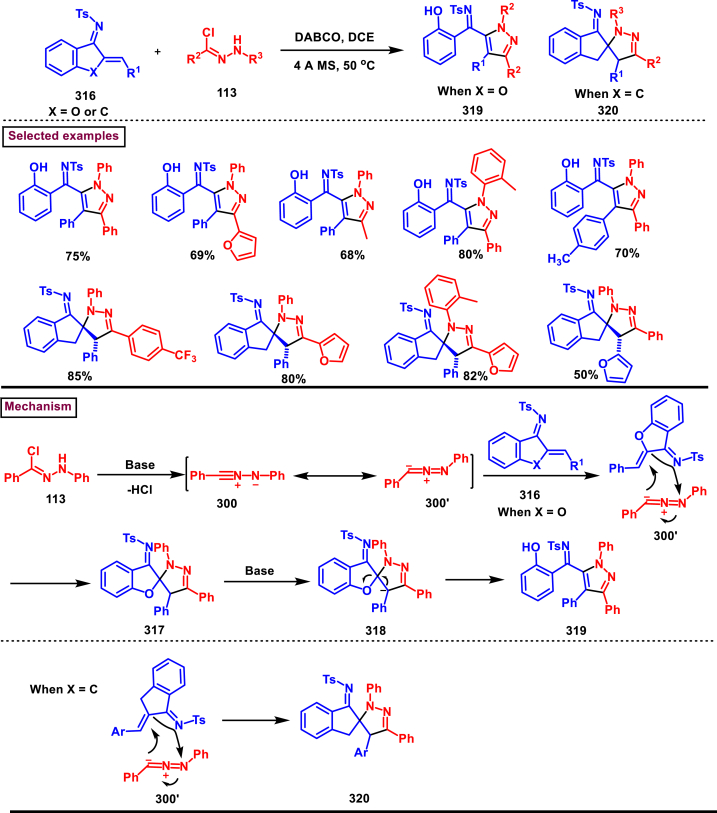
Tandem synthesis of pyrazoles *via* [3 + 2] cycloaddition/ring opening rearrangement.

In 2022, Liu and Zheng et al. reported a new method for the preparation of highly functionalized pyrazoles (**319** and **320**) from the benzofuran derived azadienes (BDAs) (**316**) and nitrile imines (**300′**) *via* [3 + 2] cycloaddition/ring opening rearrangement reaction in a tandem fashion (Scheme 58) [97]. A minor modification in the substrate also allowed to access spiro-pyrazolines (**320**). A detailed optimization studies were carried out to choose the best conditions. Solvent like MeOH underwent Michael addition with the benzofuran derived azadiene. The reaction was found to be general with various substituted *N*-phenylbenzohydrazonoyl chlorides (**113**) bearing electron donating and withdrawing groups. It was also observed that sterically hindrance is crucial and resulted in mixture of products. Similarly, a series of BDAs (**316**) with electronically and sterically distinct functional groups were yielded the corresponding products in good yields. When azadienes obtained from 2,3-dihydro-1*H*-inden-1-one were reacted under this conditions, spiro-pyrazolines (**320**) were resulted in good yields. To showcase the synthetic utility a gram scale synthesis and late-stage modifications of the product were successfully done. A possible mechanistic rational was also proposed in which base mediated dehydrochlorination of *N*-phenylbenzohydrazonoyl chloride (**113**) resulted in the nitrile imine (**300** and **300’** zwitter-ionic forms) which reacted with azadiene **(316)** to yield the spiro intermediate **(317)**. Further ring-opening rearrangement furnished the desired pyrazoles (**319**).

**Scheme 59:**
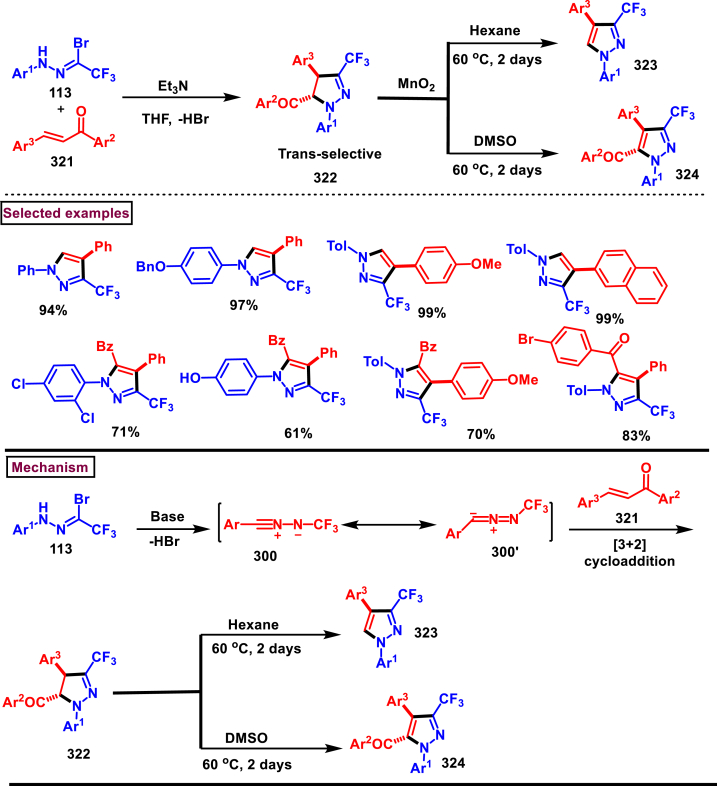
Synthesis of 3-CF_3_ pyrazoles *via* [3 + 2] cycloaddition of enones.

In 2022, Jasinski and co-workers reported a novel procedure for the synthesis of two types of multi-functionalized 3-CF_3_ pyrazoles (**323** and **324**) from 5-acyl-pyrazolines (**322**) as a common precursor (Scheme 59) [98]. Trans isomer of pyrazolines **(322)** were obtained in a regio and diastereoselective manner *via* a [3 + 2] cycloaddition of enones **(321)** with *in situ* prepared trifluoroacetonitrile imines (**300** and **300’**). During the MnO_2_-based oxidation step, either fully substituted pyrazoles (**324**, oxidative aromatization in DMSO) or 1,3,4-trisubstituted pyrazoles (**323**, deacylative pathway in hexane) were obtained depending on the solvent (hexane or DMSO). The reported procedure was successfully scaled-up and exhibited wide tolerance to varieties of substituents and functional groups.

**Scheme 60:**
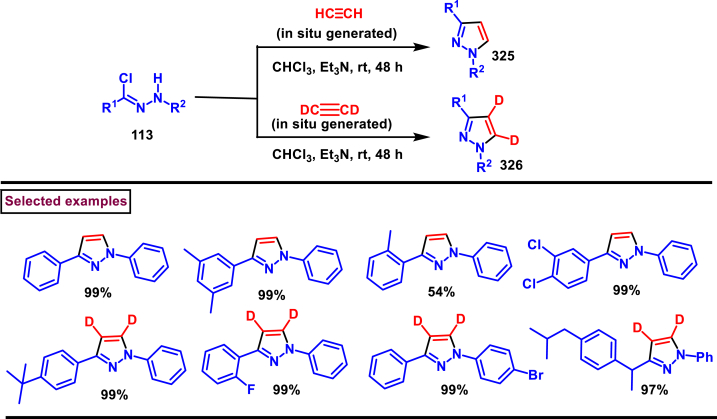
Novel synthesis of 1,3-disubstituted pyrazoles in two-chamber reactor system.

In 2018, Ananikov et al. reported a novel methodology for the synthesis of 1,3-disubstituted pyrazoles (**325**) in two-chamber reactor system through *in situ* prepared nitrile imines and acetylenes (Scheme 60) [99]. Nitrile imine precursor hydrazonoyl chloride (**113**) and a base was loaded into a chamber whereas, the acetylene was produced from calcium carbide and water in another chamber. This spatial partioning was opted to separate the water-reactive nitrile imines from the acetylene-preparing chamber. External supply of acetylene gas led to higher consumption due to the loss in the system. Wide range of *N′*-arylbenzohydazonoyl chlorides (**113**) with differently substituted aryl units were tested and the desired pyrazoles (**325**) were obtained in good yields. In addition, 4,5-dideuteropyrazoles (**326**) were also obtained by generating deuterated acetylene in presence of calcium carbide and D_2_O. To gain mechanistic insights, DFT studies were performed.

### 3.5 Synthesis of pyrazoles from aryldiazonium salts

**Scheme 61:**
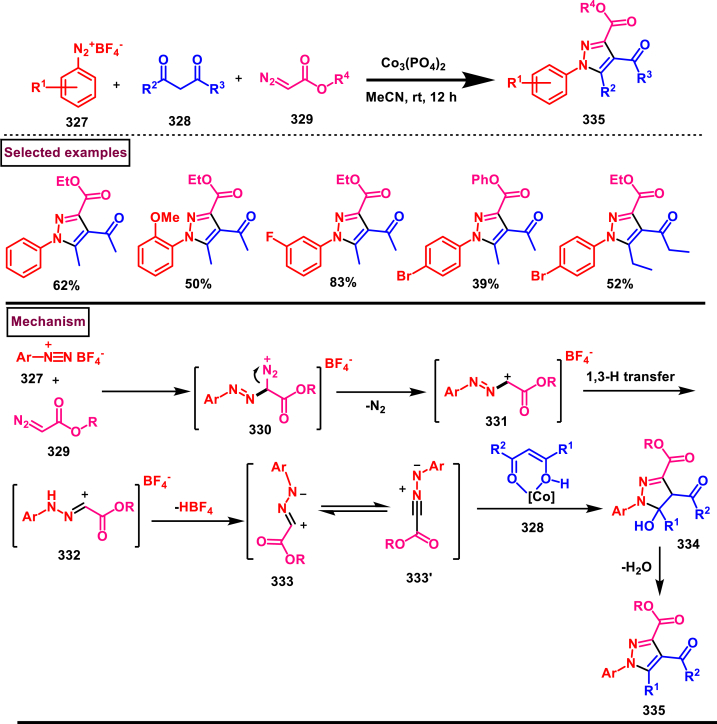
Co-catalysed three-component synthesis of fully substituted pyrazoles.

Wan and Shao et al. reported a cobalt-catalysed three component approach for the synthesis of fully substituted pyrazoles (**335**) from aryldiazonium salts (**327**), diazo esters (**329**) and 1,3-dicarbonyl compounds (**328**) (Scheme 61) [100]. Mechanistically, *in situ* generation of nitrile imines **(333** and **333’)** from diazo esters (**329**) and aryldiazonium salts **(327)** and further cycloaddition with dicarbonyl compounds (**327**) resulted the pyrazoline intermediate **(334)**. Elimination of H_2_O gave the aromatized pyrazole **(335)**. Wide substrate scope, mild conditions, readily available precursors are the notable features of this report. In addition, this method offered a convenient alternate route to generate the nitrile imines from aryldiazonium salts (**327**) which is usually accessed *via* challenging dehydro-halogenation of hydrazonoyl halides. Aryldiazonium salts bearing electronically distinct groups at various positions of aromatic ring well participated in this reaction and the corresponding pyrazoles (**335**) are obtained in good yields. Similarly, various diazo esters and 1,3-dicarbonyl compounds were also equally effective for this transformation. A series of control experiments were conducted to support the proposed mechanism.

**Scheme 62:**
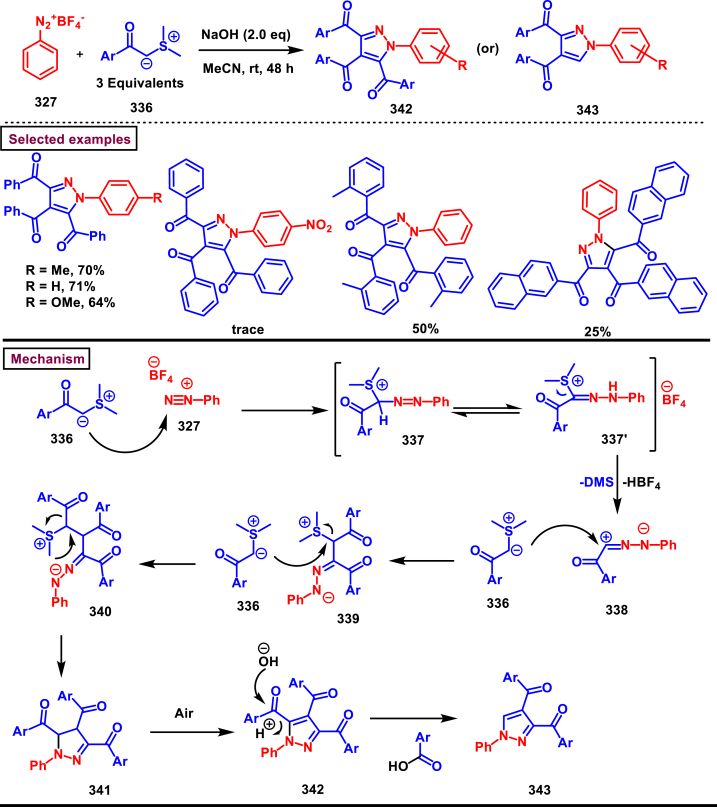
Domino synthesis of multisubstituted pyrazoles from aryldiazonium salts.

A novel domino method was described by Shao et al. (2021) to prepare tri- and tetra-substituted pyrazoles (**342** and **343**) from aryldiazonium salts (**327**) and sulfur ylides (**336**) (Scheme 62) [101]. This annulation strategy utilized 3 mol of sulfur ylides (**336**) and five new bonds were created in a one-pot operation. A detailed investigation to find the optimal conditions was carried out. It was found that tetra-substituted pyrazoles (**342**) were obtained with 2 equivalents of base (NaOH) whereas, trisubstituted pyrazoles (**343**) required more amount of base. Among various aryldiazonium salts (**327**) screened, strong electron-withdrawing substituents resulted in lower yields. On the other hand, sulfur ylides (**336**) bearing electron-donating group were found to be unfavourable for this transformation. Synthetically useful late-stage modification was performed to prepare pyrazolo[3,4-*d*]pyridazine. This (2 + 1+1 + 1) annulation process was initiated by the nucleophilic attack of nitrile imine **(337** and **337’)** on aryldiazonium species **(327).** Repeated SN_2_ process led to dihydropyrazole intermediate **(341)**. Aireal oxidation resulted in **(342)** whereas, tri-substituted **(343)** was obtained in the presence of NaOH.

**Scheme 63:**
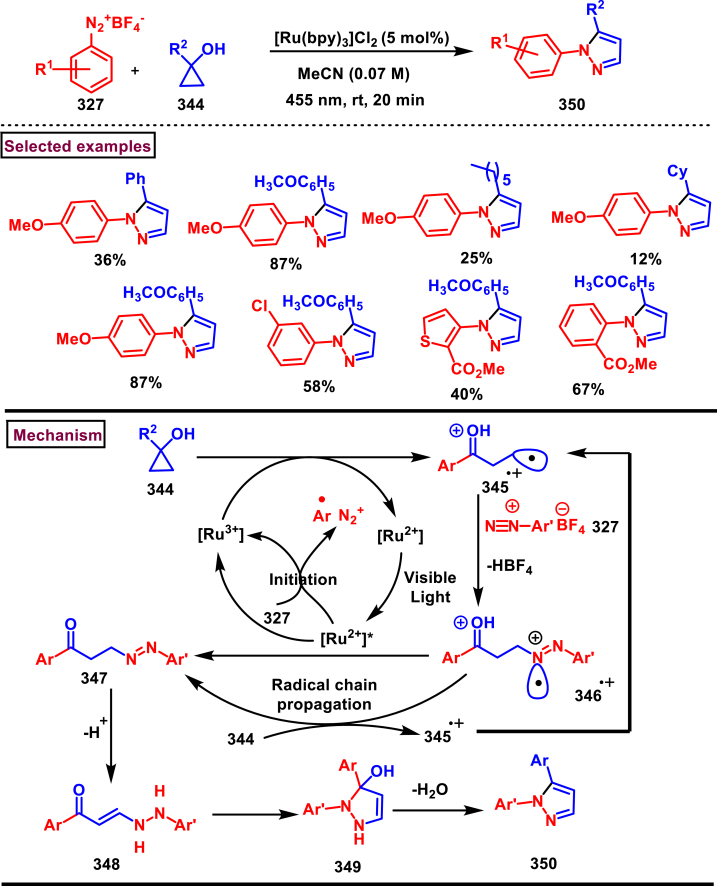
Ru-catalysed photocatalytic synthesis of 1,5-diaryl pyrazoles.

A photocatalytic, regiocontrolled preparation of 1,5-diaryl pyrazoles (**350**) from aryldiazonium salts (**327**) and aryl cyclopropanols (**344**) was reported by Wangelin and Majek et al. (2020) (Scheme 63)[102]. By using catalytic [Ru(bpy)_3_]Cl_2_ under blue-light varieties of pyrazoles (**350**) were obtained under mild conditions. Excellent regiocontrol, functional group compatibility, detailed mechanistic studies are the key advantages of this method. In the case of aryldiazonium salts (**327**), excellent substrate scope was observed irrespective of electronic properties. However, in the case of cyclopropanol (**344**) electron-donating groups shown good conversion. A radical chain pathway was supported by a series of theoretical, synthetic and spectroscopical studies. Mechanistically, excited photocatalyst oxidized the cyclopropanol (**344**) resulted in ring opening **(345**^**.+**^**)**. Further trapping of aryldiazonium salt generated the radical cation **(346)** which was converted into stable diazo ketone **(347)**. Rearrangement into a diazo-enone **(348)** followed by an intramolecular cyclization resulted in **(349)** and final dehydration yielded the 1,5-diaryl pyrazoles **(350)** regioselectively.

**Scheme 64:**
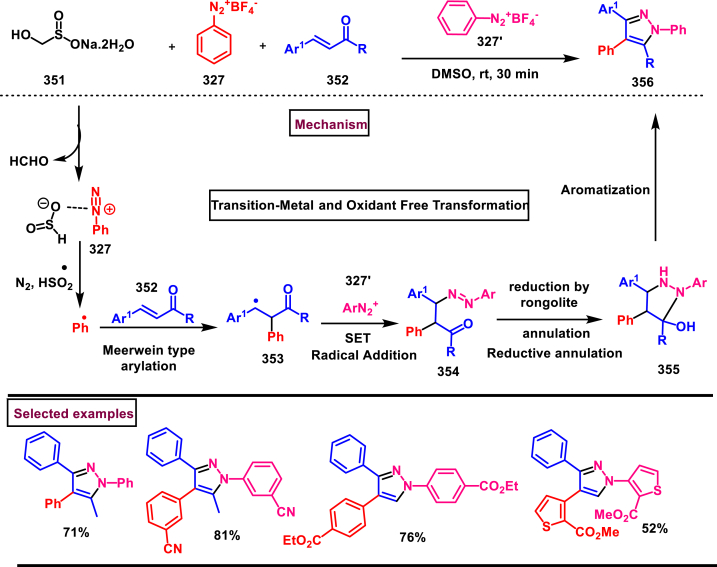
Rongalite mediated synthesis of pyrazoles from α,β-unsaturated carbonyl compounds and aryldiazonium salts *via* radical annulation.

A straightforward approach to prepare multi-substituted pyrazole derivatives (**356**) was realized by Wu et al. in 2019 (Scheme 64) [103]. This method utilized α,β-unsaturated carbonyl compounds (**352**) and aryldiazonium salts (**327** and **327′**) in the presence of rongalite **(351)** and proceeded *via* radical annulation. Rongalite served as radical initiator as well as reducing agent under this conditions. Notably, this three component synthesis furnished synthetically useful pyrazole derivatives (**356**) under metal and oxidant free conditions at room temperature making this method green and practicable. A systematic study for the scope of aryldiazonium salts (**327** and **327′**) was carried out with electronically neutral, electron-withdrawing and relasing substituents, and the desired products were obtained in good yields. On the other hand, α,β-unsaturated aldehydes and ketones (**352**) were also well-participated in this reaction. Involvement of radical species was proved by radical trapping reagent (TEMPO) and the plausible mechanism was also reported. Accordingly, Meerwein-type arylation of *in situ* generated phenyl radical into α,β-unsaturated carbonyl compound **(352)** was identified as key step. Addition of another molecule of diazonium salt **(327’)** to this radical, diazo reduction, cyclization **(355)** and aromatization resulted the fully substituted pyrazoles (**356**).

### 3.6 Synthesis of pyrazoles from 1,3-dicarbonyl compounds

**Scheme 65:**
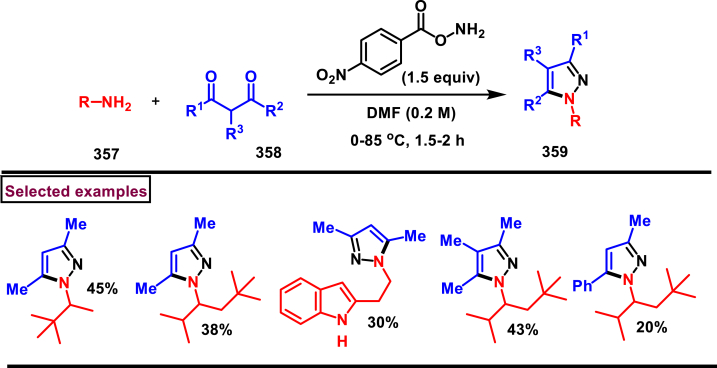
Synthesis of *N*-alkyl and *N*-aryl pyrazoles from 1,3-diketones and amines.

A novel method for the synthesis of *N*-alkyl and *N*-aryl pyrazoles **(359)** from 1,3-diketones **(358)** and aromatic or primary aliphatic amines **(357)** as an aminating agent was reported by Gulia et al. (Scheme 65) [104]. Aliphatic amines with sterically hindered substitutions gave moderate yields whereas, aryl amines gave the desired products **(359)** in comparatively higher yields (47%–70 %). Arylamines with sensitive functional groups suchas, halogens, esters, were well tollerated. However, phenolic amines were led to complex mixtures owing to the competitive O-amination. On the other hand, steric nature of diketones was crucial as, di-^*t*^Bu-1,3-diketones and aryl-alkyl 1,3-diketones were found less reactive and the desired products **(359)** were obtained in lower yields. Despite the lower yields, transition metal-free conditions, simple procedures, readily available substrates are the appreciable features of this method.

**Scheme 66:**
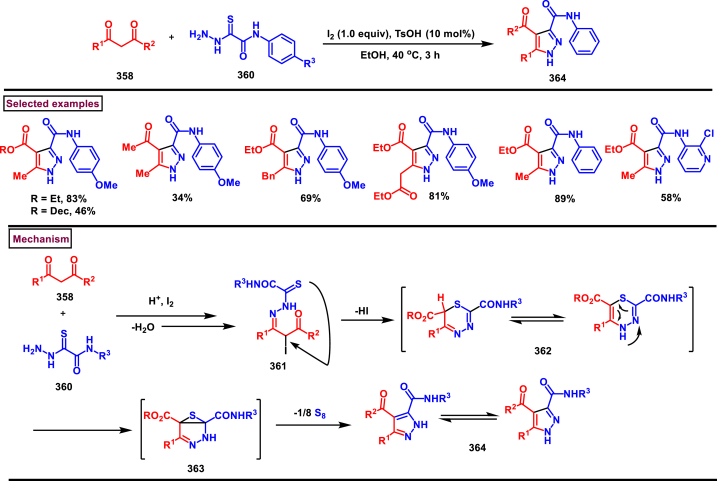
Synthesis of 3,4-dicarbonyl functionalized pyrazoles from oxamic acid thiohydrazides with 1,3-dicarbonyl compounds.

Volkova et al. reported a I_2_-mediated conveninet strategy to prepare 3,4-dicarbonyl functionalized pyrazoles **(364)** from direct reaction of oxamic acid thiohydrazides **(360)** with 1,3-dicarbonyl compounds **(358)** (Scheme 66) [105]. This process proceeded through a series of condensation/halogenation/intramolecular cyclization and ring contraction with the elimination of elemental sulfur. Excellent substrate scope was exhibited by various 1,3-dicarbonyl compounds such as, keto esters, acetoacetic esters and 1,3-diketones. Importantly testosterone derived steroidal derivative was obtained in 81 % yield. 1,3-diaryl ketone exerted poor reactivity and no desired product was obtained. oxamic acid thiohydrazides **(360)** also shown excellent substrate scope and diverse range of products were obained in good yields. Initial condensation of 1,3-dicarbonyl derivative **(358)** with oxamic acid thiohydrazide **(360)** provided the hydrazone which rapidly underwent halogenation **(361)**. Intramolecular displacement of halogen by S-nucelophile gave the intermediate **(362)** which after elimination of elemental sulfur afforded the desired product **(364)**. Gram scale synthesis, post synthetic transformation and control experiemnts to obtain mechanistic insights were the notable features of this report.

**Scheme 67:**
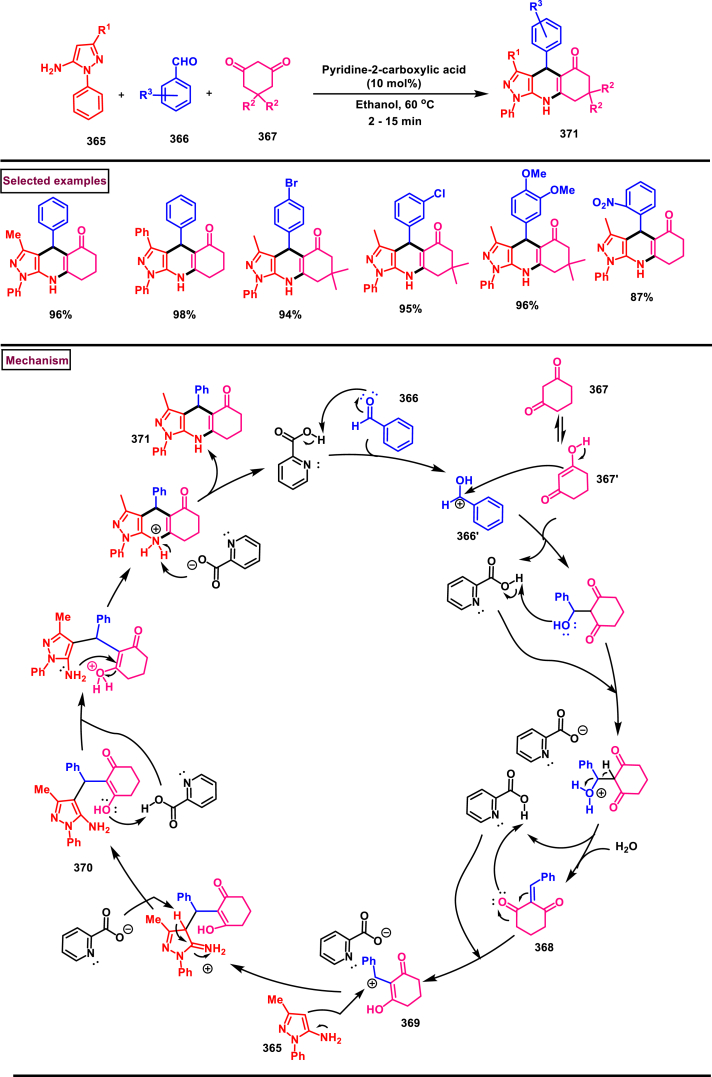
PC2A catalysed regioselective synthesis of pyrazolo[3,4-*b*]quinolinones.

A sustainable and regioselective synthesis of pyrazolo[3,4-*b*]quinolinones **(371)** was achieved from 1,3-diketones **(367)**, *N*-aryl-5-amino-pyrazoles **(365)** and aryl aldehyde precursors **(366)** by employing pyridine-2-carboxylic acid as a catalyst (Patel et al. 2020). (Scheme 67) [106]. This multicomponent approach proceeded through a Knoevenagel condensation, intermolecular C-alkylation, cyclization and dehydration. Limitations were studied with various aldehydes with electron donating and withdrawing groups including free -OH and halogens and the corresponding products were isolated in excellent yields (84%–99 %). Notably, reaction completed fastly (2 min) when electro donating groups (-OMe) were present on the aryl ring of aldehyde whereas, electron withdrawing groups (-NO_2_) led to completion in 10 min. Readily available 1,3-cyclic diketones **(367)** and pyrazole amines **(365)** were studied to afford the pyrazolo[3,4-*b*]quinolinones **(371)** in excellent yields. Pyridine-2-carboxylic acid played a crucial dual basic and acidic nature in this process. Activated aldehyde **(366′)** underwent Knoevenagel condensation with diketone **(367’)** to afford alkene **(368)** which was convereted into cation **(369)** in the presence of the PCA catalyst. 2-amino pyrazole nucleophilically reacted with the cation intermediate to give the C-alkylated product **(370)** which upon intramolecular cyclization and dehydration yileded the desired pyrazolo[3,4-*b*]quinolinone **(371)**.

**Scheme 68:**
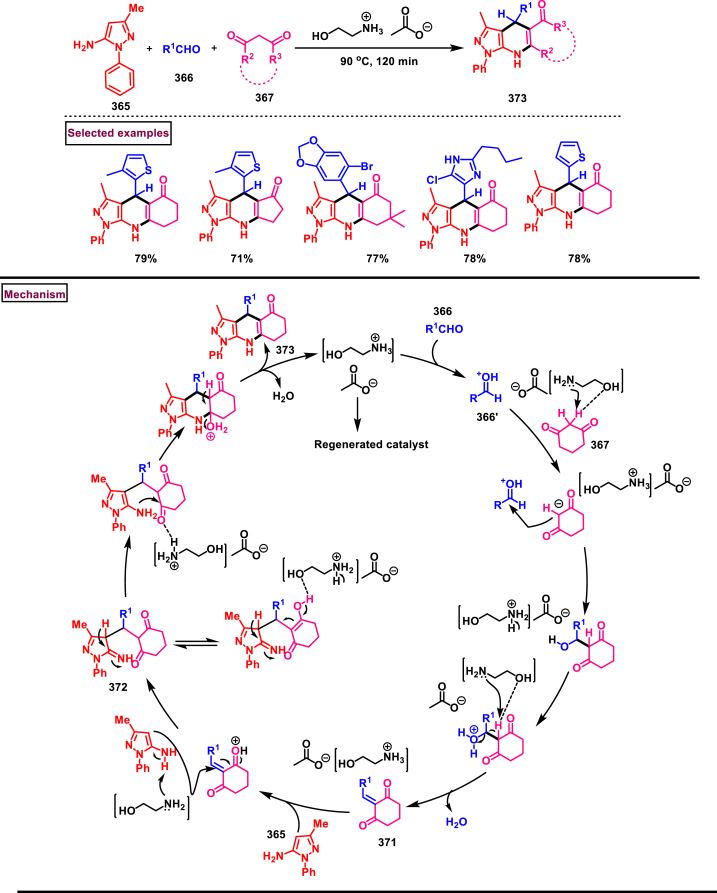
Ionic liquid mediated multi-component synthesis of pyrazolo dihydropyridine.

In 2019, Patel et al. disclosed a multicomponent approach to prepare pyrazolo dihydropyridine derivatives **(373)** from *N*-arylated-1*H*-pyrazole-5-amine **(365)**, 1,3-cyclic ketones **(367)** and aldehyde precursors **(366)** under the influence of ionic liquid (HEAA-II) (Scheme 68) [107]. This method enabled the formation of one C-N bond and two C-C bonds in a one-pot operation and proceeded through Knoevenagel condensation/intermolecular aza-Michael addition and intramolecular cyclization. A series of pyrazolo dihydropyridines **(373)** were obtained under environmentally friendly conditions with H_2_O as a byproduct. Detailed mechanistic elucidation was proposed by highlighting the crucial roles of reaction media (ionic liquid-HEAA-II). Hydroxy ethyl ammonium acetate was used as ionic liquid which acted as proton donor and subsequently protonated the aldehyde precursor **(366’)**. Basic hydroxy alkyl amine was liberated which generated the enolate of 1,3-dicarbonyl substrate **(367)**. Alkene **(371)** was generated *via* condensation which was transformed into cation through dehydration. Coupling with amine **(365)**, followed by intramolecular cyclization and dehydration provided the desired product **(373)**. Ionic liquid reusability, gram scale preparation, mild conditions are the attractive features of this approach. Notably, selected pyrazolo dihydropyridines were evaluated for their *in vitro* antibacterial, antitubercular, antifungal and anti-proliferative activities.

**Scheme 69:**
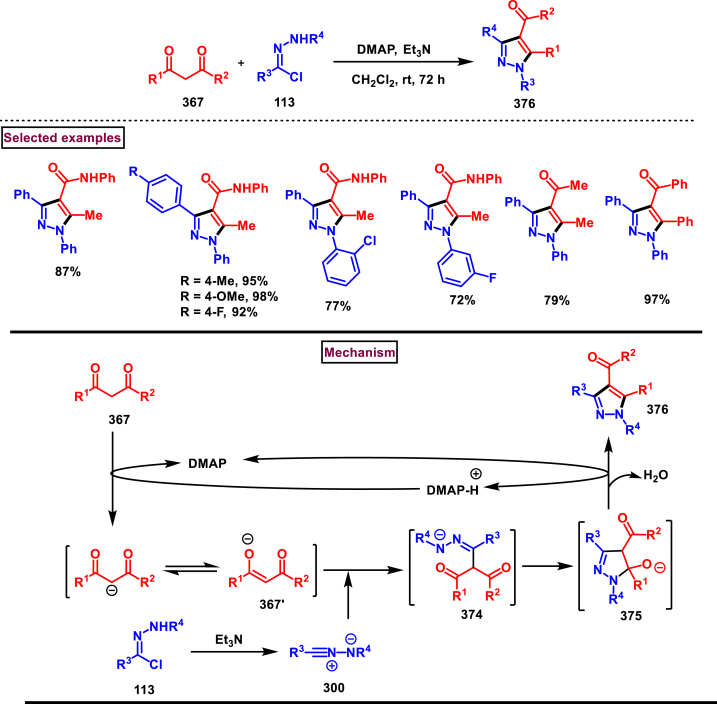
Synthesis of pyrazoles *via* a regioselective [3 + 2] cycloaddition of hydrazonoyl chloride and 1,3-dicarbonyl compounds.

Broad range of poly substituted pyrazoles **(376)** were prepared *via* a regioselective [3 + 2] cycloaddition of *in situ* generated nitrilimines **(300)** and 1,3-dicarbonyl compounds **(367)** in the presence of DMAP and triethylamine (Li et al. 2018) (Scheme 69) [108]. Wide range of C and N-aryl substituted hydrazonyl chlorides **(113)** were studied under these conditions. Irrespective of the steric nature, substitution pattern, and electronic property of the substituents on the hydrazonoyl chloride, corresponding products **(376)** were obtained in good yields. Notably, C-aryl and *N*-alkyl substitution led to the pyrazoles in moderate yield whereas, C-alkyl and N-aryl substitution on the hydrazonyl chloride led to no product. On the other hand, various 3-oxo-*N*-arylbutanamides with different substitutions on aryl ring were studied and the corresponding products **(376)** were obtained in excellent yields (74%–92 %). 1,3-diaryl and 1,3-dialkyl ketones were successfully screened. However, 1,3-keto ester failed to give the desired product. Base assisted *in situ* generated nitrilimine **(300)** reacted with enolic form of 1,3-dicarbonyl substrate **(367’)** and resulted in intermediate **(374)**. Further intramolecular cyclization and dehydration gave the desired product **(376)**.

### 3.7 Synthesis of pyrazolines and pyrazolones

**Scheme 70:**
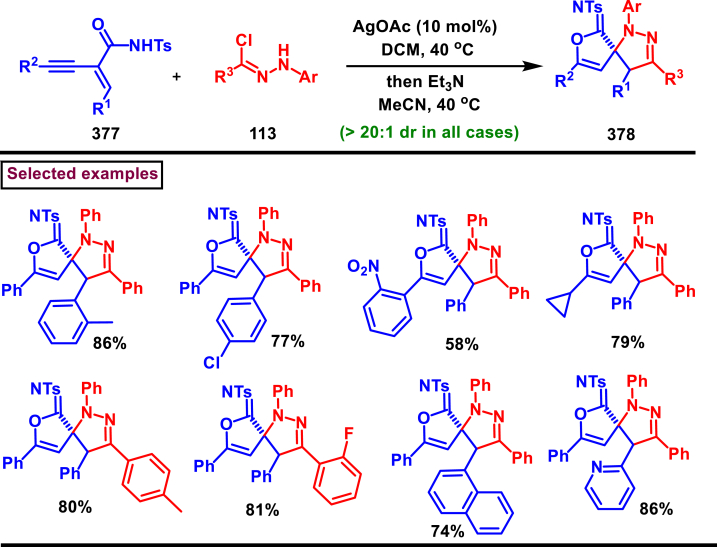
One-pot Ag-catalysed synthesis of pyrazole cascade cyclo-isomerization/[2 + 3] cycloaddition.

In 2013, an elegant approach to prepare a series of spiropyrazolines (**378**) *via* a one-pot silver catalysed cascade cycloisomerization/[2 + 3] cycloaddition of *in situ* generated nitrile imines with enynamides **(377)** was reported by Zhan et al. (Scheme 70) [109]. This transformation was proceeded under mild conditions and the desired products were obtained in good yields. *In vitro* biological studies showed that, spiropyrazolines **(378)** with cyclopropyl unit exhibited excellent anti-proliferative effect on several cancer lines. Under the optimized conditions, the reaction scope was explored in detail. Various aryl groups at alkene unit of ynamides (**377**) furnished the corresponding products **(378)** in good yields and diastereoselectivity. Similarly, aryl and alkyl groups connected at alkyne unit were also resulted the desired spiropyrazolines (**378**) in acceptable yields. Subsequently, differently substituted hydrazonoyl chlorides (**113**) were also exhibited good scope under these conditions. A gram scale synthesis, late-stage chemical transformation, biological studies are the notable features of this report.

**Scheme 71:**
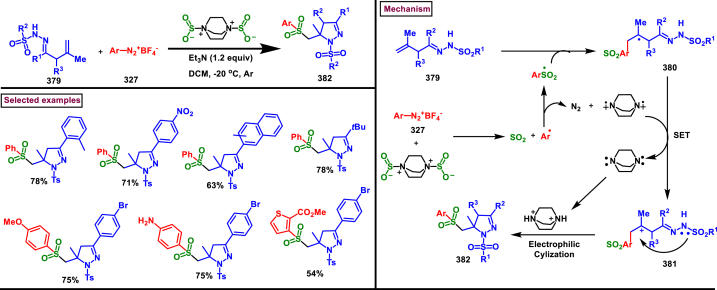
DABSO mediated synthesis of pyrazoles *via* direct arylsulfonylation.

A radical initiated approach to access sulfonated pyrazolines (**382**) from β,γ-unsaturated hydrazones (**379**), aryldiazonium salts (**327**), and DABSO *via* direct arylsulfonylation (Jiang et al. 2023) (Scheme 71) [110]. Arylsulfonyl radicals was generated *in situ* from the diazonium salts (**327**) and DABSO which triggered this transformation. Moreover, DABSO served as sulfone source as well as an oxidant in this cascade reaction. Various reaction condition such as, temperature, solvents, base were screened. In the case of β,γ-unsaturated hydrazones (**379**), aryl/heteroaryl unit with different electronic properties and substitution patterns were found to be compatible an yielded the expected pyrazolines (**382**). Both aryl and alkyl sulfonyl hydrazines were easily converted into desired pyrazoline derivatives. On the other hand, aryldiazonium salts (**327**) with electron-donating group furnished the products in good yields compared to electron-withdrawing substituents. Gratifyingly, it was observed that the *γ*,*δ*-unsaturated hydrazone was also participated in this multi-component cascade cyclization. Radical pathway was undoubtably confirmed by using various radical trapping agents. Mechanistic insights proposed that, the formation of key arylsulfonyl radical was generated by the interaction of DABSO and aryldiazonium salt (**327**) *via* a SET process and a cleavage of N-S bond. Addition to alkene unit of β,γ-unsaturated hydrazones (**379**), additional SET process and intramolecular electrophilic cyclization resulted the pyrazolines (**382**) with the formation of a new C-N and C-S bond in the absence of external catalyst or oxidant.

**Scheme 72:**
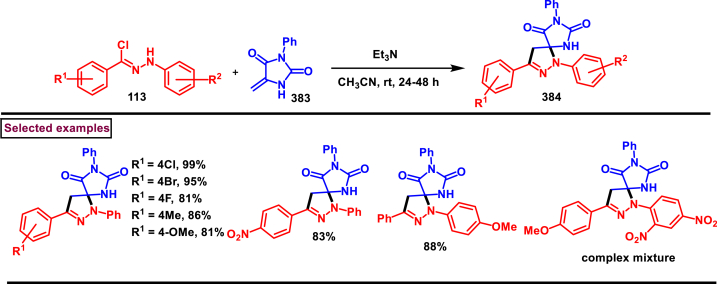
Regioselective synthesis of spiro-pyrazoline-imidazolidine-2,4-diones from *in situ* generated from nitrilimines and hydantoin derivatives.

Regioselective preparation of spiro-pyrazoline-imidazolidine-2,4-diones was achieved from *in situ* generated from nitrilimines and 5-methylidene-3-phenyl-hydantoin **(383)** (Beloglazkina et al. 2023) (Scheme 72) [111]. Secondary arylamine of nitrilimine regioselectively reacted at the sterically hindered carbon of hydantoin derivative. Electron donating groups in both the aryl ring of nitrilimine provided the desired products **(384)** in higher yields. When the C-aryl group of nitilimine was bearing strong electron-withdrawing substituents (-NO_2_), desired product was isolated in higher yields (83 %) whereas, -NO_2_ substituted N-aryl ring resulted in lower yield (36 %) due to the formation of pyrazole *via* cleavage of imidazolone moiety. Notably, when electron donating group at N-aryl and relasing group at C-aryl present, complex mixture was observed due to the opposite electronic effects.

**Scheme 73:**
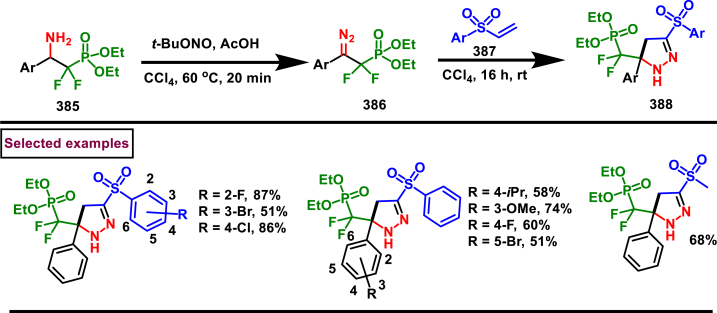
Synthesis of sulfonyl pyrazolines from *in situ* generated diazoalkanes *via* a [3 + 2] cycloaddition.

Reaction of *in situ* generated diazoalkanes bearing difluoromethyl phosphonate (**386**) with vinyl sulfones (**387**) afforded sulfonyl pyrazolines (**388**) tethering difluoromethylphosphonate moiety *via* a [3 + 2] cycloaddition (Han and Roschenthaler et al. 2021) (Scheme 73) [112]. The difluoro diazoalkanes **(386)** were generated *in situ* from the diazotization of β-amino-α,α-difluoroethyl)phosphonates (**385**). It was found that diazo compound **(386)** was unstable and transformed into stable byproduct after cleavage of C-F bond, carbene formation and fluorination. Various aryl vinyl sulfones (**387**) with electron-withdrawing groups furnished the desired products (**388**) in good yields. In contrast, aryl vinyl sulfones containing electron-releasing groups led to lower yields. Diazo alkanes with electron-rich substituents on the aryl group afforded the corresponding pyrazolines (**388**) in good yields. Interestingly, the reaction proved ineffective with other types of alkenes and alkynes such as, dimethyl fumarate, styrene, alkyl propiolates, α-methylstyrenes, and nitrostyrenes.

**Scheme 74:**
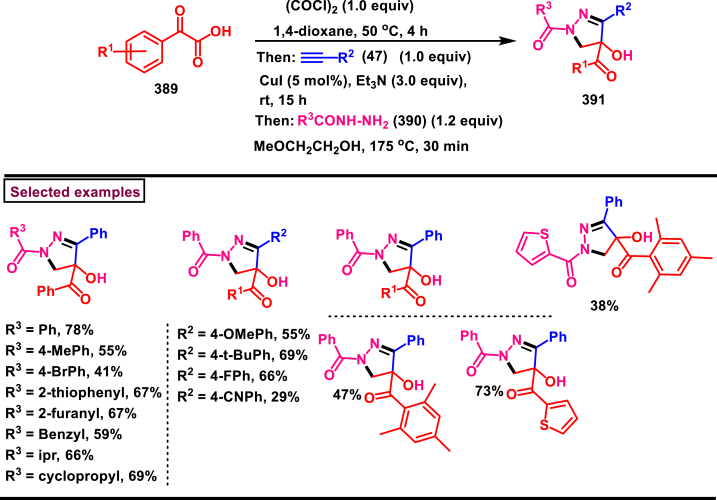
One-pot synthesis of 1,5-diacyl-5-hydroxypyrazolines from a multicomponent reaction.

A series of 1,5-diacyl-5-hydroxypyrazolines **(391)** were prepared from a multicomponent reaction of aryl glyoxylic acids **(389)**, acetylenes **(47)**, hydrazides **(390)** and oxalyl chlorides (Muller et al. 2019) (Scheme 74) [113]. Mechanistically, this process initiated with the formation of arylglyoxylyl chlorides in the presence of oxalyl chloride followed by Cu/base assisted alkynylation and cyclization with hydrazide substrate. This multicomponent synthesis of pyrazolines was successfully performed in an one-pot operation. Both aryl and heteroaryl glyoxylic acids **(389)** were well participated and the desired products were obtained in good yields. Electron rich aryl alkynes **(47)** resulted the desired products in higher yields. On the other hand, aryl, alkyl and benzyl hydrazide **(390)** derivatives were exhibited excellent reactivity towards this transformation and the corresponding pyrazolines **(391)** were isolated in good yields (33%–78 %).

**Scheme 75:**
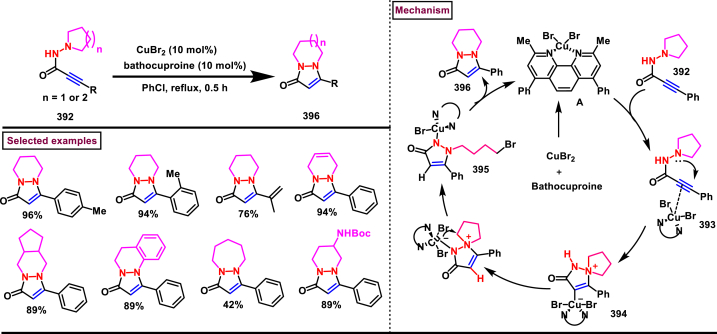
Cu-catalysed synthesis of fused pyrazolones *via* sequential cyclization/migration.

A copper catalysed approach for the preparation of fused pyrazolones (**396**) from the direct reaction of readily available alkynyl hydrazides (**392**) *via* sequential cyclization/migration was reported by Udea et al. in 2020 (Scheme 75) [114]. Optimizational studies revealed that, this reaction highly affected by the temperature, choice of ligand and catalyst loading. Alkynyl hydrazides (**392**) bearing aromatic, polyaromatic, heteroaryl and alkyl substituents were tolerated and the corresponding pyrazolones (**396**) were obtained in good yield. End products were subjected to chemical transformations and synthetically useful compounds are generated. Notably, pyrazolone (**396**) was converted into thiopyrazolone in the presence of Lawesson's reagent in good yields. A series of control experiments suggested that, activation of alkyne unit by Cu-Ligand complex **(A)** was followed by 5-*endo-dig* cyclization to afford spiro intermediate **(394)**. Further, 1,3-*H* shift and generation of alkyl bromide *via* intramolecular attack formed **(395)**. Desired pyrazole **(396)** was released with the regeneration of Cu-catalyst. Overall, this transformation was proceeded through nucleophilic addition/1,3-migration and 1,2-migration process.

**Scheme 76:**
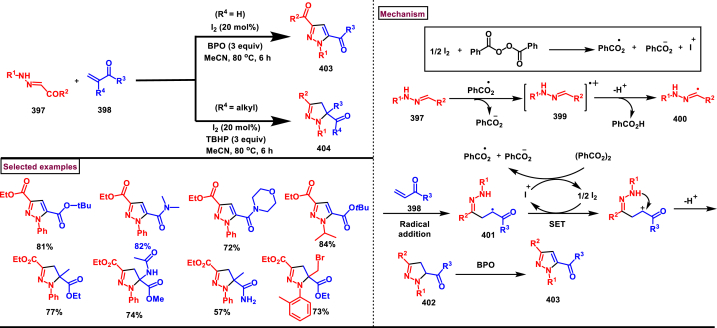
Regioselective synthesis of pyrazoles *via* an I_2_-catalysed oxidative cyclization.

In 2019, Zhao et al. reported a convenient regioselective preparation of multi-substituted dihydropyrazoles (**404**) and pyrazoles (**403**) from electron-deficient alkenes (**398**) and aldehyde hydrazones (**397**) *via* an I_2_-catalysed oxidative cyclization protocol (Scheme 76) [115]. Optimized reaction conditions were identified by varying parameters such as, oxidant, temperature and solvents. Scope of this radical relay process was studied with various aldehyde aryl hydrazones and alkyl hydrazones (**397**) and the corresponding products were obtained in excellent yields. In the case of olefines, only electron deficient alkenes were feasible. In the presence of BPO and I_2_, diversified pyrazoles (**403**) were obtained. α-alkylated acrylates in presence of TBHP and I_2_, resulted in dihydropyrazoles (**404**). Utility of mild oxidant (TBHP) allowed the presence of various sensitive functionalities including free OH and NH groups. The synthetic applicability was further demonstrated by accessing mefenpyr-diethyl (herbicidal activity) in a one-pot operation. Model reactions in the absence of I_2_ led to trace product and addition of TEMPO resulted in no product indicating I_2_-catalysed radical pathway. Mechanistically, the reaction occurred *via* cascade C-H functionalization, formation of C-N bond and sequential oxidation process. Accordingly, radical intermediate **(400)** was formed from the hydrazone **(397)** *via* H-abstraction. Successive addition of radical **(400)** to alkene precursor **(398)** followed by oxidation resulted cationic intermediate which underwent intramolecular cyclization to afford dihydropyrazole **(402)**. BPO-assisted oxidation generated the pyrazole **(403)**.

**Scheme 77:**
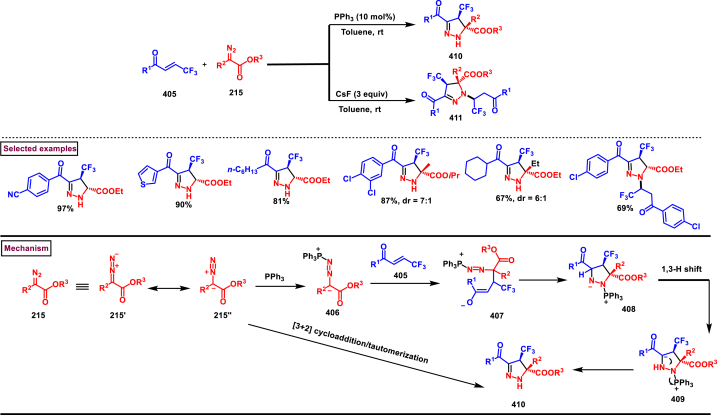
Synthesis of 4-CF_3_ pyrazolines *via* a phosphine catalysed intermolecular [3 + 2] cycloaddition.

Zhang and Liu et al. combinedly reported a facile access to prepare 4-CF_3_ pyrazolines (**410** and **411**) from the β-trifluoromethyl enones (**405**) and α-diazoacetates (**215**) *via* a phosphine catalysed intermolecular [3 + 2] cycloaddition strategy (Scheme 77) [116]. Additionally, a CsF catalysed three component [3 + 2] cycloaddition/Michael addition cascade sequence was also developed to access poly-substituted pyrazolines (**411**) as single diastereomer. β-trifluoromethyl enones with alkyl substitution and diverse substituents on the aryl ring were involved in this transformation and provided the corresponding 4-(trifluoromethyl)-4,5-dihydro-1*H*-pyrazoles **(410)** in good yields. Interestingly, α-alkyl substituted diazo compounds (**215**) were resulted in 90 % isolated yield whereas, the aryl counterpart failed to provide the desired products. Steric hindrance of the diazo precursors was crucial in deciding the diastereoselectivity of the pyrazolines (**410** and **411**). Synthetic value of this method was demonstrated by a scale-up experiment and various synthetic conversions. Mechanistic insights proved that, a resonant form of diazo compound **(215″)** was trapped by PPh_3_ and resulted in **(406)** which simultaneously reacted with enone **(405)** *via* [3 + 2] cycloaddition to give **(407)**. Further cyclization, 1.3-*H* shift, elimination of PPh_3_ afforded the pyrazoline **(410)**.

**Scheme 78:**
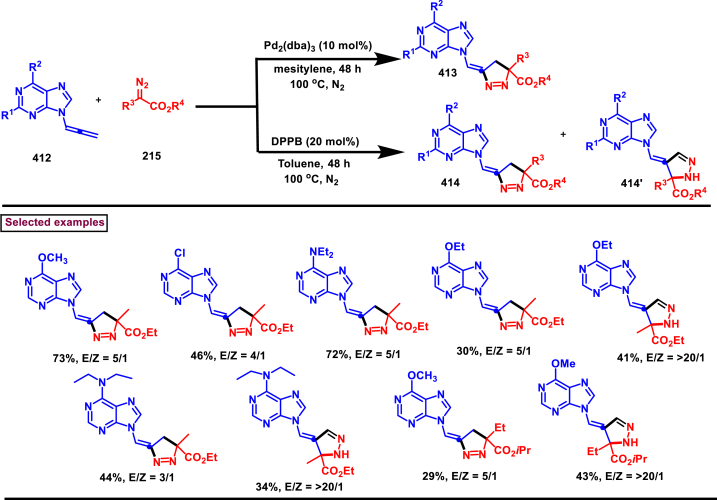
Diversity-oriented synthesis of a pyrazoline derivatives *via* 1,3-dipolar cycloaddition.

In 2018, Guo et al. reported a diversity-oriented preparation of a quaternary carbon containing pyrazoline derivatives (**413** to **414′**) from α-alkyl/aryl diazoesters (**215**) and *N*-purine substituted allene precursors (**412**) *via* transition-metal catalysed 1,3-dipolar cycloaddition (Scheme 78) [117]. Regioselective formation of 1-pyrazolines (**413**) with excellent *E*-selectivity were obtained when Pd_2_(dba)_3_ catalyst was employed. On the other hand, 1-pyrazolines (**414**) and 2-pyrazolines (**414’**) were obtained upon using DPPB. A detailed optimizational studies were carried out by changing the temperature, solvent and metal catalysts. The reaction was found to be general with various allene derivatives (**412**) and α-diazo compounds (**215**) with different ester groups. Notably, when sterically bulky diazo ester was used in presence of DPPB, exclusive formation of 1-pyrazoline was observed. The pyrazoline products were subjected to late-stage modifications and converted into synthetically useful compounds.

### 3.8 Synthesis of fused pyrazoles

#### 3.8.1 Oxocino [2,3-*c*] pyrazoles

**Scheme 79:**
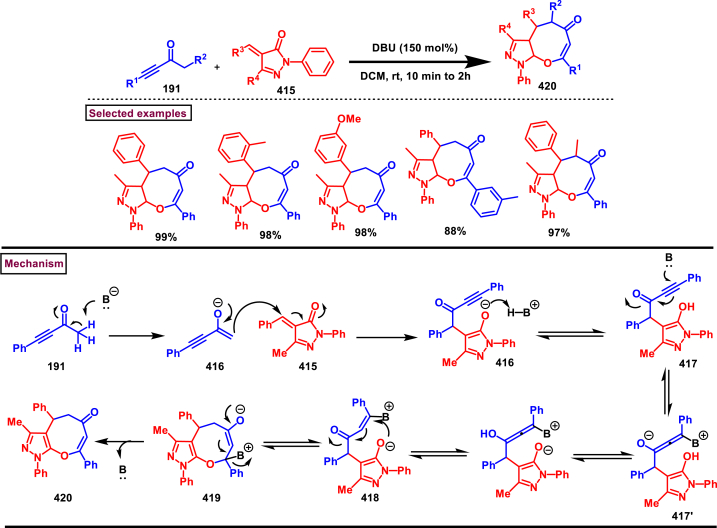
DBU-triggered [4 + 4] domino cycloaddition to synthesize oxocino [2,3-*c*] pyrazoles.

Miao et al. (2018) reported a DBU-triggered [4 + 4] domino cycloaddition sequence to prepare fused eight-membered oxocino [2,3-*c*] pyrazoles (**420**) from ynones (**191**) and benzylidenepyrazolones (**415**) (Scheme 79) [118]. Compared to other existing method to construct the medium sized fused ring skeletons such as, ring-closing metathesis, intramolecular alkylation, ring expansion, retro-Claisen rearrangement, this domino annulation strategy was proved to possess synthetic flexibility and practicality. Under the optimized reaction conditions, the limitations and scope were further explored by treating variously substituted ynones (**191**) and pyrazolones (**415**). It was observed that, pyrazolones bearing aryl, polyaryl or heteroaryl units containing various substitution pattern with different electronic properties were compatible and afforded the corresponding oxocino [2,3-*c*] pyrazoles (**420**) in good yields. To showcase the synthetic utility, the keto-group was diasteroselectively reduced to alcohol using NaBH_4_. Mechanistically, DBU-mediated deprotonation of ynone **(191)** resulted in enolate **(416)** which underwent conjugate addition to C=C bond of benzylidenepyrazolone **(415)** to afford the intermediate **(416)**. Proton abstraction from base resulted in zwitter ionic intermediate **(417** and **417’)** Sequential intramolecular cyclization and tautomerization led to desired eight-membered fused oxocino [2,3-*c*] pyrazoles (**420**) under mild conditions.

#### 3.8.2 1*H*-oxepino [2,3-*c*]pyrazoles

**Scheme 80:**
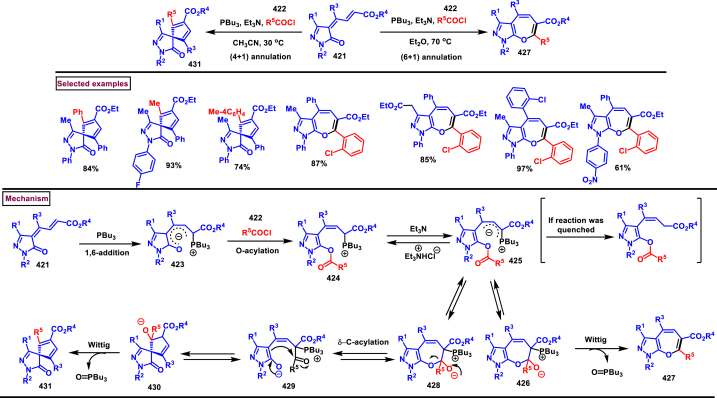
Synthesis of1*H*-oxepino [2,3-*c*]pyrazoles *via* a sequential phospha-1,6-addition/O-acylation and Wittig reaction.

Lin et al. (2020) reported an efficient and diversity-oriented method to access 1*H*-oxepino [2,3-*c*]pyrazoles (**427**) and spiropentadiene pyrazolones **(431)** from conjugated pyrazolones (**421**) and aroylchlorides (**422**) in the presence of PBu_3_ and TEA *via* a sequential phospha-1,6-addition/O-acylation and Wittig reaction in a cascade manner (Scheme 80) [119]. On the other hand, an additional route was developed for the synthesis of spiro-pentadiene pyrazolones **(431)** from the reaction of *α,β,γ,σ*-unsaturated pyrazolones **(421),** acyl chlorides **(422)**, PBu_3_ and Et_3_N *via* a series of phospha-1,6-addition/O-acylation/*δ*-C-acylation/cyclization and Wittig reaction.

Under the optimal conditions, regardless of the steric and electronic nature of the substituents both pyrazolones (**421**) and aroylchlorides (**422**) afforded the desired 1*H*-oxepino[2,3-*c*]pyrazoles (**427**) in excellent yields. It was observed that, presence of sterically hindered substituents in the aroylchlorides favoured the formation of less hindered betaine intermediate which resulted in the spiro-pentadiene pyrazolones **(431)**. Ylide **(425)** was obtained *via* addition of PBu_3_ at *δ*-position of the pyrazolone, O-acylation of zwitter ionic intermediate **(423)** and deprotonation. Intramolecular cyclization afforded the intermediate betaine **(426)** resulted from sterically hindered aroylchlorides underwent Wittig reaction to give 1*H*-oxepino[2,3-*c*]pyrazoles (**427**) whereas, spiroproduct **(431)** was obtained *via* ring opening and *δ*-C-acylation **(428)**, cyclization and Wittig reaction.

**Scheme 81:**
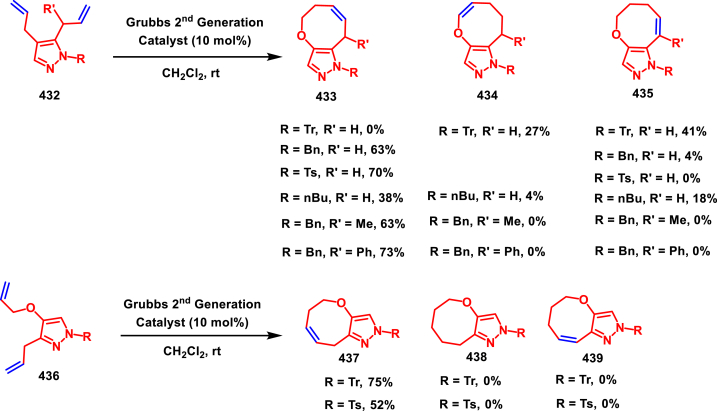
Synthesis of oxepino[3,2-*c*]pyrazoles *via* Claisen rearrangement and RCM.

Usami et al. (2018) reported a general method to prepare dihydro-1*H* or 2*H*-oxepino[3,2-*c*]pyrazoles **(433**–**435** and **437**–**439)** *via* the sequential Claisen rearrangement and RCM of 3-allyl-4-allyloxy-1*H*-pyrazoles **(432)** and 5-allyl-4-allyloxy-1*H*-pyrazoles **(436)** at room temperature (Scheme 81) [120]. Out of three different types of Grubbs catalyst screened, 2nd generation catalyst was found to be more efficient under the reaction conditions. Microwave assisted RCM shortened the reaction time, but the products were obtained together with double bond migration. It was also observed that, ruthenium hydride species was involved in the isomerization process at higher temperature.

**Scheme 82:**
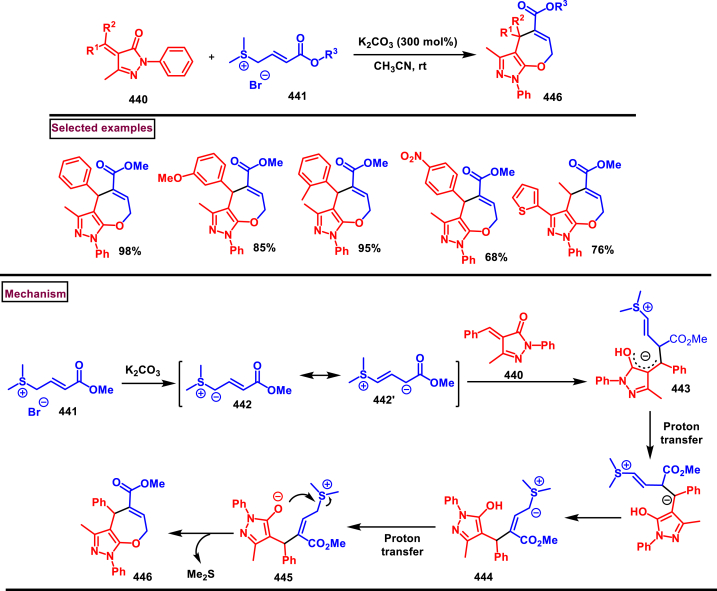
Synthesis of oxepino[3,2-c]pyrazoles *via* base promoted [4 + 3] annulation.

Miao et al. (2018) realized a facile preparation of 4,7-dihydro-1*H*-oxepino[2,3-*c*]pyrazoles **(446)** from crotonate based sulfur ylides **(441)** and α, β-unsaturated pyrazolones **(440)** *via* K_2_CO_3_ promoted [4 + 3] annulation (Scheme 82) [121]. This strategy enabled the convenient access to wide range of oxepino[2,3-*c*]pyrazoles **(446)** from readily available precursors in excellent yields. Unsaturated pyrazolones bearing electronically and sterically distinct functional groups (-Me, -OMe, -F. -Cl -NO_2_) were screened, it was found that electron withdrawing groups led to the anticipated products in slightly lowered yields. Importantly heteroaryl units exhibited excellent scope and furnished the desired products in moderate yields (68–76 %). Base mediated deprotonation of **(441)** resulted in resonant allylic forms **(442** and **442’)** which instantly underwent conjugate nucleophilic addition to **(440)** to generate intermediate **(443)**. Successive proton transfers and intramolecular cyclization with the elimination of Me_2_S afforded the desired product **(446)**. Moreover, this [4 + 3] domino reaction was successfully carried out in gram scale with excellent yields (93 %).

#### 3.8.3 Pyrazolo[1,5-*a*]pyridines

**Scheme 83:**
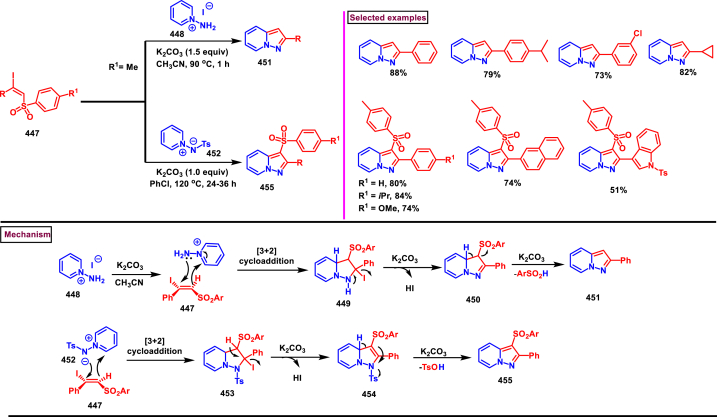
Synthesis of pyrazolo[1,5-*a*]pyridines *via* cyclo-annulation and desulfonylation.

Reddy et al. (2022) disclosed a straight forward approach to achieve pyrazolo[1,5-*a*]pyridines (**451** and **455**) from 1-aminopyridinium iodide (**448**) or *N*-tosylpyridinium imide **(452)** and (*E*)-β-iodovinyl sulfones **(447)** *via* K_2_CO_3_ promoted sequential cyclo-annulation and desulfonylation (Scheme 83) [122]. A series of 2-alkyl/aryl substituted pyrazolo[1,5-*a*]pyridines **(451)** were prepared from differently substituted (*E*)-β-iodovinyl sulfones **(447)** and 1-aminopyridinium iodide **(448)**. No significant effects of position or nature of the substituents on the aryl ring of vinyl sulfone was observed and the anticipated products were obtained in the range of 49–88 % yields. On the other hand, a series of *β*-iodovinyl sulfones **(447)** were smoothly reacted with *N*-tosylpyridinium imides **(452)** and produced 2-aryl-3-tosyl-pyrazolo[1,5-*a*]pyridines **(455)** in high yields. Various control experiments were conducted to support the reaction mechanism.

Free 1-aminopyridine reacted with vinyl sulfones **(447)** *via* [3 + 2] cycloaddition to give the cyclized intermediate **(449)**. Further elimination of HI and tosyl unit afforded **(451)**. Alternatively, **(452)** reacted with vinyl sulfone **(447)** *via* [3 + 2] cycloaddition and the cycloadduct **(453)** was obtained. Desired product **(455)** was formed after the elimination of HI and tosyl group. Overall, gram scale synthesis, high functional group tolerance and transition metal-free conditions are the features of this report.

**Scheme 84:**
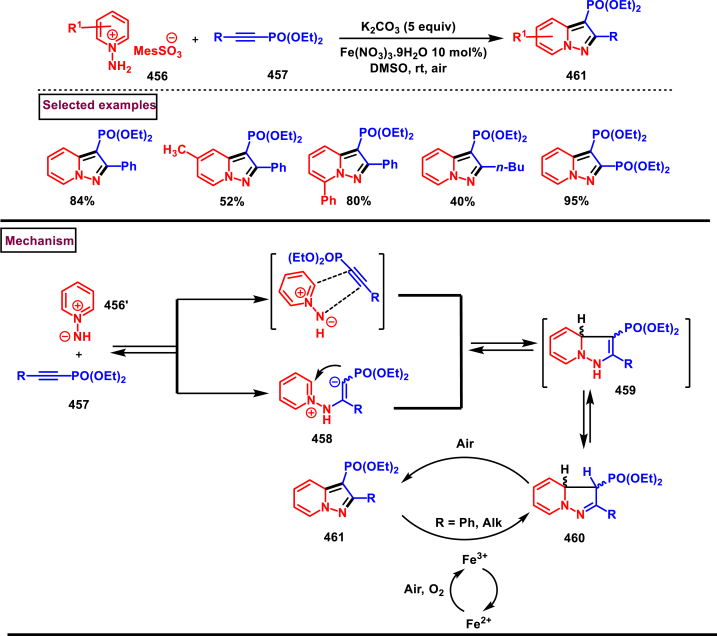
Fe(NO_3_)_3_ catalysed [3 + 2] oxidative cycloaddition.

By treating the *in situ* prepared pyridinium-*N*-imines **(456′)** with 2-functionalized ethynylphosphonates **(457)**, a range of pyrazolo[1,5-*a*]pyridinyl-3-phosphonates **(461)** were obtained in moderate yields *via* [3 + 2] oxidative cycloaddition (Vorob'ev et al. 2022) (Scheme 84) [123]. Fe(NO_3_)_3_ was used as a catalyst in 10 mol% to promote the rection towards higher yields. The reaction was sluggish in the presence of mild electron-donating and withdrawing groups on the aryl ring of *N*-aminopyridinium salts **(456)**. However bulkier Ph group at the *ortho* position led to higher yields (80 %). It was observed that, substituents which lowered the acidity of amino group (4-NMe_2_, quinolinium, isoquinolinium) were completely unreactive under the conditions. Varieties of alkynyl phosphonates **(457)** were tested under the optimized conditions. TMS-substituted phosphonates were exhibited superior reactivity and the corresponding pyrazolo[1,5-*a*]pyridines were obtained with the loss of TMS group. Alkyl and halogenated acetylenes were led to complex mixtures, however OPh-substituted alkynyl phosphonate smoothly participated in the oxidative cycloaddition. Michael addition of deprotonated form **(456′)** with alkynyl phosphonate **(457)** resulted in intermediate **(458)** followed by an intramolecular cyclization gave the cycloadduct **(459)**. Further *H*-shift and Fe-mediated aerial oxidation afforded **(461)**.

**Scheme 85:**
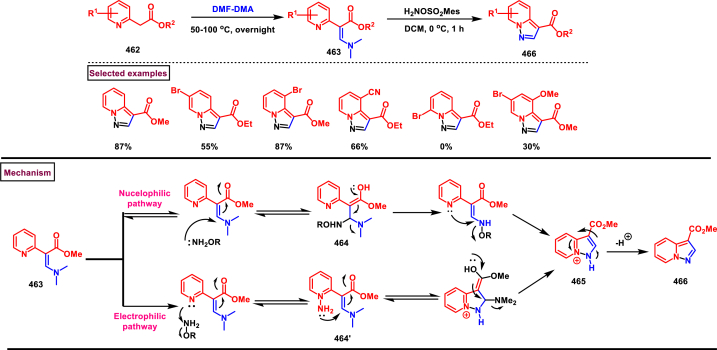
Regiocontrolled synthesis of pyrazolo[1,5-*a*]pyridine from 2-pyridiyl acetates.

A regiocontrolled synthesis of pyrazolo[1,5-*a*]pyridine **(466)** was reported by Mennie et al. (2021) from 2-pyridiyl acetates **(462)** (Scheme 85) [124]. Enamine intermediate **(463)** was obtained from the direct condensation of 2-pyridyl acetates **(462)** with DMF-DMA and further reaction with *O*-(mesitylsulfonyl)-hydroxylamine delivered the desired pyrazolo[1,5-*a*]pyridines **(466)**. Bromo substitution at 4th and 5th position of aryl ring resulted the desired products in moderate yields. 6-bromo substituted 2-pyridyl acetate was inactive under these conditions due to steric issues. Though moderate yields were observed in many cases, excellent regioselectivity was the advantage of this report. Interestingly, utility of 4-pyrimidinyl, 2-quinolinyl acetates and dihydronaphthyridine were led to fused aza analogues in moderate yields. Notably, formation of imidazolopyridine was observed under hydroxylamine/TFAA system from the common enamine intermediate **(463)**. Mechanistically, *O*-(mesitylsulfonyl)hydroxylamine could act as an nucleophile to displace -NMe_2_ unit **(464)** followed by an intramolecular cyclization **(465)** with the release of sulfonyl moiety and deprotonation gave the desired product **(466)**. Alternatively, intermediate **(464’)** would generate when *O*-(mesitylsulfonyl)hydroxylamine act as an electrophile. Sequential cyclization with elimination of -NMe_2_ unit and deprotonation would result the pyrazolo[1,5-*a*]pyridine **(466)**.

**Scheme 86:**
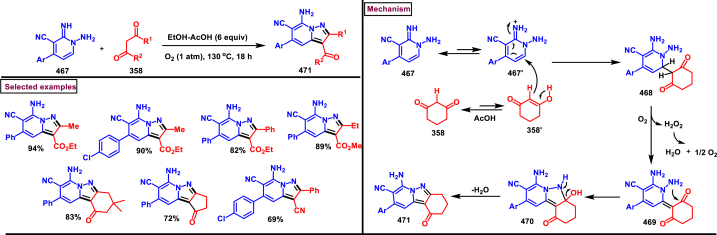
Synthesis of pyrazolo[1,5-*a*]pyridines *via* O_2_/CH_3_COOH triggered oxidative-dehydrogenative coupling.

Molecular O_2_ and Brønsted acid triggered oxidative-dehydrogenative coupling strategy was reported to construct pharmaceutically important pyrazolo[1,5-*a*]pyridines (**471**) from *N*-amino-2-iminopyridines (**467**) and 1,3-dicarbonyl compounds (**358**) (Ibrahim and Behbehani et al.2019) (Scheme 86) [125]. The choice of solvents, acid, oxidant and reaction time were studied in detail. Variations in the aryl-substitution in *N*-amino-2-iminopyridine (**467**) did not have any noticeable effect on the course of the reaction. Considerable variations on 1,3-dicarbonyl compounds (**358**) were also demonstrated by treating ethyl benzoylacetate, ethyl ββ, acetylacetone, methyl propionylacetate, dimedone, 1,3-cyclopentanedione and 1,3-cyclohexanedione with *N*-amino-2-iminopyridines and the corresponding pyrazolo[1,5-*a*]pyridines (**471**) were obtained in good yields. Mechanistically, addition of enol form of 1,3-dicarbonyl compound **(358′)** to acid-activated imino-pyridines **(467’)** resulted in adduct **(468)** O_2_ promoted oxidative dehydrogenation **(469)** and dehydration resulted in pyrazolo[1,5-*a*]pyridines (**471**).

**Scheme 87:**
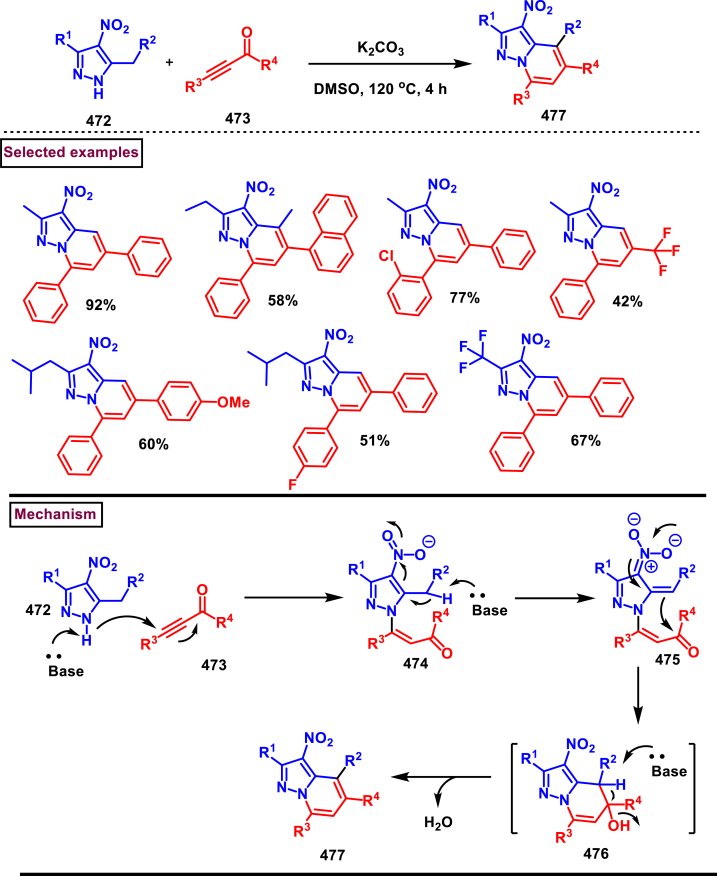
Synthesis of *N*-fused pyrazolo pyridines *via* sequential aza-Michael addition/vinylogous nitroaldol condensation.

DBU-mediated tandem process was developed for the synthesis of substituted *N*-fused pyrazolo pyridines (**477**) from dialkyl-4-nitropyrazoles (**472**) and alkynyl carbonyl compounds (**473**) *via* sequential aza-Michael addition/vinylogous nitroaldol condensation (Suresh et al. 2018) (Scheme 87) [126]. This metal-free approach was proceeded with the formation of C-N and C=C bonds in one step. Generality of this transformation was studied for the 4-nitropyrazoles (**472**) under the extensively optimized conditions. Pyrazoles bearing symmetrical aryl and alkyl substitution at 3, 5 positions delivered the desired *N*-fused 3-nitropyrazolo[1,5-*a*]pyridines (**477**) in moderate to good yields. Sensitive functionalities such as pyridyl, thiophenyl and halogens were survived under the reaction conditions. Similarly, the scope was also studied with unsymmetrically substituted pyrazoles and the corresponding products (**477**) were obtained with excellent regioselectivity due to the participation of less hindered -CH_3_ group in the nitroaldol condensation process. Moderate substrate scope was also exerted for the alkynyl ketones and aldehydes **(473)**. When the NO_2_ group in the pyrazole was replaced with the carboxy unit, only aza-Michael addition product was observed with the trace formation of desired product. *N*-alkylated pyrazoles were also not involved in the reaction and the starting precursors were remained unreacted. It is worth mentioning that, the gram-scale reaction was also successfully demonstrated for this tandem process. Tentative mechanistic proposal described that initial DBU-promoted aza-Michael addition of pyrazole **(472)** on to the alkyne **(473)** furnished the intermediate **(474)**. Sequential abstraction of methylene proton, vinylogous nitroaldol condensation and loss of water furnished the product (**477**) in a regioselective fashion. An alternative pathway proceeding *via* intramolecular 1,8-conjugate addition was also reported.

**Scheme 88:**
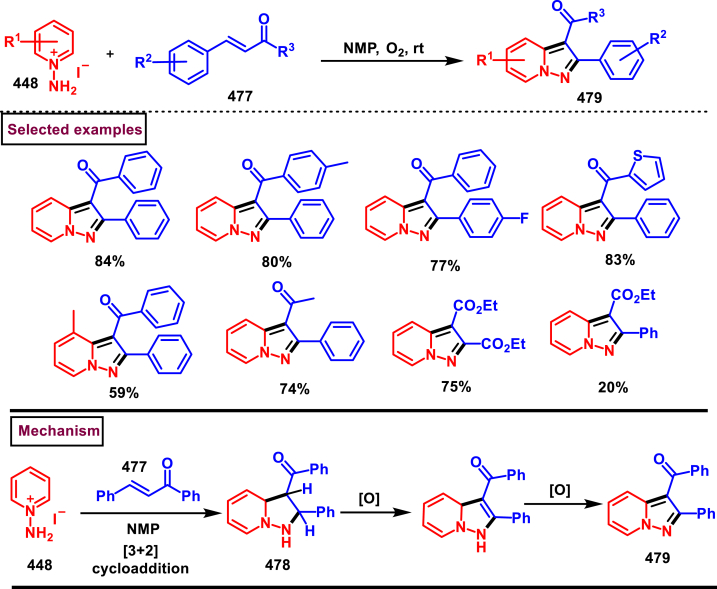
Synthesis of pyrazolo[1,5-*a*]pyridine from *N*-aminopyridines and α,β-unsaturated carbonyl compounds.

Benzoylated and acetylated pyrazolo[1,5-*a*]pyridines **(479)** were obtained *via* oxidative [3 + 2] cycloaddition of α,β-unsaturated carbonyl compounds **(477)** with *N*-aminopyridines **(448)** at room temperature (Adimurthy et al. 2017) (Scheme 88) [127]. This strategy was also extended to electron deficient alkenes to afford functionalized pyrazolo[1,5-*a*]pyridines **(479)** in moderate yields. Various chalcones were engaged the reaction and the corresponding products **(479)** were obtained in good yields (60%–80 %). Conversion was sluggish with NO_2_ substituted chalcones. Utility of benzylideneacetones directly afforded the acetylated products in moderate yields. Furthermore, electron-deficient acrylonitrile, ethyl acrylate, ethyl cinnamate also conveniently afforded the desired pyrazolo[1,5-*a*]pyridines **(479)** at room temperature. NMP assisted [3 + 2] cycloaddition of **(448)** and **(477)** gave the adduct **(478)**. Successive oxidation and aromatization yielded the product **(479)**. Overall, highly challenging insertion of benzoyl/acyl units on the pyrazolo[1,5-*a*]pyridines was successfully achieved under transition metal-free ambient conditions.

#### 3.8.4 Pyrazolo[3,4-*b*]pyridines

**Scheme 89:**
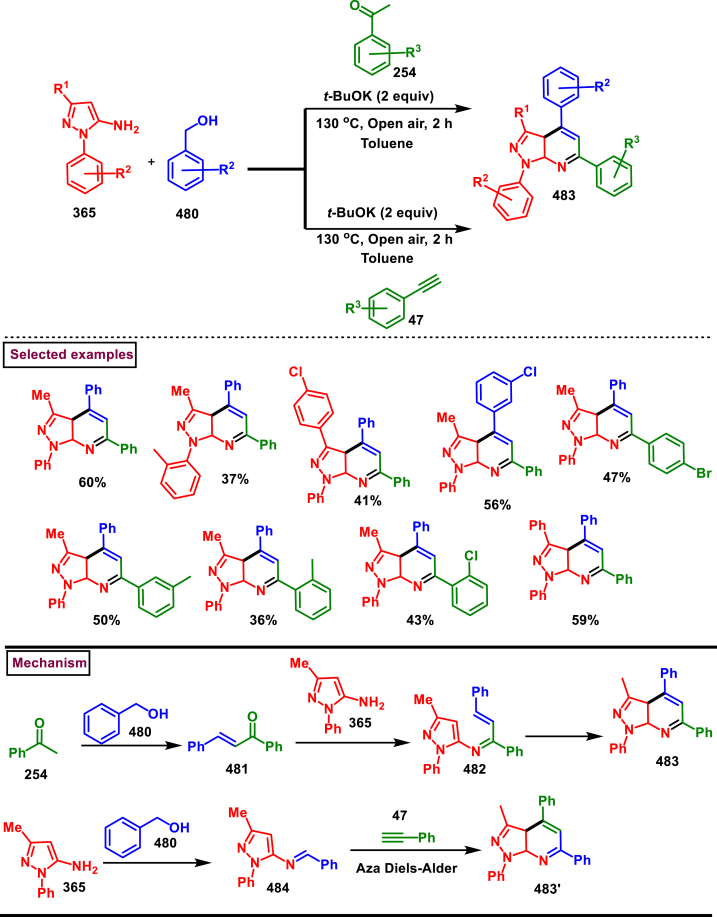
Synthesis of pyrazolo[3,4-*b*]pyridines *via* base/O_2_ mediated *in situ* oxidation.

A base/O_2_ mediated *in situ* oxidation of benzyl alcohols (**480**) into benzaldehydes and its further reaction with arylketones (**254**) and pyrazole amines (**365**) gave the corresponding pyrazolo[3,4-*b*]pyridines (**483**) *via* the formation α, β-unsaturated ketone in moderate to good yields (Ramesh et al. 2023) (Scheme 89) [128]. Moreover, direct dehydrogenative coupling of pyrazole amine (**365**) and benzyl alcohol (**480**) resulted in the imine intermediate **(484)** which further treated with alkynes (**47**) *via* Aza-Diels-Alder fashion to afford pyrazolo-pyridines (**483′**). Amount of base, solvents, temperatures and the substrate ratios were screened in detail. Pyrazole amines (**365**) bearing electron-withdrawing and sterically hindering substituents were led to poor yields. Without any appreciable impact on the yields, variety of substituted benzyl alcohols (**480**) and ketones **(254)** were screened under the optimal conditions, the desired products (**483**) were isolated in moderate to good yields. Remarkably, similar kind of reactivity trends were observed for the aza-Diels-Alder reaction and diverse range of pyrazolo[3,4-*b*]pyridines (**483’**) were obtained. All the intermediates formed during this dehydrogenative cascade process were confirmed by means of a series of control experiments. Further gram-scale synthesis, post-synthetic transformation and the plausible mechanism were also described. A parallel route was developed for the pyrazoline by treating the *α*, *β*-unsaturated ketone intermediate with arylhydrazine hydrochloride.

**Scheme 90:**
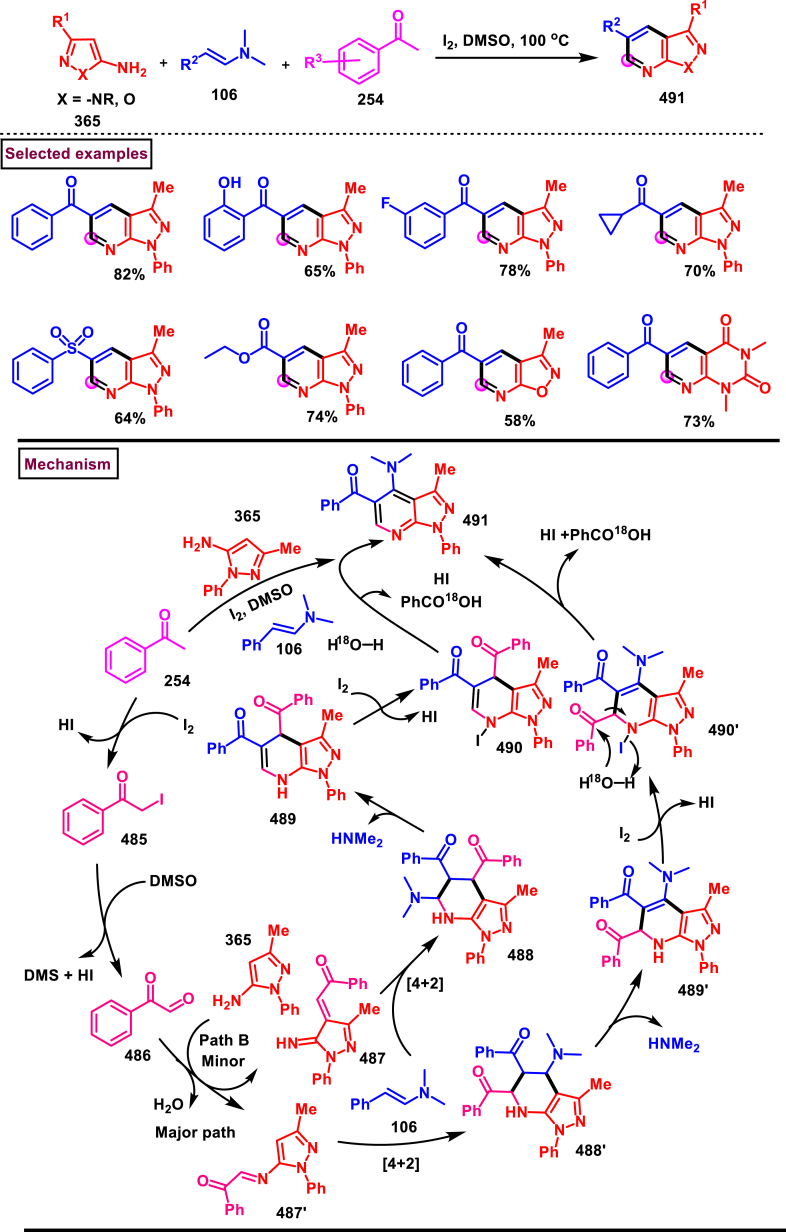
Synthesis of 1*H*-pyrazolo[3,4-*b*]pyridines *via* I_2_ mediated [1 + 3+2] cyclization.

A transition metal-free method was developed to prepare 1*H*-pyrazolo[3,4-*b*]pyridines **(491)** from enaminones **(106)**, 5-aminopyrazoles **(365)**, aryl-methyl ketones **(254)** *via* I_2_ mediated [1 + 3+2] cyclization (Wu et al. 2022) (Scheme 90) [129]. In this process, aryl methyl ketones **(254)** were utilized as C1 source and the cleavage of C(sp^2^)-C(sp^3^) bond was observed. Different classes of enaminones (aryl, heteroaryl, polyaryl) bearing distinct substitutions such as, esters, sulfones at various places were screened and the corresponding products **(491)** were obtained in good yields (65–82 %). Interestingly, cyclic and acyclic alkyl enaminones **(106)** were also found equally reactive. Diverse range of 5-aminopyrazoles, 5-aminooxazoles, 6-aminouracil were smoothly engaged in this process and furnished the desired products in good yields. Plausible two pathway mechanism was proposed, accordingly formation of phenylglyoxal **(486)** was realized *via* I_2_ mediated Kornblum oxidation of iodinated acetophenone **(485)**. Amine **(365)** and **(486)** underwent dehydrative condensation to afford the intermediates **(401** and **401′)**. [4 + 2] condensation of these intermediates with enaminone **(402)** gave the adducts **(487** and **487′)**. Successive elimination of HNMe_2_ and N-H iodination yielded the intermediates **(490** and **490’).** Final reaction of with deuterated water furnished the desired 1*H*-pyrazolo[3,4-*b*]pyridine **(491)**. Various control experiments and post-synthetic modifications of the target 1*H*-pyrazolo[3,4-*b*]pyridines **(491)** into biologically useful molecules were performed to show case the synthetic potential of this method.

**Scheme 91:**
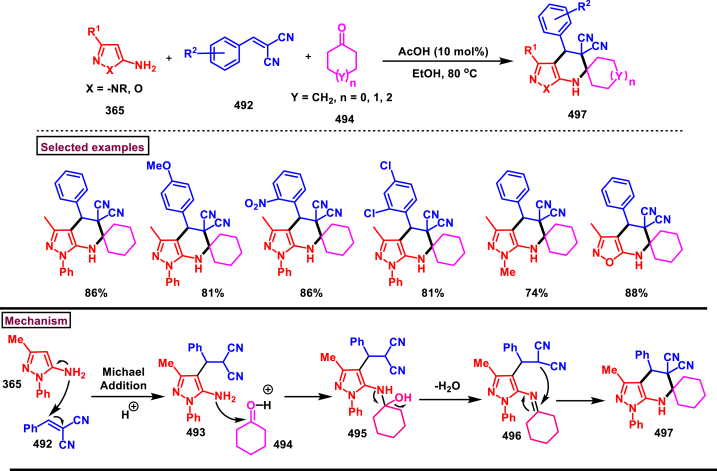
One-pot synthesis of spirocyclic pyrazolo[3,4-*b*]pyridines *via* a domino approach.

A simple domino approach for the preparation of spirocycloalkane embedded pyrazolo[3,4-*b*]pyridine dicarbonitriles **(497)** were prepared *via* a metal free reaction of 2-arylidenemalononitriles **(492)**, cyclic ketones **(494)** and pyrazol-5-amines **(365)** or isoxazole-5-amines (Shen and Zhang et al. 2020) (Scheme 91) [130]. This one-pot method was efficiently catalysed by 10 mol% acetic acid. Steric effect of substituents in the case of 2-arylidenemalononitriles **(492)** was observed as *ortho*-substituents lowered the yields. Strongly electron withdrawing substituents (amide, acid or ester) failed to give the desired products. Common substituents such as, Br, Cl, CF_3_, NO_2_, OMe are survived under the reaction conditions. Diversity was further enhanced with different types of cyclic ketones **(494)**. Among the screened cyclic ketones, cyclohexanone was efficiently involved in this process owing to the stability of the product. It was also reported that, isoxazole-5-amines exhibited high activity than 1*H*-pyrazol-5-amines and provided the anticipated products **(497)** in excellent yields. Mechanistic proposal stated that, AcOH-assisted Michael addition of amine **(365)** with nitrile precursor **(492)** led to intermediate **(493)**. Formation of imine **(496)** was realized by the reaction of **(493)** with the ketone **(494)** followed by dehydration process. Desired product **(497)** was obtained *via* an intramolecular cyclization.

**Scheme 92:**
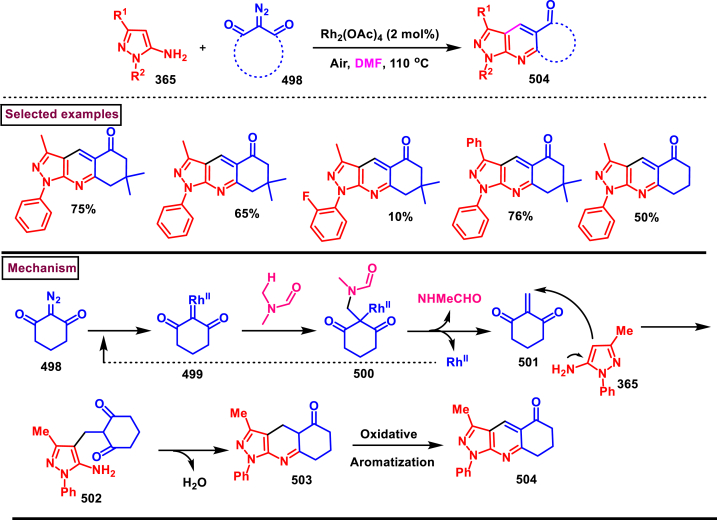
Rh-catalysed synthesis of pyrazolo[3,4-*b*]pyridines *via* [3 + 2+1] cyclization.

Reaction of 1,3-diketone-2-diazo compounds **(498)** and 5-amino pyrazoles **(365)** in the presence of Rh-catalyst resulted in the formation of fused pyrazolo[3,4-*b*]pyridines **(504)** *via* [3 + 2+1] cyclization (Shang et al. 2019) (Scheme 92) [131]. During the pyridine ring formation, DMF served as a source of CH fragment. *N*-alkyl and aryl substituted 1*H*-pyrazol-5-amine derivatives **(365)** were tolerated wide range of substituents (OMe, Me, F, Cl, Br) on the aryl ring and the corresponding products were obtained in moderate to good yields. Severe influence of steric effect was observed as the o-functionalized aryl ring led to very lower yield (10 %). Both alkyl and aryl substitution on the 3rd position of the pyrazole ring gave excellent yields. On the other hand, acyclic 1,3-dicarbonyl-2-diazo compounds were not involved in this transformation, however differently substituted cyclic analogues were led to the desired products **(504)** in good yields. Reaction pathway was explained with the formation key rhodium-carbene species. Generation of rhodium carbene species **(499)** was observed *via* the coordination of Rh (II) catalyst with diazo compound **(498)**. Interaction with C-H bond of DMF **(500)** followed by an elimination resulted in alkene **(501)**. Friedel-Crafts type addition of amine **(365)** to alkene **(501)** gave **(502)**. Further intramolecular condensation and oxidation delivered the product **(504)**.

**Scheme 93:**
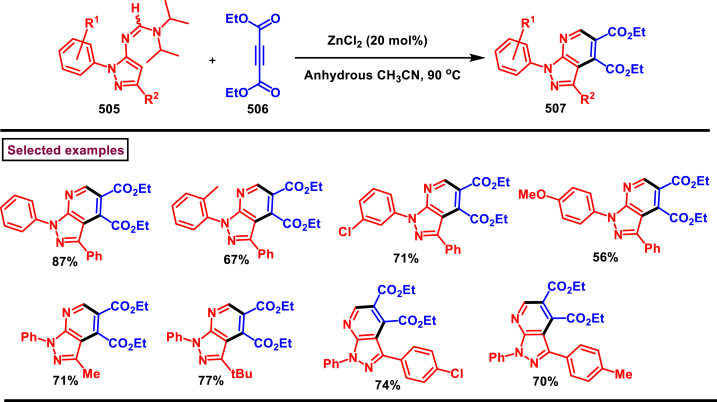
Synthesis of pyrazolo[3,4-*b*]pyridines *via* ZnCl_2_-catalysed aza-Diels-Alder reaction.

Wong and Kuo et al. (2018) reported a convenient method to prepare a diverse range of 1*H*-pyrazolo[3,4-*b*]pyridine-4,5-dicarboxylates **(507)** *via* ZnCl_2_-catalysed [4 + 2] aza-Diels-Alder reaction of acetylenedicarboxylic acid **(506)** and *N*, *N*-disubstituted pyrazoylimines **(505)** (Scheme 93) [132]. Among the various substrates tested, pyrazoylimine **(505)** was found to be more efficient azadiene precursor. N1-phenyl ring of pyrazoylimines with different substitutions such as alkyl, alkoxy, and halogens were screened and the desired products were obtained in good yields. Similarly, alkyl and aryl groups at C3 position of pyrazole resulted the pyrazolo[3,4-*b*]pyridines **(507)** in acceptable yields.

#### 3.8.5 Pyrazolo[5,1-*a*]isoquinolines

**Scheme 94:**
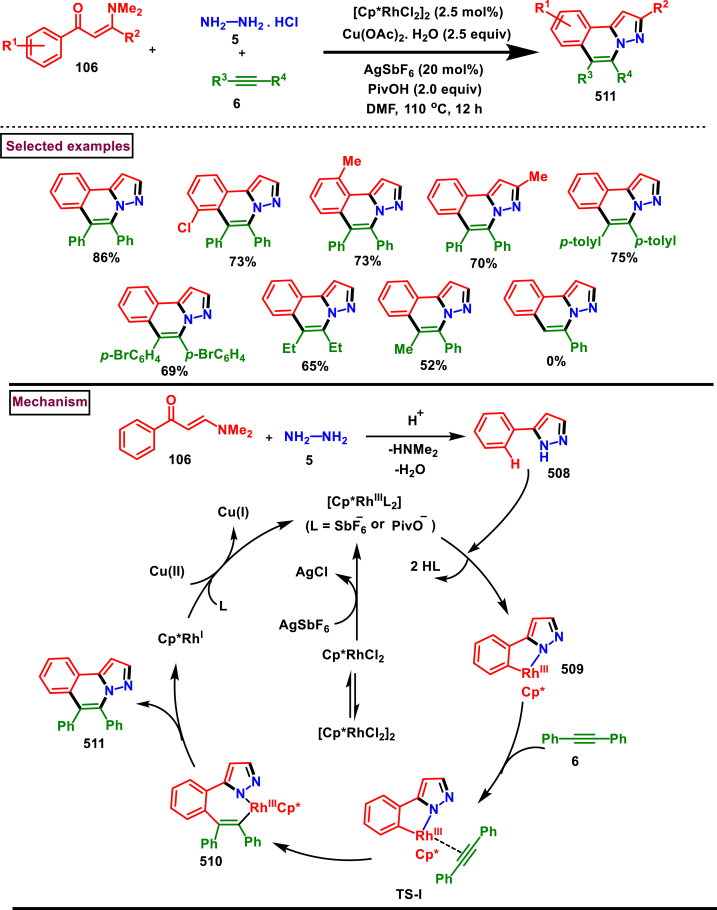
Rh-catalysed synthesis of tricyclic fused pyrazolo [5,1-*a*]isoquinolines.

Rh-catalysed practical approach was developed for the preparation of tricyclic fused pyrazolo [5,1-*a*]isoquinolines (**511**) from the direct reaction of *N*, *N*-dimethyl enaminones (**106**), internal alkynes (**6**) and hydrazine hydrochloride (**5**) (Wan and Liu et al. 2023) (Scheme 94) [133]. This one-step cascade approach constructed simultaneously two cycles through the formation of one C-C bond and three C-N bonds. Optimizational studies revealed that, the presence of Cu(II) salt, Ag(I) salt, Rh-catalysts and additive (PivOH) were necessary. Excellent functional group tolerance and wide substitution patterns were observed with varieties of enaminones (**106**) and alkynes (**6**). Though unsymmetrically substituted internal alkynes gave the fused pyrazolo [5,1-*a*]isoquinolines (**511**) in moderate yield, the terminal alkynes were found to be unsuccessful. Control experiments proved the 5-arylpyrazoles **(508)** was the main intermediate which is very important for the further aryl C-H annulation. Proposed mechanism stated that, *in situ* generated 5-arylpyrazoles **(508)** interacted with active Rh^III^ species to generate intermediate **(509)** which was coordinated with internal alkyne and transformed into intermediate **(510)** *via* TS-I. Further reductive elimination through C-N bond formation resulted the desired product (**511**) and released Rh(I) species. Cu(II) oxidized Rh(I) into Rh(III) species in the presence of ligands.

**Scheme 95:**
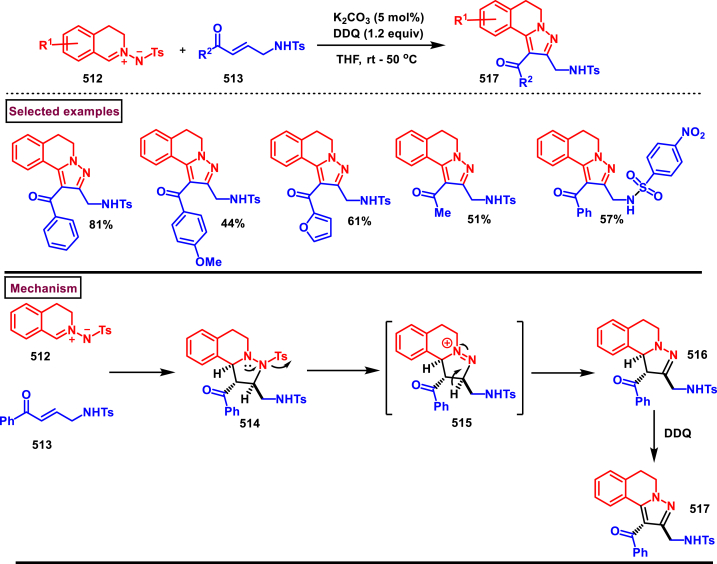
Synthesis of 5,6-dihydropyrazolo[5,1-*a*]isoquinolines *via* [3 + 2] cycloaddition.

Kim et al. (2023) developed an one-pot tandem approach for the synthesis of densely substituted 5,6-dihydropyrazolo[5,1-*a*]isoquinolines **(517)** from the reaction of α,β-unsaturated ketones **(513)** and C, N-cyclic azomethine imines **(512)** (Scheme 95) [134]. This strategy involved a sequential [3 + 2] cycloaddition of **(512)** and **(513)** to give the adduct **(516)** *via* elimination of tosyl group. DDQ assisted oxidative aromatization released the desired product **(517)**. K_2_CO_3_ served as an economic choice of base and DDQ was used as an oxidant. Irrespective of the position and electronic behaviour, various substituents on the aryl ring of *N*-tosyl protected azomethine imine **(512)** were tolerated and the corresponding products **(517)** were obtained in good yields (51%–81 %). In the case of enones, electron-withdrawing groups at the *para*-position proved more effective. Varieties of aryl enones and an example of alkyl variant **(513)** were tested. Reasonable variations were done for the arylsulfonyl protecting groups and the desired products **(517)** were obtained in acceptable yields.

**Scheme 96:**
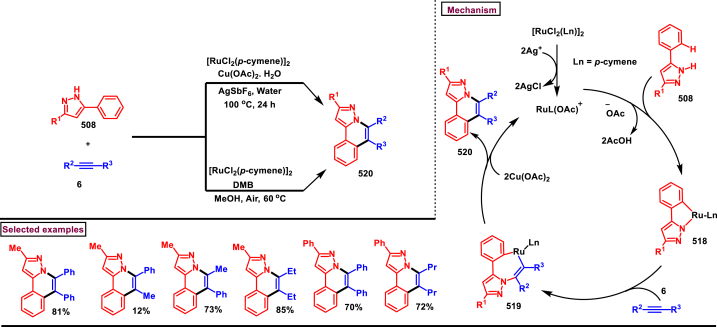
Synthesis of pyrazolo[5,1-*a*]isoquinolines *via* Ru-catalysed annulation reactions.

Ru(II)-catalysed annulation of functionalized pyrazoles **(508)** with alkynes **(6)** produced pyrazolo[5,1-*a*]isoquinolines **(520)** in the presence of AgSbF_6_ and Cu(OAc)_2_.H_2_O *via* sequential C-H/N-H bond cleavage (Singh et al. 2023) (Scheme 96) [135]. Notably, this transformation occurred in water. Additionally, this reaction could also be performed at low temperature in methanol solvent without Cu(OAc)_2_.H_2_O in the presence of a carboxylate ligand. Lower yield was observed in the absence of silver-based additive as it was crucially involved in the generation of cationic [Ru(OAc)/(*p*-cymene)]^+^ species. On the other hand, in methanol the reaction proceed with the formation of neutral [(*p*-cymene)(Ru(OAc)_2_] species. Terminal alkynes (phenyl acetylene) and electron deficient internal alkynes were not involved in this transformation. Varieties of electron rich internal alkynes **(6)** smoothly participated in this process and corresponding annulated products **(520)** were obtained in excellent yields. Insertion of active Ru-species into aryl C-H of pyrazole **(508)** resulted in organoruthenium intermediate **(518)**. Interaction and coordination of alkyne unit **(6)** resulted in intermediate **(519)**. Further reductive elimination released the product **(520)**. Cu(OAc)_2_.H_2_O oxidized Ru-(0) species.

**Scheme 97:**
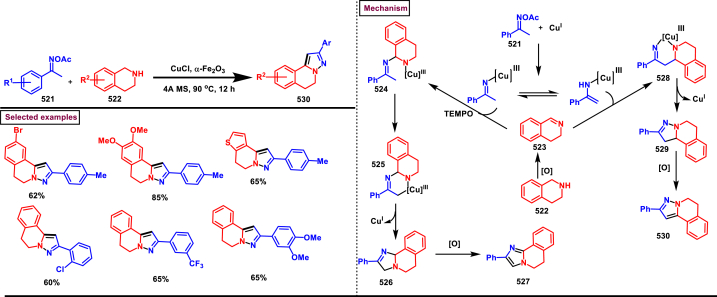
Cu-catalysed oxidative [3 + 2] annulation strategy to access isoquinolines-fused pyrazoles.

A formal oxidative [3 + 2] annulation strategy between tetrahydroisoquinolines (**522**) and ketoxime acetates (**521**) to access isoquinolines-fused pyrazoles (**530**) was developed under copper catalysis (Huang et al. 2019) (Scheme 97) [136]. Delightfully, it is the first documented oxidative [3 + 2] annulation of tetrahydroisoquinolines (**522**) and ketoxime acetate (**521**). A systematic examination of various reaction parameters was screened. A facile access to 2-substituted 5,6-dihydropyrazolo[5,1-*α*]isoquinolines (**530**) with ketoximes (**521**) bearing various functional groups such as halogens, nitro, CF_3_ was achieved under the optimal conditions. Electronic nature of the aryl ring in ketoximes had little effect on the outcome, whereas steric hindrance in the substrates led to lower yields. Generality of 1,2,3,4-tetrahydroisoquinoline (**522**) was revealed that, electronic nature of aryl substituents had no effect on the yield. However, sterically hindered functionalities led to poor yields. Control experiments revealed that, the transformation occurred through the dihydropyrazole intermediate and its dehydrative aromatization. Oxime acetate **(521)** was converted into Cu(III)-imino species which were tautomerized and nucelophilically reacted with **(523)** resulted in intermediate **(528)**. Subsequent reductive elimination and aromatization gave the pyrazoline **(529)** which on oxidation transformed into desired pyrazole **(530)**. Parallelly, authors have also investigated the formation of functionalized imidazole derivatives **(527)** *via* TEMPO-promoted oxidative annulation of substituted 1,2,3,4-tetrahydroisoquinolines (**522**) and oxime acetates (**521**).

**Scheme 98:**
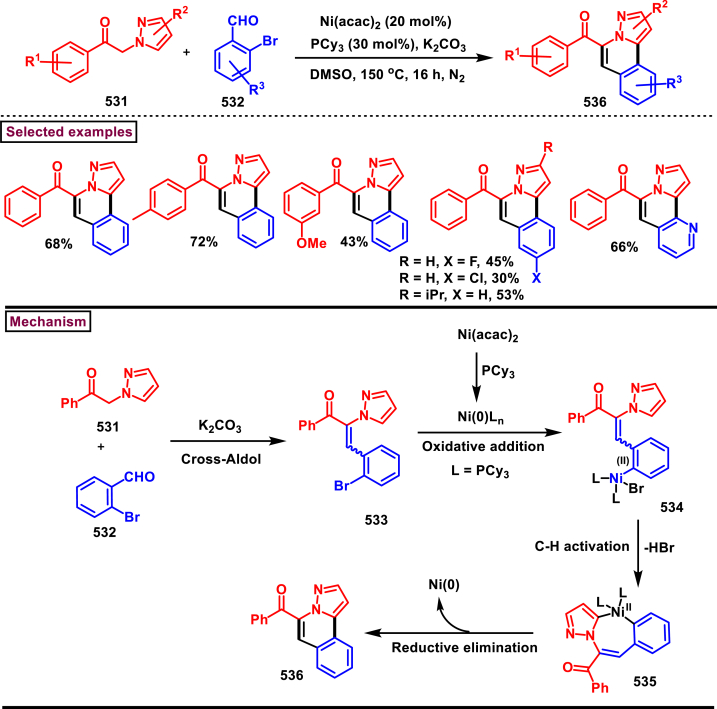
Synthesis of pyrazolo[5,1-*a*]isoquinolines *via* Ni-catalysed intramolecular arylation.

In 2018, Kumar et al. reported a straight forward Ni-catalysed preparation of pyrazolo[5,1-*a*]isoquinolines **(536)** from 2-bromo aldehydes **(532)** and 1-aryl-2-(1*H*-pyrazol-1-yl)ethan-1-ones **(531)** in good yields (Scheme 98) [137]. Mechanistically, Knoevenagel condensation of ketone **(531)** and aldehyde **(532)** afforded alkene **(533)**. Oxidative addition of **(533)** with active Ni(0) catalyst gave the intermediate **(534)**. Sequential intramolecular C-H activation and reductive elimination resulted the product **(536)**. Scope of the reaction with a series of 1-aryl-2-(1*H*-pyrazol-1-yl)ethan-1-ones **(531)** revealed that, electronically neutral and rich substituents favoured the transformation and resulted in higher yields (up to 70 %) whereas, the electron withdrawing groups afforded the corresponding products in lower yields (32 %). Even the substituent effect on the pyrazole ring was studied with various groups. Aryl, heteroaryl bromo-aldehydes **(532)** bearing electronically distinct functional groups were found equally effective and led to the desired products **(536)** in moderate to good yields. Experimental supports were obtained from various control experiments.

**Scheme 99:**
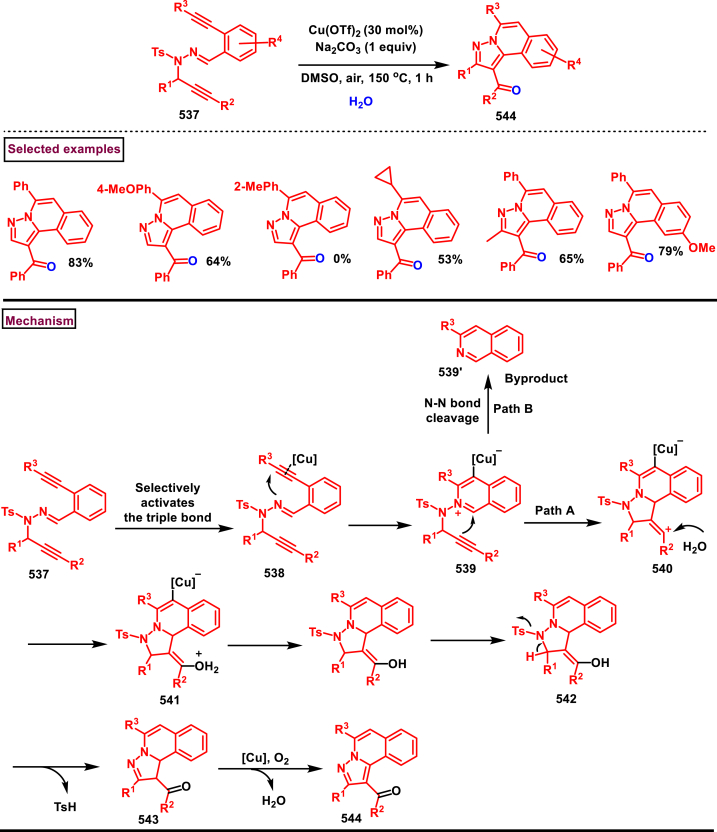
Cu-catalysed regioselective preparation of pyrazolo[5,1-*a*]isoquinolines.

Cu-catalysed regioselective preparation of pyrazolo[5,1-*a*]isoquinolines **(544)** was achieved *via* a cascade bicyclization of *N*-propargylic sulfonylhydrazones **(537)** (Zhan et al. 2017) (Scheme 99) [138]. Interestingly, presence of H_2_O was essential for the successful formation of desired product, as the reaction failed to initiate in super-dry DMSO solvent. This hydration assisted strategy occurred through the formation of cyclized intermediate **(539)** *via* an intramolecular N-nucleophilic attack of imine to Cu-activated alkyne unit. Tricyclic intermediate **(540)** bearing vinyl cation was generated through another nucleophilic reaction. Rapid trapping of vinyl cation by H_2_O **(541)**, followed by demetallation, Keto-enol tautomerization **(543)**, Cu-catalysed oxidation and aromatization afforded the product **(544)**. It was also observed that, **(539’)** was formed from **(539)** *via* N-N bond cleavage. Various aryl and non-aryl units at R^3^ were smoothly participated, however sterically hindered *o*-substituents failed to give the desired products. Most of the common substituents such as -Me, -OMe, -Cl were found compatible at the aryl ring (R^4^). Substitution at R^2^ was crucial as only electronically weaker groups (-Cl, -Me) gave the desired products **(544)** in good yields. Electron withdrawing groups and alkyl units were failed to give the anticipated products. A three-step synthesis of a compound with potential CDC25B, PTP1B, TC-PTP inhibition was prepared to showcase the potential of this method.

**Scheme 100:**
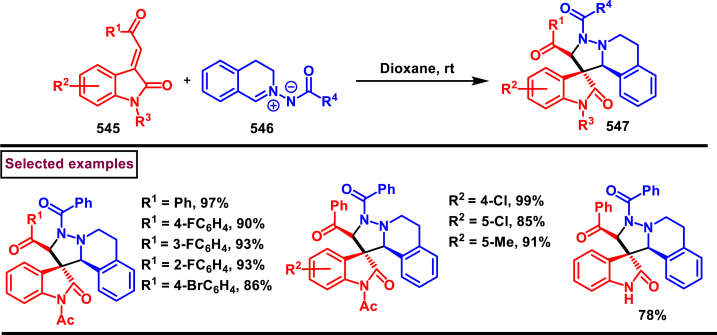
Synthesis of spiro[indoline-3,1′-pyrazolo[5,1-*a*]isoquinolines *via* transition metal-free 1, 3-dipolar cycloaddition.

Construction of spiro[indoline-3,1′-pyrazolo[5,1-*a*]isoquinolines **(547)** was realized from C, N-cyclic azomethines **(546)** and methyleneindolinones **(545)** *via* transition metal-free 1, 3-dipolar cycloaddition (Zhang et al. 2017) (Scheme 100) [139]. Variety of substituted aryl and alkyl ketones at R^1^ efficiently participated in this transformation and products **(547)** were obtained in excellent yields (84%–98 %). Aryl ring of oxindole nucleus **(545)** was also screened with sensitive substitutions. Notably, presence of free NH on methyleneindolinone did not affect the outcome of the reaction and good yield was obtained (78 %). On the other hand, both alkyl and aryl groups at R^4^ of C, N-cyclic azomethines **(546)** were tested and desired products were formed in good yields. Overall, this room temperature process exhibited good functional group tolerance and broad range of densely functionalized spirocyclic pyrazolo[5,1-*a*]isoquinolines **(547)** were obtained.

#### 3.8.6 Pyrazolo[1,5-*c*]quinazolines

**Scheme 101:**
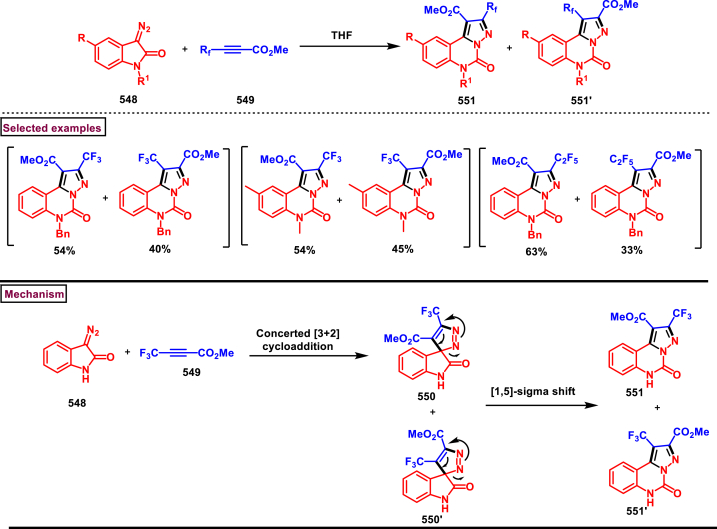
Fluoroalkylated pyrazolo[1,5-*c*]quinazolines from methyl *β*-fluoroalkylpropionates and 3-diazoindolin-2-ones.

In 2023, Tang and Cao et al. reported an efficient method to construct fluoroalkylated pyrazolo[1,5-*c*]quinazolines **(551** and **551’)** from methyl *β*-fluoroalkylpropionates **(549)** and 3-diazoindolin-2-ones **(548)** *via* a [3 + 2] cycloaddition (Scheme 101) [140]. Scope of the reaction was studied with variously substituted 3-diazoindolin-2-ones **(548)**. Aryl group of **(548)** substituted with electron donating groups (-CH_3_, -OCH_3_) or electron withdrawing groups (-F, -Cl, -NO_2_) provided the mixture of regioisomers **(551)** in excellent yields. It was observed that, desired products **(551)** were obtained with *N*-Bn or *N*-alkyl protection or free N-H of indoline-2-one **(548)**. On the other hand, *β*-difluoro, pentafluoro, heptafluoropropylpropiolates were also smoothly reacted in this process. Notably, perfluoroalkyl groups enhanced the reactivity of alkyne precursors. [3 + 2] concerted cycloaddition was proposed for this transformation.

**Scheme 102:**
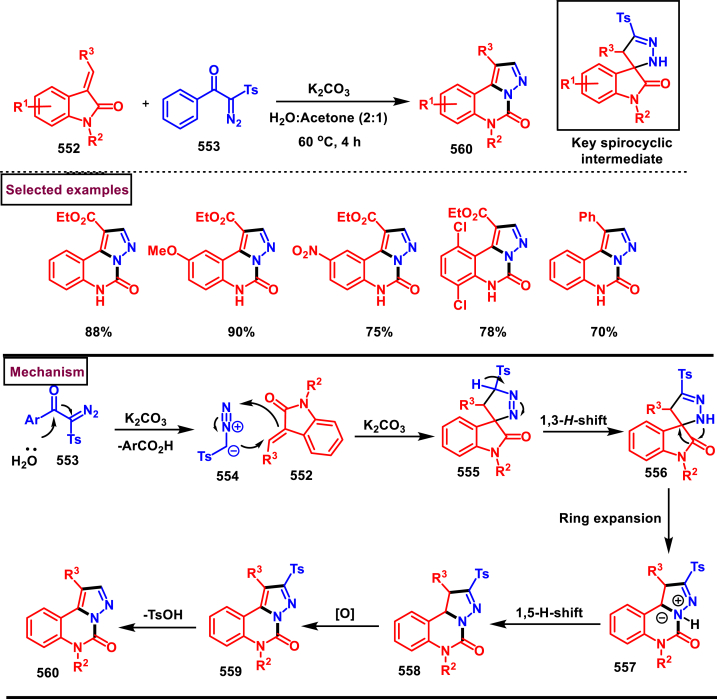
Synthesis of pyrazolo[1,5-*c*]quinazolines from tosyldiazomethane and 3-ylideneoxindoles.

A mild and transition metal-free method was reported by Nagendra Babu et al. (2020) to prepare pyrazolo-[1,5-*c*]quinazolines **(560)** from tosyldiazomethane **(553)** and 3-ylideneoxindoles **(552)** in presence of H_2_O as a green solvent (Scheme 102) [141]. H_2_O played an important role in the *in situ* generation of 1,3-dipole **(554)** which underwent rapid cycloaddition with alkene substrate **(552)**. Mechanistic studies revealed that, this transformation involved 1,3-dipolar cycloaddition **(555)**, 1,3-*H* shift **(556)**, regioselective ring expansion of spiro intermediate **(557)**, oxidative aromatization **(559)** and elimination of Ts group as TsOH **(560)**. Generality was tested with variously substituted 3-ylideneoxindoles **(552)** (-Me, -OMe, -Br, -Cl, -NO_2_) and the corresponding pyrazolo-[1,5-*c*]quinazolines **(560)** were obtained in moderate to good yields (60%–90 %). Electron donating substituents on the aryl ring favoured the reaction towards higher yields than electron-withdrawing groups.

**Scheme 103:**
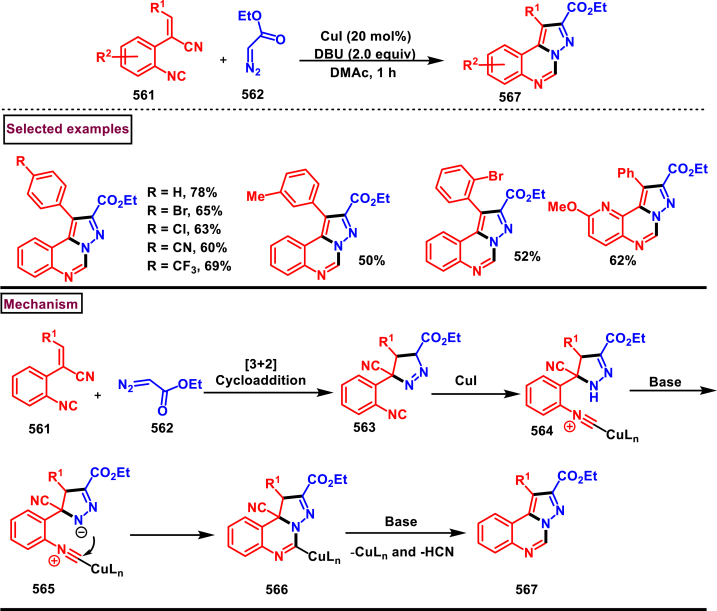
Synthesis of pyrazolo[1,5-*c*]quinazolines from Cu-catalysed reaction of alkenyl aryl isocyanides and diazo compounds.

Wang and Li et al. (2020) combinedly reported a domino reaction of diazo compounds **(562)** with *o*-alkenyl aryl isocyanides **(561)** under copper catalysed conditions to prepare a series of pyrazolo-[1,5-*c*]quinazolines **(567)** under mild conditions (Scheme 103) [142]. This strategy proceeded efficiently with readily available substrate with consecutive formation of three bonds and two rings in one-step. Reaction mechanism involved an initial [3 + 2] cycloaddition to afford **(563)**, Coordination of Cu with isocyanide unit resulted in *N*-aryl nitrilium intermediate **(564)**. Further intramolecular cyclization **(566)** and elimination of HCN, furnished the corresponding pyrazolo-[1,5-*c*]quinazolines **(467)**. Another pathway with the initial elimination of HCN was also elucidated. Aryl/heteroaryl isocyanides **(561)** bearing electronically different groups (-F, -Cl, -CF_3_) at various positions smoothly reacted with diazo compounds **(562)** to furnish the pyrazolo-[1,5-*c*]quinazolines **(567)** in acceptable yields (46%–78 %). On the other hand, α-diazocarbonyl compounds bearing sensitive functionalities such as, terminal alkynes, alkenes, esters, ketones were reacted successfully to give the desired products in 27%–84 % yields. Overall, wide substrate scope with respect to both the precursors was observed.

**Scheme 104:**
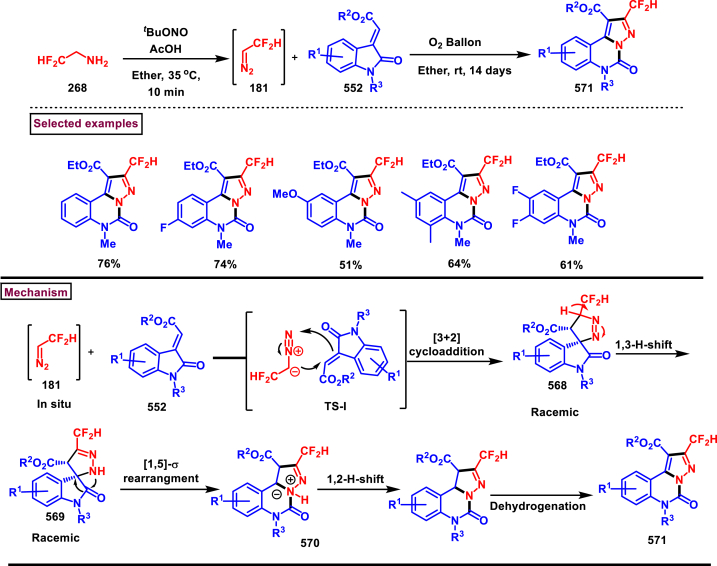
Synthesis of pyrazolo[1,5-*c*]quinazolines *via* a [3 + 2] cycloaddition/1,3-*H* shift/rearrangement and dehydrogenation process.

A catalyst and additive free protocol was developed to access pyrazolo[1,5-*c*]quinazolines bearing CF_2_H (**571**) from CF_2_HCHN_2_ (**181**) and 3-ylideoxindoles (**552**) (Chen and Han et al. 2018) (Scheme 104) [143]. This cascade approach proceeded at room temperature *via* a [3 + 2] cycloaddition/1,3-H shift/rearrangement and dehydrogenation process. Difluoromethyl diazomethane (**181**) was *in situ* generated from the reaction of difluoroethylamine (**268**) and *t*-BuONO and served as an effective 1,3-dipole. Utilization of O_2_ as an external oxidant promoted the oxidative aromatization step and increased the overall yield. Without appreciable changes in the yield, various substituted 3-ylideneoxindoles (**552**) with wide range of N-protecting groups led to the desired products in satisfactory yields. Control experiments proved that; this transformation proceeded *via* formation of a spirocyclic intermediate **(568)**. According to the proposed mechanism, *in situ* generated **(181)** underwent [3 + 2] cycloaddition with oxindole **(552)** to give **(568)** *via* TS-I. Sequential 1,3-*H* shift and a 1,5-sigmatropic rearrangement resulted in the tricyclic intermediate **(570)**. Desired product was obtained through 1,2-*H* shift and dehydrogenative aromatization.

#### 3.8.7 Chromeno-pyrazoles

**Scheme 105:**
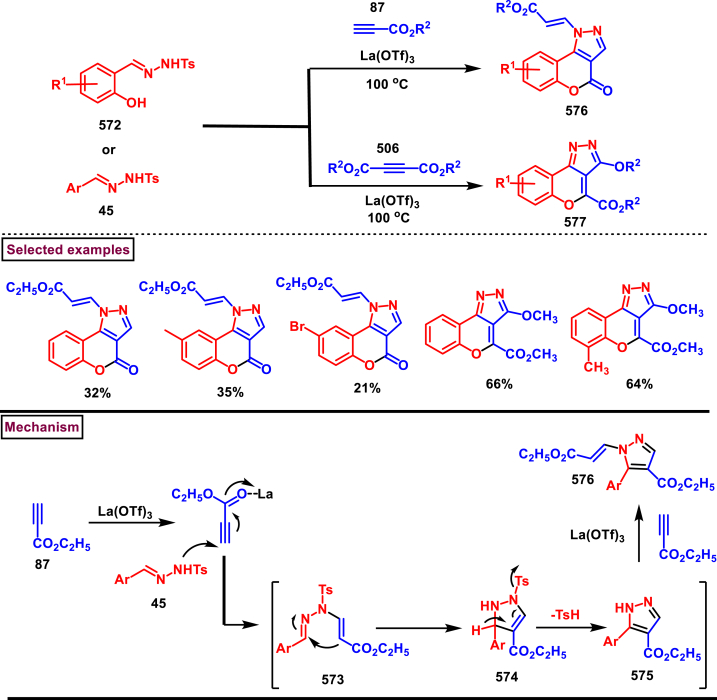
La(OTf)_3_-catalysed synthesis of fused chromenopyrazoles and five membered-*N*-alkenylpyrazoles.

A La(OTf)_3_-catalysed stereo- and regioselective approach for the synthesis of fused chromenopyrazoles (**577**) and five membered-*N*-alkenylpyrazoles **(576)** was developed from reaction of salicylic *N*-tosylhydrazones (**572**) and *N*-tosylhydrazones **(45)** *via* a sequential formation of C-C, C-N and C-O bonds (Bhimapaka et al. 2021) (Scheme 105) [144]. After screening various temperatures and catalysts, it was observed that the best results were obtained under neat conditions. When activated alkynes (**87**) were treated with electron-withdrawing and electron-donating hydrazone derivative, corresponding chromenopyrazole acrylates (**576**) were obtained in moderate yields. Remarkably, when alkyne-dioates (**506**) were employed a new product chromenopyrazole-4-carboxylates (**577**) were obtained in moderate to good yields. Mechanistically, La(OTf)_3_ activated the carbonyl group of alkyne (**87**) which underwent aza-Michael addition with hydrazone (**45**) to give the intermediate **(573)**. Further intramolecular cyclization and elimination of tosyl group resulted in pyrazole **(575)**. (*E*)-selective **(576)** was obtained *via* aza Michael addition with propiolate **(87)**.

**Scheme 106:**
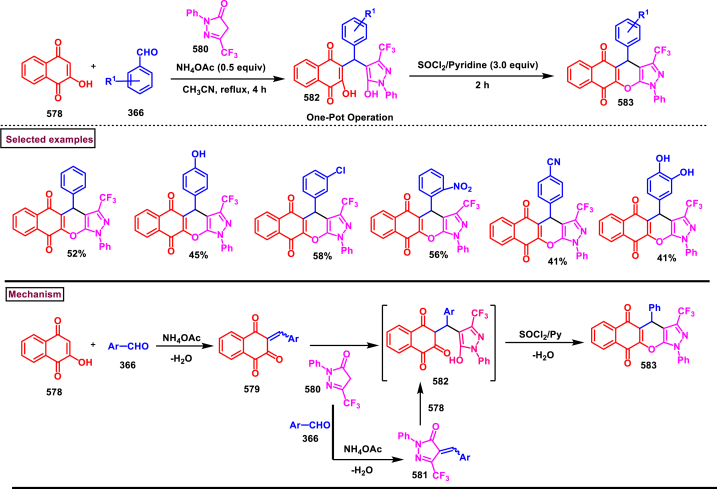
One-pot synthesis of CF_3_-bearing benzo [6,7]chromeno[2,3-*c*]pyrazoles.

A multi-component reaction to access CF_3_-bearing benzo [6,7]chromeno[2,3-*c*]pyrazoles **(583)** was developed by Song et al. (2020) in two steps (Scheme 106) [145]. Firstly, NH_4_OAc catalysed reaction of aromatic aldehydes **(366)**, 1-aryl-3-trifluoromethyl-5-pyrazolone **(580)** and 2-hydroxy-1,4-naphthoquinones **(578)** resulted in a non-annulated trifluoromethylpyrazolone tethered methane derivatives **(582)** which upon dehydration using SOCl_2_/pyridine furnished annulated chromeno[2,3-*c*]pyrazoles **(583)** *via* intramolecular dehydrative annulation. Aryl aldehydes bearing electron-withdrawing groups were found more reactive than the electron-releasing substrates. Heteroaryl and alkyl aldehydes were not involved in this reaction. NH_4_OAc assisted Knoevenagel condensation of **(578)** and **(366)** gave the alkene intermediate **(579)**. Michael addition of pyrazolone **(580)** with intermediate **(579)** resulted the hydroxy-ketone **(582)**. Intramolecular dehydrative cyclization in the presence of SOCl_2_/pyridine gave the desired tetracyclic product **(583)**. Alternatively, Knoevenagel adduct **(581)** could also react with **(578)** and lead to the intermediate **(582)**. Overall, this one-pot, two-steps process was highly advantageous in terms of high atom economy and convenient operation.

**Scheme 107:**
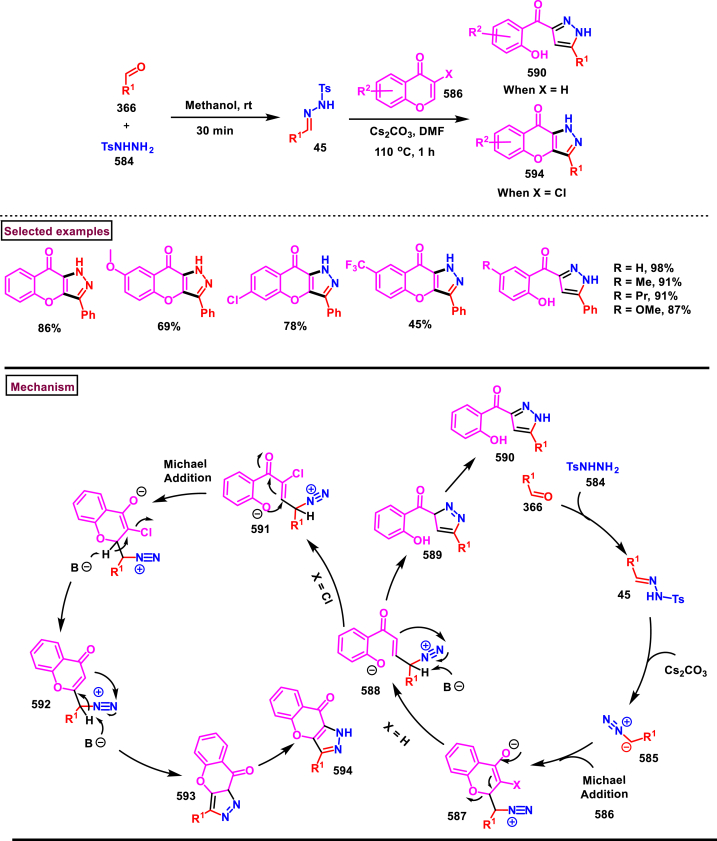
One-pot synthesis of chromeno[3,2-*c*]pyrazoles from 3-chlorochromenes.

In 2019, Yang and Zhang et al. reported a one-pot method to chemoselectively synthesize fused chromeno[3,2-*c*]pyrazoles **(594)** from aldehydes **(366)**, tosyl hydrazides **(584)**, and 3-chlorochromenes **(586)** (Scheme 107) [146]. Interestingly, monocyclic pyrazole skeletons **(590)** were obtained by utilizing unsubstituted chromones under similar conditions. Electronic nature of the substituents on the aryl ring of chromene **(586)** was affected the yield as the electron-donating groups favoured the reaction more than electron withdrawing substituents. NO_2_ substituted aryl aldehyde led to no desired products, whereas alkyl aldehyde furnished the anticipated product in lower yield (27 %). Wide substrate scope was exerted to afford the densely functionalized pyrazoles **(590** and **594)** in excellent yields. Base mediated formation of **(585)** and its Michael addition with **(586)** gave the bicyclic adduct **(587)**. 3-unsubstituted **(588)** underwent intramolecular cyclization and 1,3-*H*-shift to afford monocyclic pyrroles **(590)**. In the case of 3-chloro substituted chromenes, **(588)** proceeded *via* intramolecular Michael addition, base assisted dehydrochlorination and keto-enol tautomerization to furnish **(592)**. Another set of intramolecular cyclization and 1,5-*H* shift delivered the tricyclic fused pyrrole **(594)**.

**Scheme 108:**
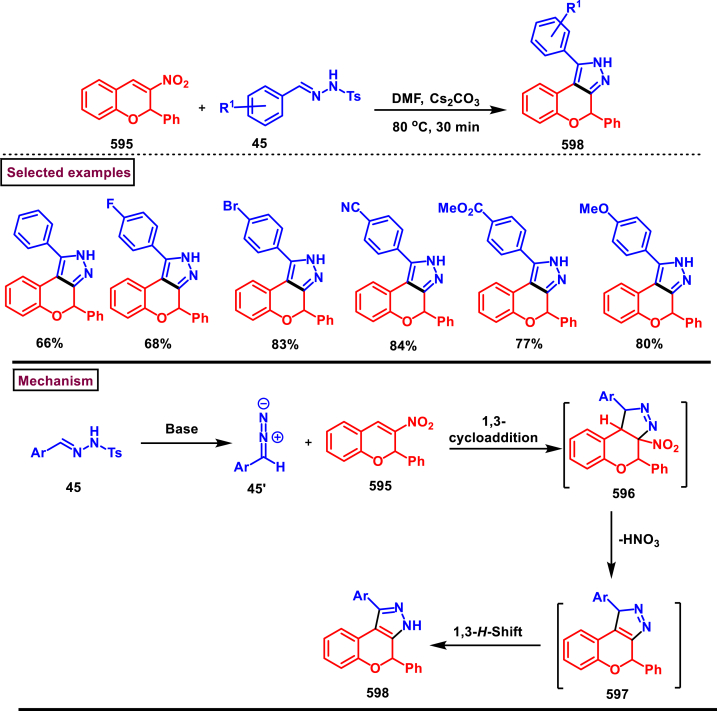
Synthesis of 2,4-dihydrochromeno[3,4-*c*]pyrazoles *via* base mediated intermolecular 1,3-dipolar cycloaddition.

Base mediated construction of 2,4-dihydrochromeno[3,4-*c*]pyrazoles **(598)** was realized *via* the intermolecular 1,3-dipolar cycloaddition of 3-nitro-2-phenyl-2*H*-chromene **(595)** and *N*-tosylhydrazones **(45)** (Li et al. 2017) (Scheme 108) [147]. A highly reactive diazo species **(45’)** was generated *in situ* from the *N*-tosylhydrazones **(45)** in the presence base. A series of electron rich and poor functional groups on the *N*-tosyl hydrazones (-CN, -CO_2_Me, -Br, -Cl, -F) were studied under these conditions and the corresponding products were obtained in moderate to good yields. Mechanistically, initial 1,3-dipolar cycloaddition **(596)**, followed by a nitrous acid elimination **(597)** and 1,3-*H* shift occurred to deliver the 2,4-dihydrochromeno[3,4-*c*]pyrazoles **(598)**.

#### 3.8.8 Pyrazolo[1,2-*a*]cinnoline

**Scheme 109:**
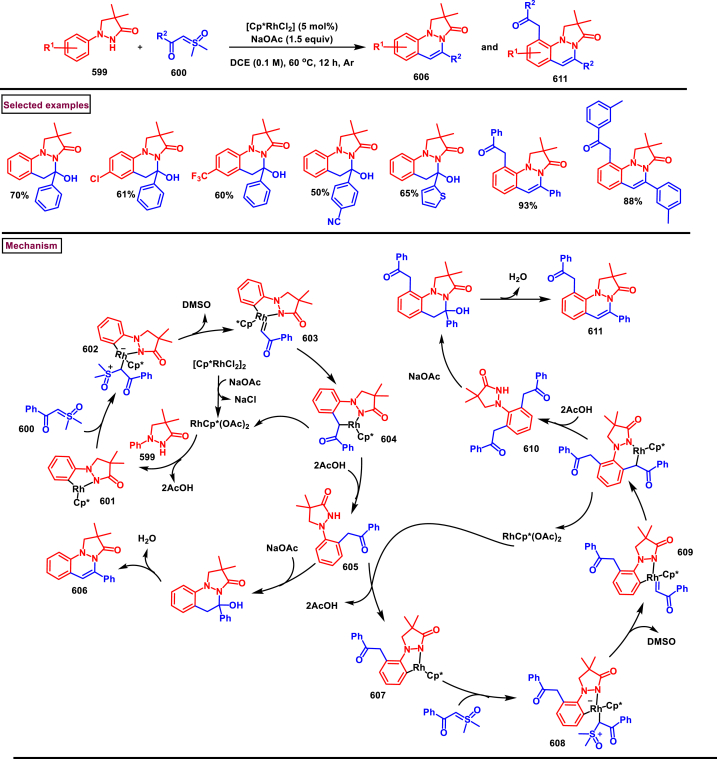
Synthesis of pyrazolo[1,2-*a*]cinnolines *via* Rh(III)-catalysed [4 + 2] cyclization.

An efficient method to prepare pyrazolo[1,2-*a*]cinnolines **(606** and **611)** from pyrazolidinones **(599)** and sulfoxonium ylides **(600)** was developed under Rh(III)-catalysed conditions (Wang and Liu et al. 2021) (Scheme 109) [148]. Desired cinnolines were obtained through C-H activation of pyrazolidinones **(599)** under Rh(III)-catalysis followed by a [4 + 2] cyclization of sulfoxonium ylides **(600)**. Exclusive formation of 5,10-disubstituted **(611)** or 5-substituted pyrazolo[1,2-*a*]cinnolines **(606)** was realized under slightly modified reaction conditions. Introduction of electronically distinct substituents (-Me, -OMe, -NO_2_, -Br, -Cl) at various positions of pyrazolidinones gave the desired products **(606** and **611)** without much impact on the yields (46–71 %). Similarly, excellent substrate scope was exhibited by various alkyl, aryl and benzyl sulfoxonium ylides **(600)** and the corresponding cinnolines were obtained in good yields (69 % tom79 %). It was observed that, alkyl sulfoxonium ylides were reacted better than aryl ylides. Mechanistic insights were obtained from various control experiments such as, deuteration studies, KIE studies and competition experiments. According to path A, Rh-catalysed aryl C-H activation and N-H deprotonation generated the five membered rhodium intermediate **(601)**. Further coordination with **(600)** and elimination of DMSO gave the intermediate **(603)**. Formation of six membered Rh-intermediate *via* migratory insertion of **(604)** followed by acidic exposure (AcOH) furnished ketone **(605)** which underwent intramolecular cyclization and dehydration to give the product **(606)**. Pathway B proceeded *via* the formation of rhodacycle at another *ortho* position of ketone intermediate **(607)**. Further complexation of alkene unit of **(600)** with Rh(III) and elimination of DMSO delivered the intermediate **(609)**. A series of migratory insertion, protonolysis, intramolecular cyclization and dehydration generated the product **(611)**.

**Scheme 110:**
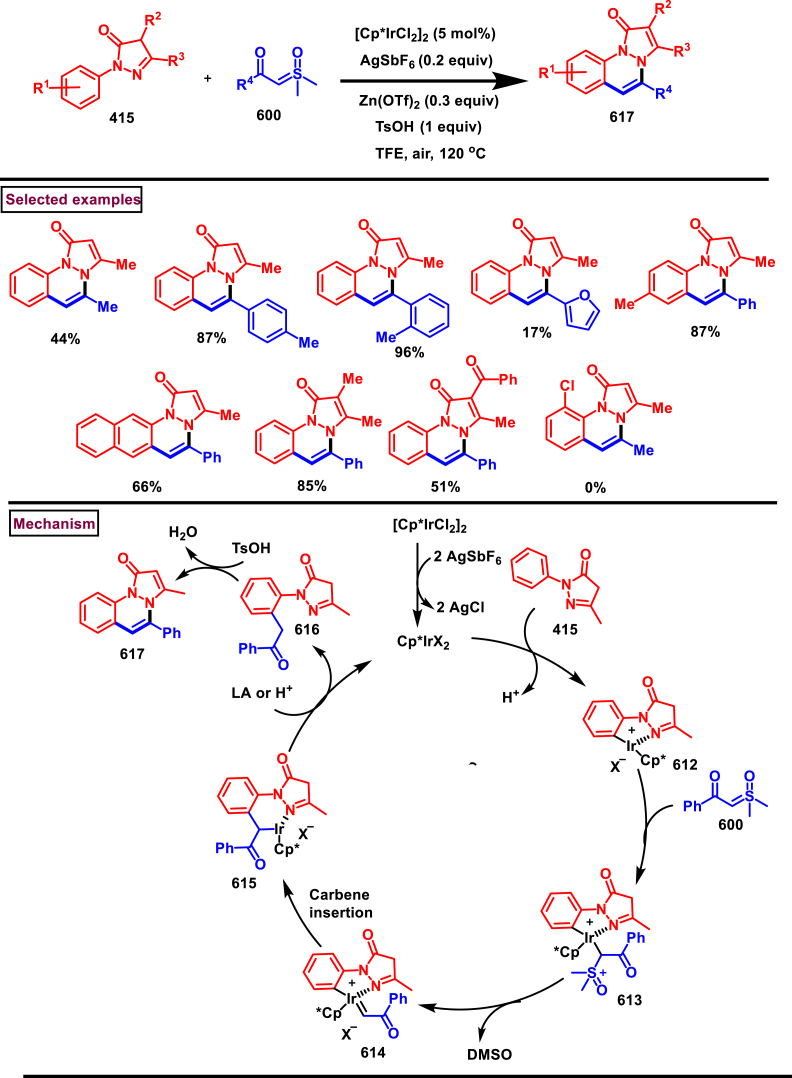
Ir-catalysed synthesis of pyrazolo[1,2-*α*]cinnolines *via* C-H activation and annulation strategy.

Ir-catalysed cascade reaction of sulfoxonium ylides (**600**) and pyrazolones (**415**) was performed to access pyrazolo[1,2-*a*]cinnoline (**617**) derivatives *via* C-H activation and annulation strategy (Dong et al. 2019) (Scheme 110) [149]. Optimal condition was established with catalytic [IrCp∗Cl_2_]_2_, Zn(OTf)_2_ and additive (AgSbF_6_). Without much impact on the yield, variously substituted aryl/heteroaryl rings on the sulfoxonium ylides (**600**) tollerated well and the desired products (**617**) were isolated in excellent yields. However, sterically demanding substituents were failed to give the annulated products. On the other hand, regardless of the electronic nature of the substituents in the *N*-aryl group of the pyrazolone, annulation products (**617**) were obtained with excellent regioselectivity. Similar to sulfoxonium ylides, sterically hindered substrates (*ortho* substituted) were not yielded the desired pyrazolo[1,2-*a*]cinnoline derivatives. Base on various control experiments, a plausible mechanism was proposed in which the active Ir^III^ catalyst involved in the C-H activation of the *N*-aryl pyrazolone (**415**) and subsequent coordination with sulfoxonium ylide (**600**) followed by DMSO elimination generated the intermediate **(614)**. A series of migratory insertion **(615)**, protonalysis **(616)**, intramolecular cyclization and dehydration afforded the desired product **(617)**.

**Scheme 111:**
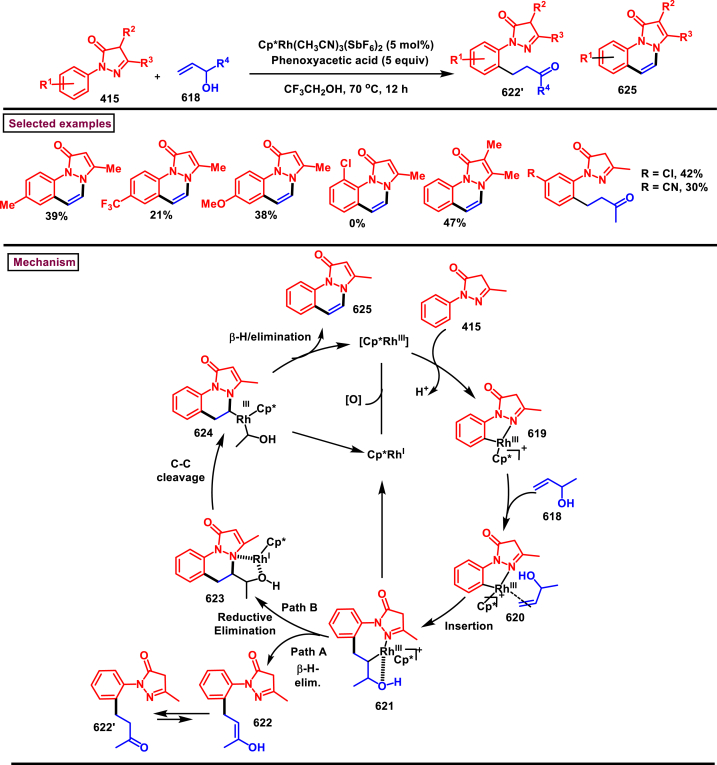
Synthesis of pyrazolo[1,2-*a*]cinnolines *via* Rh(III)-catalysed C-H activation of allylic alcohols.

Dong and Xu et al. (2019) reported a novel method to prepare pyrazolo[1,2-*a*]cinnolines **(625)** from 3-methyl-1-phenyl-1*H*-pyrazol-5-(4*H*)-ones **(415)** and allylic alcohol **(618)** under Rh(III)-catalysed C-H activation conditions (Scheme 111) [150]. *γ*-ketone bearing edaravone derivatives **(622′)** were also obtained together with the cinnolines. Substrate scope was explored with wide range of *N*-arylpyrazol-5-ones **(415)**. Electron withdrawing substituents at the *para*-position led to lower yields (0 %–21 %) compared to electron-donating groups (31–39 %). Utility of allylic alcohol **(618)** led to the formation of only pyrazolo[1,2-*a*]cinnoline **(625)** in 44 % yield whereas, other functionalized allylic alcohols resulted in mixture of cinnolines and alkylated ketones. Based on the control experimental studies, a possible reaction pathway also proposed. Initial *ortho*-metalation and *N*-coordination gave the rhodocycle intermediate **(619)**. Interaction with allylic alcohol **(620)** followed by a migratory insertion provided the intermediate **(621)**. β-hydride elimination offered **(622’)** *via* a keto-enol tautomerization (Path A). In path B, intermediate **(621)** underwent reductive elimination and C-C cleavage to give **(624)**. Desired product **(625)** was observed though another *β*-hydride elimination and the Rh(I)-species was re-oxidized to Rh(III)-catalyst for another cycle.

**Scheme 112:**
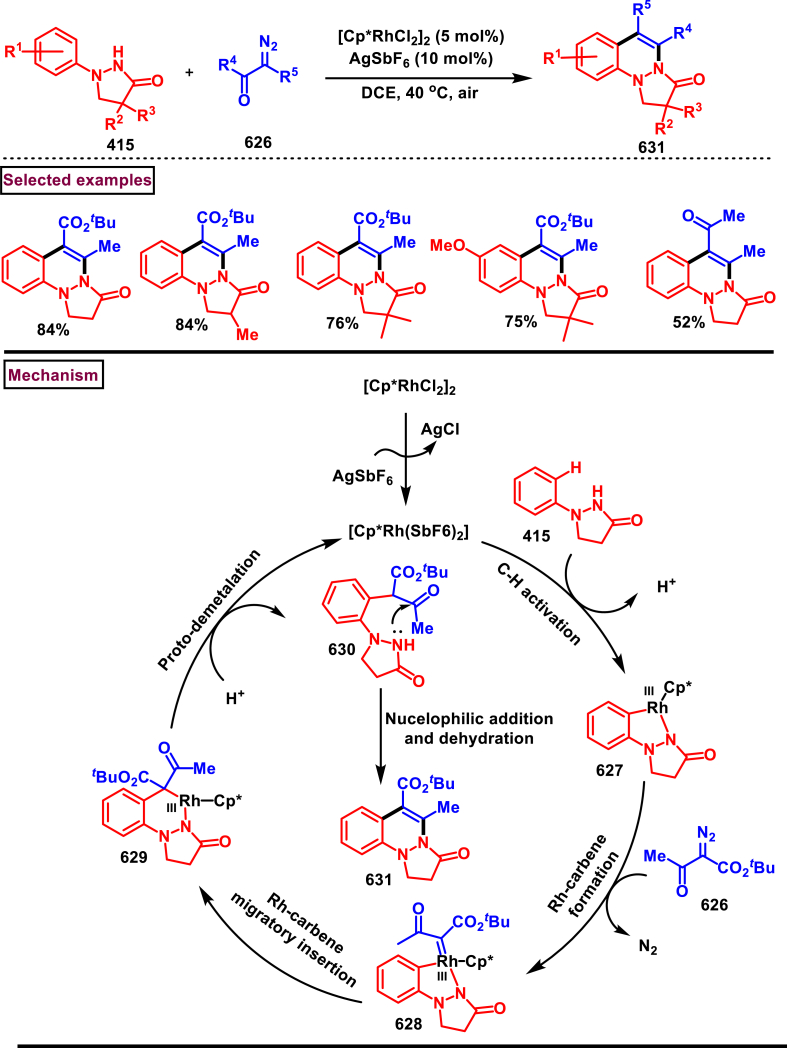
Synthesis of pyrazolo[1,2-*a*]cinnolines *via* Rh(III)-catalysed annulation of diazo compounds and pyrazolidinones.

Rh(III)-catalysed annulation of diazo compounds **(626)** and pyrazolidinones **(415)** to access medicinally important 5,6-disubstituted pyrazolo[1,2-*a*]cinnolines **(631)** was developed by Lin and Yao et al. (2018) (Scheme 112) [151]. This process was proceeded under redox-neutral conditions and pyrazolidinone **(415)** was served as a directing group. Structural variations at the aryl ring of pyrazolidinones was carried out with diverse functional groups. It was observed that, *meta*-substituted pyrazolidinones **(415)** afforded the regioselective products *via* the C-H activation on less hindered side. Diazo compounds **(626)** with various ester substitutions, ketones were furnished the desired products **(631)** in moderate to good yields (45%–80%). Interestingly, cyclic diazo compounds were also successfully involved in this transformation and provided the desired products in 54%–68 % yields. Environmentally benign N_2_ and H_2_O were released as by-products. Generation of five membered rhodium intermediate **(627)** was realized *via* the coordination of active rhodium catalyst between aryl C-H and N atom of pyrazolidinone **(415)**. Interaction of **(627)** with diazo compound **(626)** delivered the Rh-carbene intermediate **(628)** with the release of N_2_. Further migratory insertion and proto-demetallation process offered the ketone **(629)**. Intramolecular cyclization and dehydration furnished the product **(631)**.

#### 3.8.9 Pyranopyrazoles

Pyranopyrazoles are important sub-class of fused pyrazole family with diverse biological profile [152]. Among four isomeric forms, pyrano[2,3-*c*]pyrazoles are intensively studied due to their promising biological activities [153]. The structural features of this building allowed numerous modifications and allowed extensive structure-activity relationship studies. Herein, we compiled few interesting synthetic strategies for the synthesis of pyrano[2,3-*c*]pyrazole cores in recent years.

**Scheme 113:**
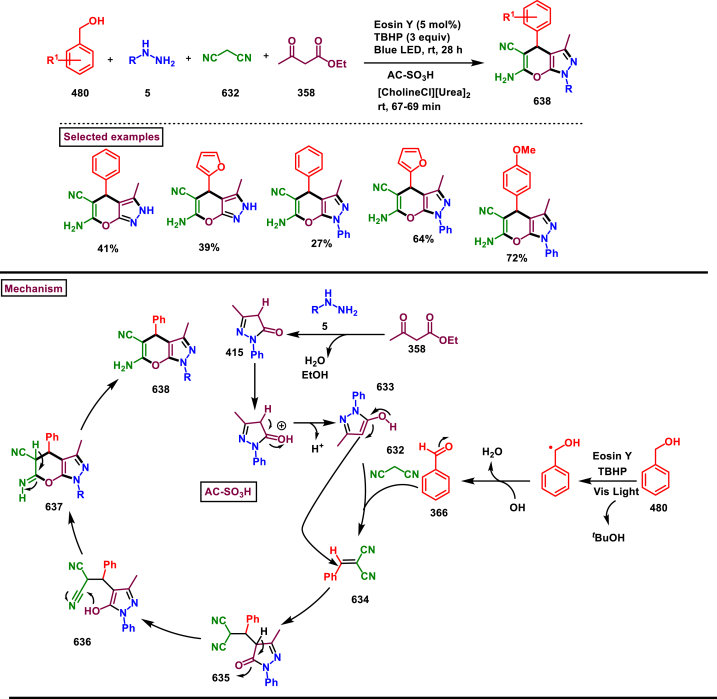
Synthesis of pyrano[2,3-*c*]pyrazoles under photocatalytic conditions.

Tran and Nguyen et al. (2022) combinedly reported a multicomponent strategy to access pyrano[2,3-*c*]pyrazoles **(638)** from phenylhydrazine **(5)**, ethyl acetoacetate **(358)**, malononitrile **(632)** and benzyl alcohol **(480)** in the presence of activated-carbon sulfonic acid (AC-SO_3_H) and Eosin Y catalysts (Scheme 113) [154]. Mechanistically, oxidation of benzyl alcohol **(480)** into benzaldehyde **(366)** was realized under visible light in presence of TBHP and Eosin Y. Further condensation with active methylene compound **(634)** and reaction with pyrazolone intermediate **(633)** (Formed from the direct reaction of ethyl acetoacetate **(358)** and phenylhydrazine **(5)** in the presence of AC-SO_3_H) resulted in the intermediate **(635)**. Intramolecular cyclization and imino-amino tautomerization resulted in the formation of pyrano[2,3-*c*]pyrazole derivatives **(638)**. Wide range of electron-donating groups and withdrawing substituents were tested on all the precursors. In most of the cases moderate to good yields were obtained.

**Scheme 114:**
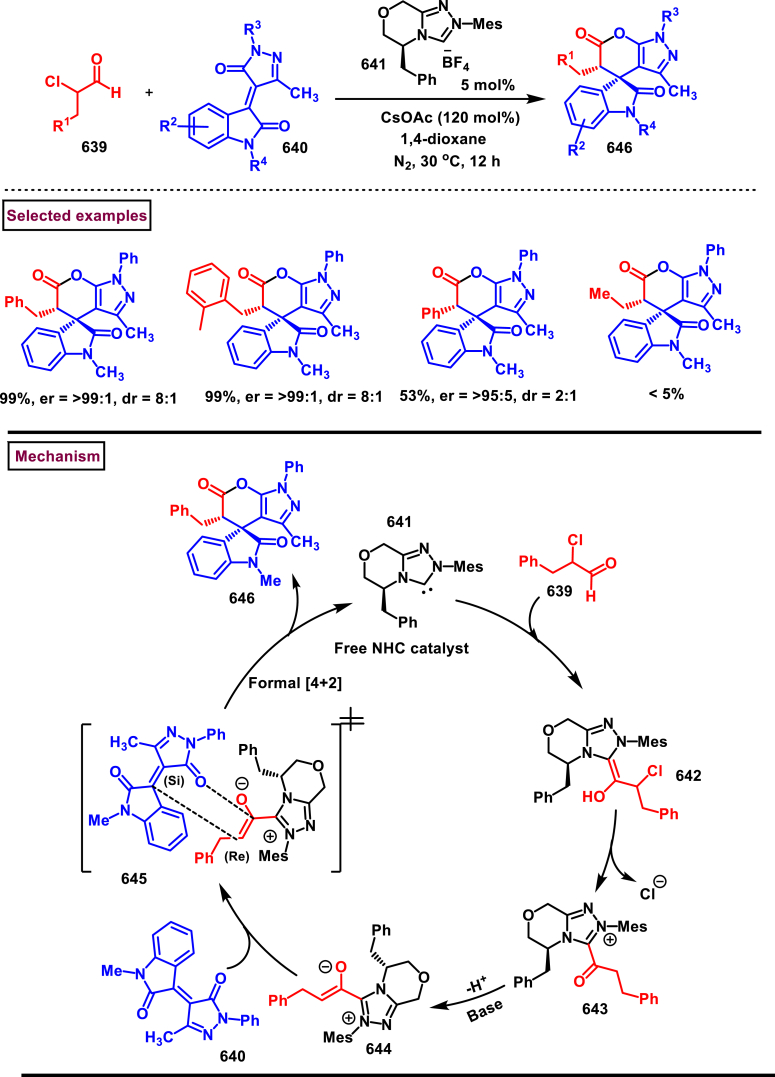
Synthesis of pyrano[2,3-*c*]pyrazoles *via* carbene catalysed cycloaddition.

A carbene **(641)** catalysed asymmetric [2 + 4] cycloaddition reaction of an aldehyde **(639)** and a pyrazolone **(640)** was developed to access structurally rich spirocyclic pyrano[2,3-*c*]pyrazoles **(646)** (Jin and Huang et al. 2022) (Scheme 114) [155]. Most of the chiral products obtained were structurally resembled the bioactive compounds and tested for their antibacterial effects. The tolerance of this protocol was studied towards various aldehyde **(639)** and oxindole-derived pyrazolones **(640)**. In most of the cases the good yields and excellent chiral purities are observed. Especially, *N*-alkyl, *N*-aryl, *N*-benzyl groups and free NH groups could be employed on the pyrazolones without much change in the yields and diastereoselectivity. Intermediate **(642)** was generated from the addition of NHC catalyst **(641)** to chloroaldehyde **(639)**. Acylazolium intermediate **(643)** was realized after losing chloride anion. Base mediated deprotonation and [4 + 2] cycloaddition with electrophilic **(640)** liberated the product **(646)**. It is noteworthy that, Re face of **(644)** and Si face of **(640)** were involved during key [4 + 2] cycloaddition step, thus products were obtained in enantioselective fashion. Readily available substrates, gram-scale synthesis, and mild reaction conditions are the features of this report.

**Scheme 115:**
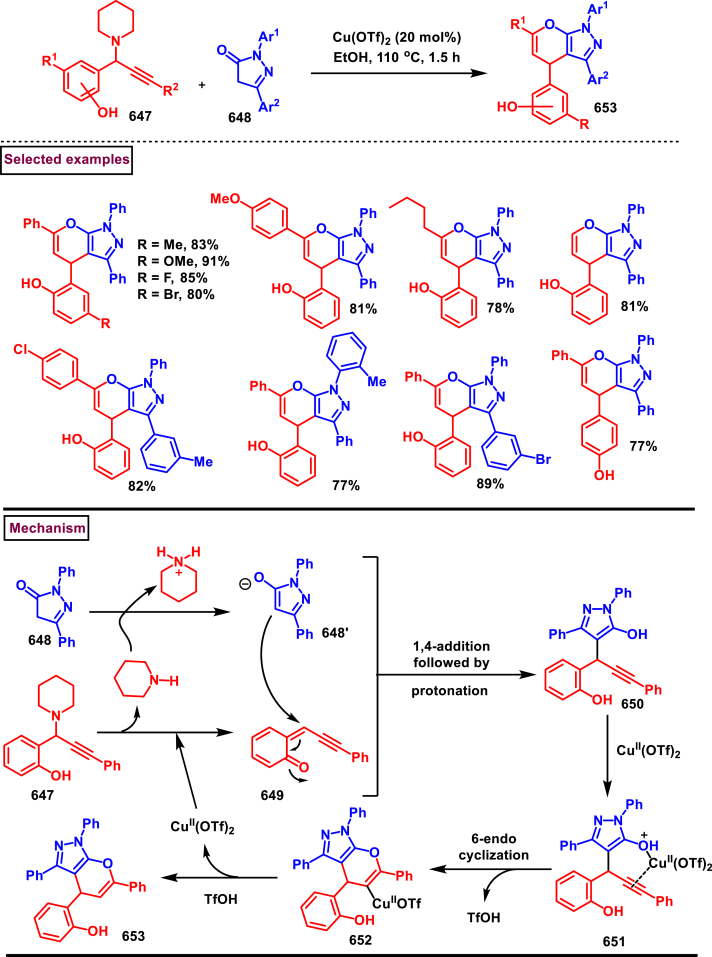
Cu-catalysed tandem synthesis of pyrano[2,3-*c*]pyrazoles.

In 2022, Wang and Zhao et al. described a Cu-catalysed cyclization of pyrazoline-5-ones **(648)** and *o*-hydroxyphenyl propargylamines **(647)** (*o*-HPPA) to prepare synthetically useful pyrano[2,3-*c*]pyrazoles **(653)** in moderate to good yields (Scheme 115) [156]. Facile construction of the desired products was realized with excellent functional group compatibility with respect to *o*-HPPA **(647)** and pyrazoline-5-ones **(648)**. Aryl group of phenolic moiety and alkyne units with different substituents furnished the desired products **(653)** in excellent yields. Notably, alkyl bearing alkynes and terminal alkynes both were evaluated to give pyrano[2,3-*c*]pyrazoles **(653)** in good yields. On the other hand, irrespective of the electronic bias and position several substituted pyrazoline-5-ones were smoothly engaged in this transformation. Mechanistically, alkynyl *ortho*-quinone methide **(649)** was formed from **(647)** *via* an elimination of piperidine which acted as base and produced pyrazolone enolate **(648′)**. 1,4-conjugate addition of **(648’)** and **(649)** generated the intermediate **(650)** which provided intermediate **(651)** *via* the coordination of Cu-catalyst to the alkyne unit. Selective 6-*endo* cyclization and protonation afforded the product **(653)**.

**Scheme 116:**
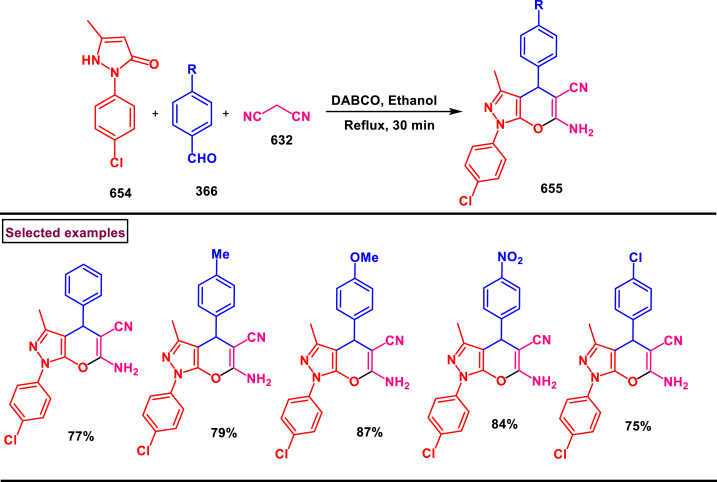
Multicomponent synthesis of *N*-aryl substituted pyrano[2,3-*c*]pyrazoles from pyrazolones, aryl aldehydes, and malononitrile.

A range of *N*-aryl substituted pyrano[2,3-*c*]pyrazoles **(655)** were prepared from pyrazolones **(654)**, aryl aldehydes **(366)**, and malononitrile **(632)** in presence of DABCO (Patel et al. 2022) (Scheme 116) [157]. Aryl aldehydes bearing electron donating groups (-Me, -OMe, -OEt, -OH) and electron withdrawing groups (-Cl, -Br, NO_2_, -F) were reacted smoothly to afford the desired products in moderate to good yields. Selected compounds of this category were tested for their *in vitro* anti-glioma activity.

**Scheme 117:**
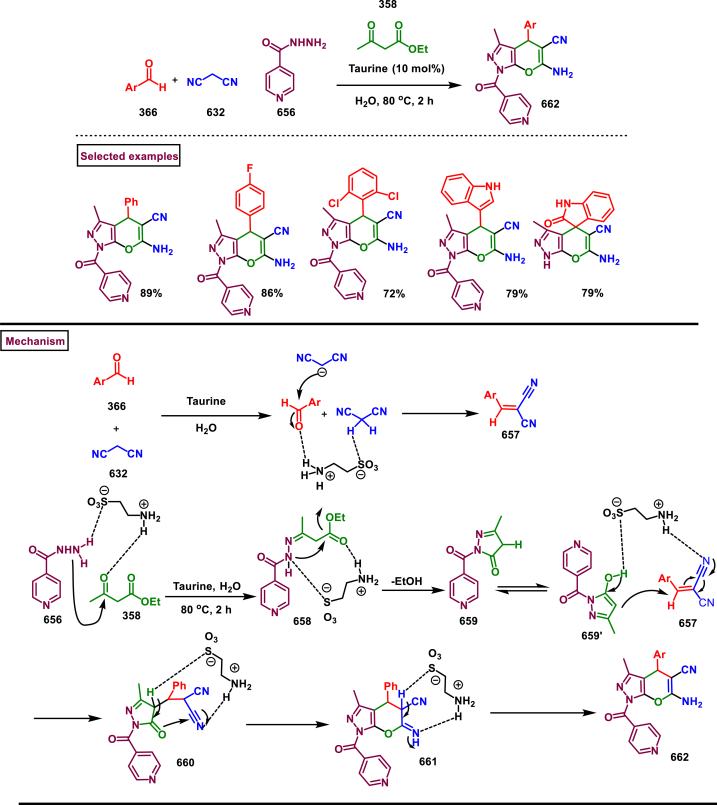
Taurine catalysed synthesis of pyrano[2,3-*c*]pyrazoles.

Preparation of a series of 1,4-dihydropyrano[2,3-*c*]pyrazoles **(662)** and spirooxindole 1,4- dihydropyrano[2,3-*c*]pyrazoles was disclosed *via* taurine catalysed multicomponent reaction system (Chate et al. 2021) (Scheme 117) [158]. This one-pot, four component method was well-tolerated the sensitive functional groups, electronically distinct substituents on the aryl ring of benzaldehydes, hetero arylaldehydes and polyaromatic aldehydes and the corresponding products **(662)** were obtained in excellent yields (72–99 %). By replacing the aryl aldehyde moiety with isatin, an innovative preparation of spiro-products was obtained in good yields. Taurin a natural β-amino sulfonic acid served as a bifunctional donor-acceptor activating agent. Mechanistically, taurine activated the ethyl acetate **(358)** which was attacked nucleophilically by hydrazine **(656)**. Resulting intermediate **(658)** underwent intramolecular cyclization to give pyrazolone **(659)**. On the other hand, adduct **(657)** was generated from the Knoevenagel condensation of taurine activated aryl aldehyde **(366)** and enolized malononitrile **(632)**. Intermolecular Michael addition of tautomerized **(659’)** to Knoevenagel adduct **(657)** provided the intermediate **(660)**. Subsequent intramolecular cyclization and imino-enamino tautomerization delivered the pyrano[2,3-*c*]pyrazole **(662)**.

**Scheme 118:**
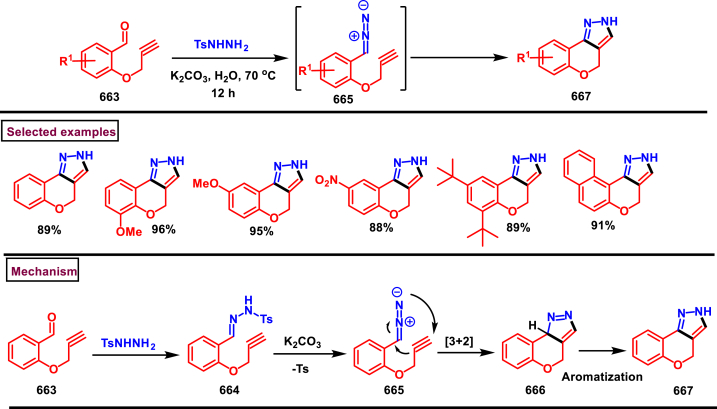
One-pot synthesis of benzopyranopyrazoles in water media.

In 2018,Muthusamy et al. disclosed an environmentally friendly one-pot, cascade approch towards benzopyranopyrazoles **(667)** in water media (Scheme 118) [159]. Salicylaldehyde based *in situ* generated alkynyl diazo substrate **(665)** readily reacted intramolecularly with unactivated alkenes or alkynes *via* [3 + 2] cycloaddition to yield the desired benzopyranopyrazoles **(667)**. By adapting a two-step procedure, diazo compounds **(665)** were prepared *in situ* from propargylated salicylaldehyde and tosylhydrazone under basic condition. Various substituents such as, -Cl, -Br, -NO_2_, -^*t*^Bu, -OEt on the aryl ring of salicyclaldehyde substrate were compatible and the desired products were isolated in moderte yields. Sterically challenging di-*tert*-butylbenzopyranopyrazole and naphthopyranopyrazole were also accessed in good yields. This method was also conveniently led pyrazoles incorporated into macrocyclic skeleton. Condensation of aldehyde **(663)** with tosylhydrazone was followed by a base mediated generation of diazo intermediate **(665)**. Intramolecular [3 + 2] cycloaddition and aromatization provided the benzopyranopyrazoles **(667)**.

#### 3.8.10 Pyrazolo [1,5-*a*] pyrimidines

Among different types of fused pyrazolo-pyrimidines, pyrazolo[1,5-*a*]pyrimidine skleton is frequently found in various biologically important molecules and markted drug products such as, Lorediplon (hypnotic), Indiplon (hypnotic), Zaleplon (hypnotic), and Ocinaplon (anxiolytic) [160]. Numerous synthetic approaches were developed in recent years to achieve this ubiquetous core [161]. Majority of the strtegies involve the reaction of aminopyrazoles with 1, 3-bis electrophiles such as β-enaminones, β-ketonitriles, or β-dicarbonyls). Herein, we compile a few very intresting synthetic methods that are aimed to synthezie pyrazolo[1,5-*a*]pyrimidine cores in recent years.

**Scheme 119:**
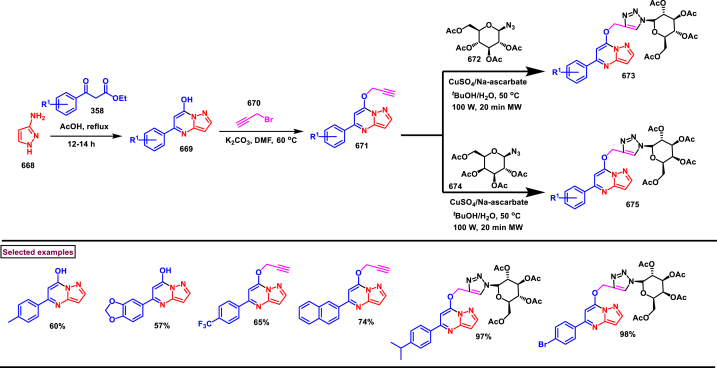
Microwave assisted synthesis of triazole tethered pyrazolo[1,5-*a*]pyrimidines.

Tiwari et al. (2024) prepared a series of 7-hydroxy pyrazolo[1,5-*a*]pyrimidines **(673** and **675)** by treating 3-aminopyrazoles **(668)** with β-ketoesters **(358)** in refluxing acetic acid. These products were propargylated **(671)** under basic conditions in excellent yields (Scheme 119) [162]. Under microwave irradiation, these propargylated products **(671)** were converted into triazole based glucohybrids **(673)** or galactohybrids **(675)** by treating with either azido glucosides **(672)** or azio galactosides **(674)** *via* Cu-catayzed Click reaction. Several numbers of sterochemically enriched glycohybrids possesing different functional groups were prepared. Above all, these pyrimidine based triazole skeletons were screened for their anticancer potential against MCF-7, MDA-MB453 and MDA-MB231 cell lines.

**Scheme 120:**
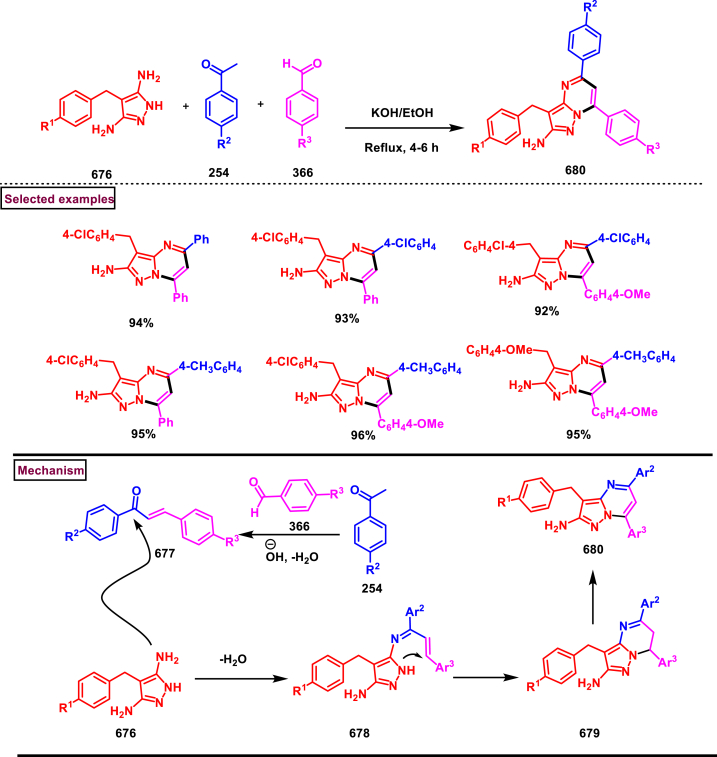
KOH mediated three component synthesis of pyrazolo[1,5-*a*]pyrimidines.

By reacting pyrazole-diamines **(676)**, aryl aldehydes **(366)** and aryl ketones **(254)** in a 1:1:1 ratio in presence of ethanolic KOH, a series of pyrazolo[1,5-*a*]pyrimidines **(680)** were obtained (Mekky et al. 2023) (Scheme 120) [163]. Pyrazole 3,5-diamines **(676)** were obtained by treating functionalized malononitriles with hydrazine following a literature reports. Most of the common functional groups and substituents were survived during this transformation and the desired products **(680)** were obtained in excellent yields (89–96 % isolated yields). According to the proposed mechansim, base mediated condensation of aryl ketone **(254)** and aldehyde **(366)** gave the enone **(677)** which underwent acylation with **(676)** to give **(678)**. Further intramolecular cyclization and dehydrogenation provided **(680)**.

**Scheme 121:**
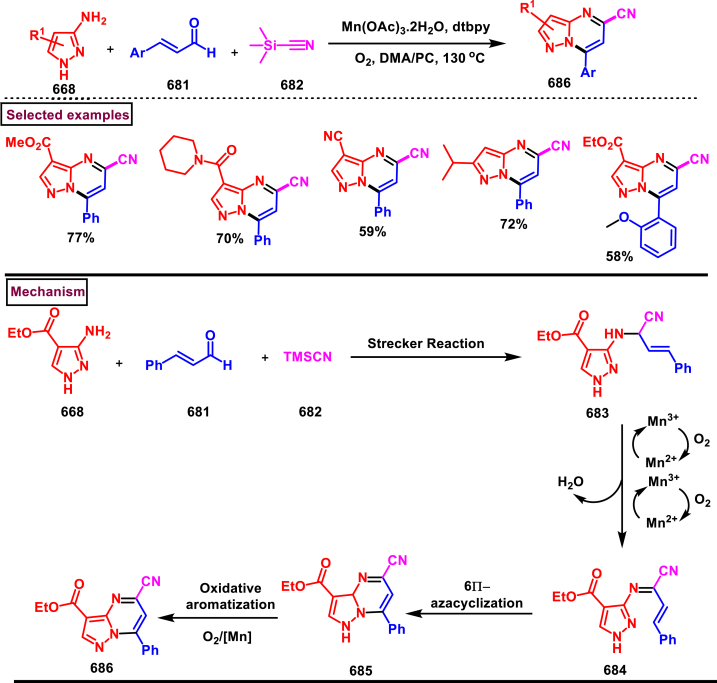
Mn(III)-catalysed synthesis of 5-cyano-pyrazolo[1,5-*a*]pyrimidines.

A step-economic Mn(III)-catalysed three component stragey for the preparation of 5-cyano-pyrazolo[1,5-*a*]pyrimidine **(686)** was developed from 3-aminopyrazoles **(668)**, aldehyde **(681)** and TMS-CN **(682)** by Xu and Zhang et al. (2023) (Scheme 121) [164]. Mechanistically, this transformation proceeded through a sequential Strecker reaction to give intermediate **(683)**. Further oxidative cyclization by using O_2_ gas as an oxidant and aromatization yielded the product **(686)**. Aryl and alkyl substituted 3-amino pyrazoles **(668)** were investigated and found that, presence of electron-withdrawing groups resulted in higher yields. Sensitive functional groups were also tollerated. In the case of α,β-unsaturated aldehydes **(681)**, steric effect was dominating as the *ortho*-functionalization led to lower yields. Control experiment supported the mechansitc proposal and salient merits of this report are, readily available precursors, mild reaction condition and less toxic cyanating agent.

**Scheme 122:**
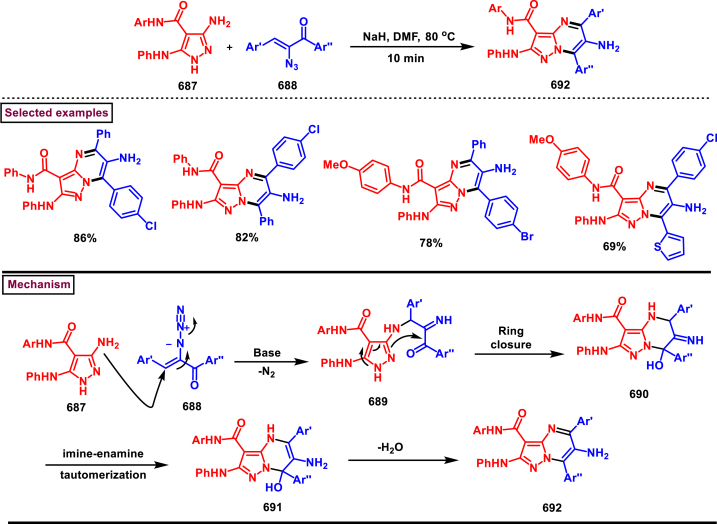
Synthesis of 6-amino-pyrazolo[1,5-*a*]pyrimidines from 3-aminopyrazoles and α-azidochalcones.

A base mediated synthesis of library of 6-amino-pyrazolo[1,5-*a*]pyrimidines **(692)** was realized from the readily available 3-aminopyrazoles **(687)** and α-azidochalcones **(688)** under mild conditions (Foroumadi et al. 2020) (Scheme 122) [165]. This reaction initiated *via* sequential intermolecular Michael-addition of **(687)** and **(688)** followed by an intramolecular cascade cyclization of **(689)** to give **(690)**. Sequential tautomerization and dehydration provided the product **(692)**. Common functionalities such as, Br, Cl, -CH_3_, -OMe were screened on both the reacting partners and the desired 6-amino-pyrazolo[1,5-*a*]pyrimidines **(692)** were obtained in excellent yields. Importantly, all these products were evaluated for their α-glucosidase inhibitory potency. IC_50_ values were observed in the range of 15.2 ± 0.4 μM to 201.3 ± 4.2 μM.

**Scheme 123:**
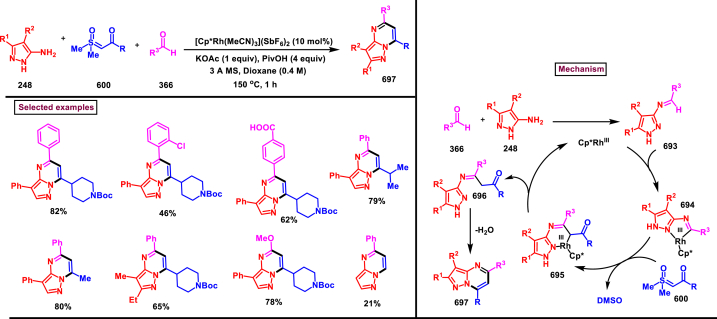
Rh(III)-catalysed synthesis of pyrazolo[1,5-*a*]pyrimidines using sulfoxonium ylides.

A set of synthetically intresting pyrazolo[1,5-*a*]pyrimidines **(697)** were obtained *via* a Rh(III)-catalysed, microwave assisted annulation reaction of aldehydes **(366)**, 5-aminopyrazoles **(248)**, and sulfoxonium ylides **(600)** (Ellman et al. 2018) (Scheme 123) [166]. Wide range of substituted aminopyrazoles **(248)** and aryl aldehydes **(366)** with electronically distinct functional groups smoothly parcipated under the reported conditions. Alkyl, aryl and heteroaryl units were successfully incorporated into the pyrazolo[1,5-*a*]pyrimidine skeleton by employing appropriate aldehydes. On the other hand, number of substituted sulfoxonium ylides **(600)** including linear, branched alkyl, aryl and heteroaryl units were also screened to afford the desired products in good to excellent yields. Regiospecific introduction of aryl units was achieved irrespective of the electronic properties. Moreover, for the first time, author employed formyl sulfoxonium ylides to prepare mono substituted pyrazolo[1,5-*a*]pyrimidines **(697)**.

**Scheme 124:**
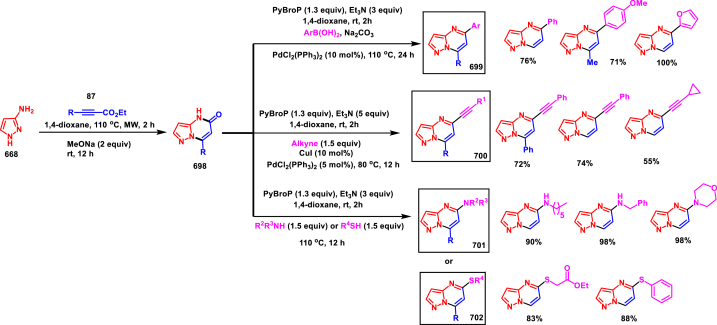
Synthesis of 5,7-disubstituted pyrazolo[1,5-*a*]pyrimidines *via* PyBroP mediated coupling reaction.

A consise preparation of 7-functionalized pyrazolo[1,5-*a*]pyrimidin-5-ones (**698**) was realized *via* a condensation of alkynyl ester (activated alkynes, **87**) and 3-aminopyrazoles **(668)**. This intermediate was used as a building block to synthesize a library of 5,7-disubstituted pyrazolo[1,5-*a*]pyrimidines **(699**–**702)** by using PyBroP as the activating agent (Abarbri et al. 2017) (Scheme 124) [167]. A series of alkynyl, amino, thiol, aryl groups were coupled at the C5-position in excellent yields. pyrazolo[1,5-*a*]pyrimidin-5-ones **(698)** was obtained in good regioselectivity *via* Michael addition followed by a lactam formation. 5-arylated pyrazolo[1.5-*a*]pyrimidines **(699)** were obtained through initial phosphonium coupling followed by a Suzuki-Miyaura cross-coupling. Variety of substituents on the arylboronic acid excerted good functional group tolerance. C-5 alkynation was realized through Cu-catalysed Sonagashira coupling **(700)** and the introduction of amino **(701)** or thiol group **(702)** was achieved by a SN_Ar_ reaction.

**Scheme 125:**
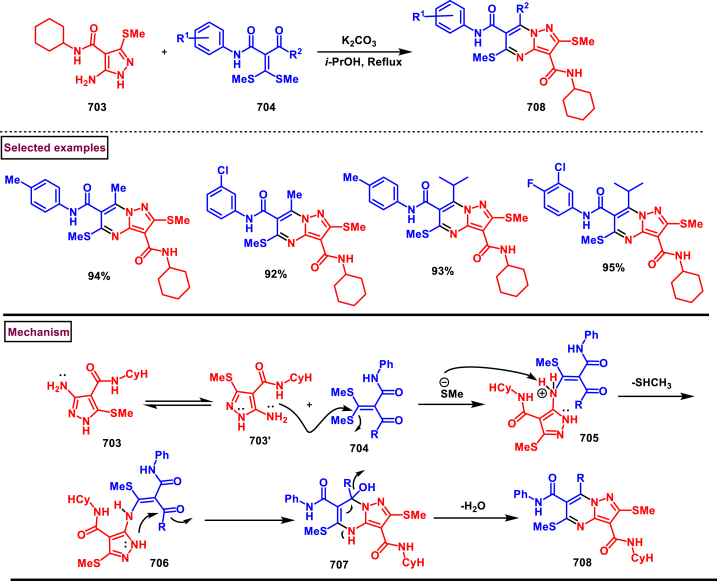
Synthesis of pyrazolo[1,5-*a*]pyrimidines *via* K_2_CO_3_ mediated [3 + 3] heteroaromatization.

Patel and Naliapara et al. (2017) combindely reported a novel method to prepare dicarboxamide bearing pyrazolo[1,5-*a*]pyrimidines **(708)** *via* K_2_CO_3_ mediated [3 + 3] heteroaromatization of 5-amino-*N*-cyclohexyl-3-(methylthio)-1*H*-pyrazole-4-carboxamide **(703)** and oxoketene dithioacetals **(704)** (Scheme 125) [168]. Both of these intermediates were prepared by literature procedures. A library of pyrazolo[1,5-*a*]pyrimidines **(708)** were prepared in refluxing *i*-PrOH and the corresponding products were obtained in 77–95 % yields. Varities of substituents on both the substrates were found well-tollerated. Mechanistically, this tranformation was initiated by the nucleophilic amino attack of aminopyrazole **(703’)** on the alkene of oxoketene intermediate **(704)**. Further intramolecular cyclization **(707)** and dehydrative aromatization provided the desired product **(708).**

**Scheme 126:**
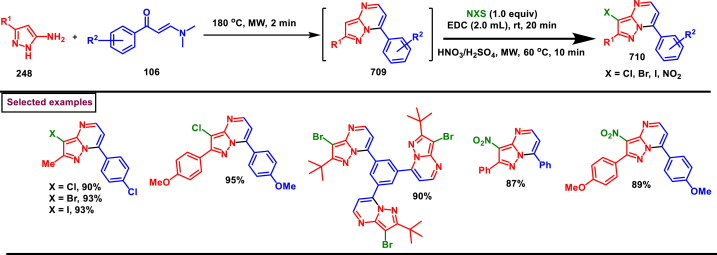
Microwave assisted synthesis of pyrazolo[1,5-*a*]pyrimidines from the condensation of 5-aminopyrazoles and β-enaminones.

Microwave assisted synthesis of 3-halo and 3-nitro pyrazolo[1,5-*a*]pyrimidines **(710)** was reported from the condensation of 5-aminopyrazoles **(248)** and β-enaminones **(106)** under catalyst free conditions (Portilla et al. 2017) (Scheme 126) [169]. Mechanstically, this process proceeded through cyclo condensation between the substrates to give **(709)** followed by a highly regioselective substitution of electrophiles. In this way, 3-halopyrazolo[1,5-*a*]pyrimidines **(710)** were obtained by using either NBS or NIS in excellent yields (89%–96 %). On the other hand, nitration was achieved using the nitrating mixture (HNO_3_:H_2_SO_4_, 2:1). Pd-catalysed Sonogashira coupling of halogenated products with acetylyne reagent and reduction of -NO_2_ unit into amino group were performed as a postsynthetic modification to prove the synthetic utility of this report.

## 4 Biological activities of pyrazole derivatives

**Fig. 4:**
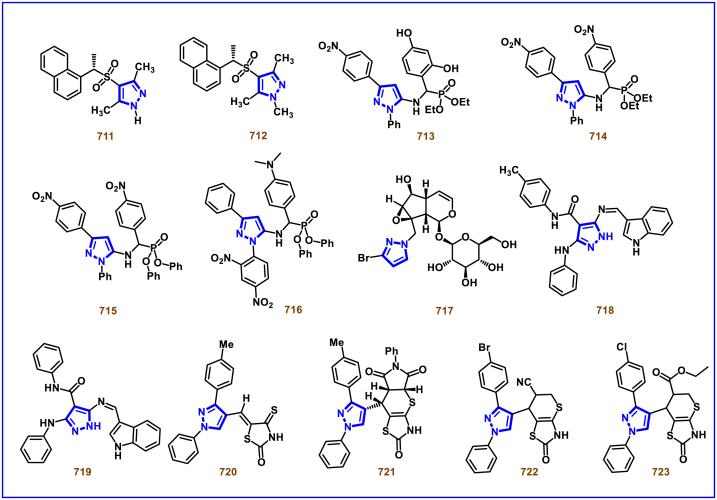
Pyrazoles with anti-cancer activities.

**Fig. 5:**
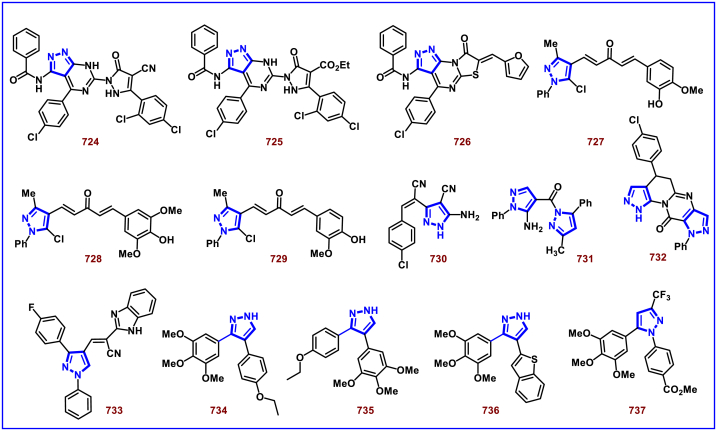
Pyrazoles with anti-cancer activities.

**Fig. 6:**
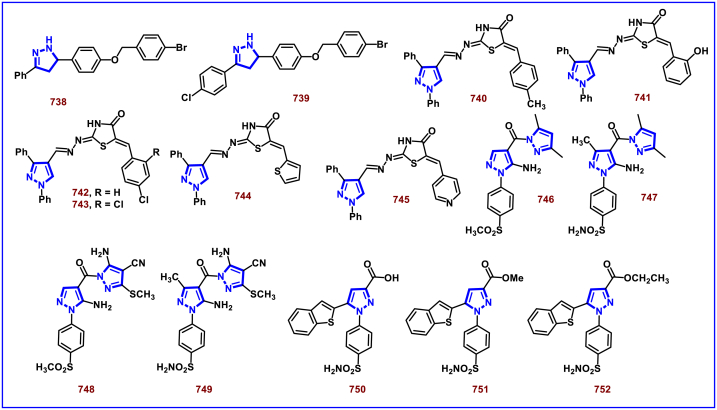
Pyrazoles with anti-inflammatory activities.

**Fig. 7:**
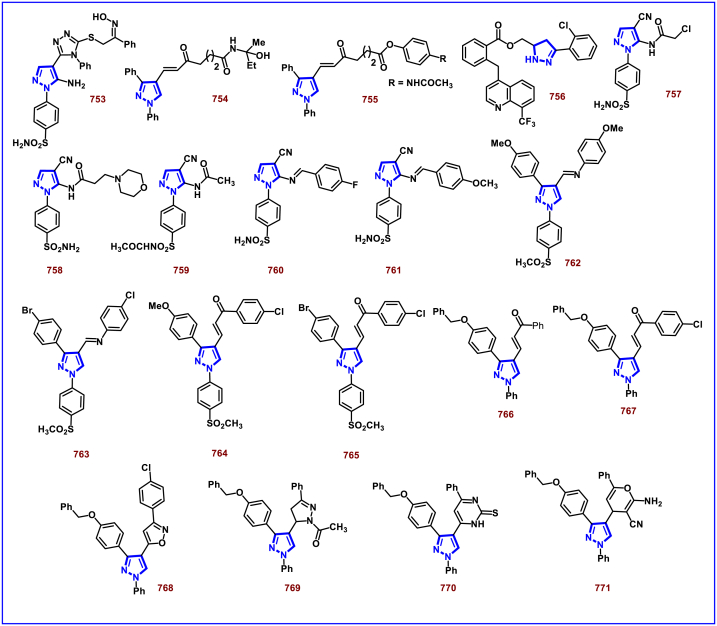
Pyrazoles with anti-inflammatory activities.

**Fig. 8:**
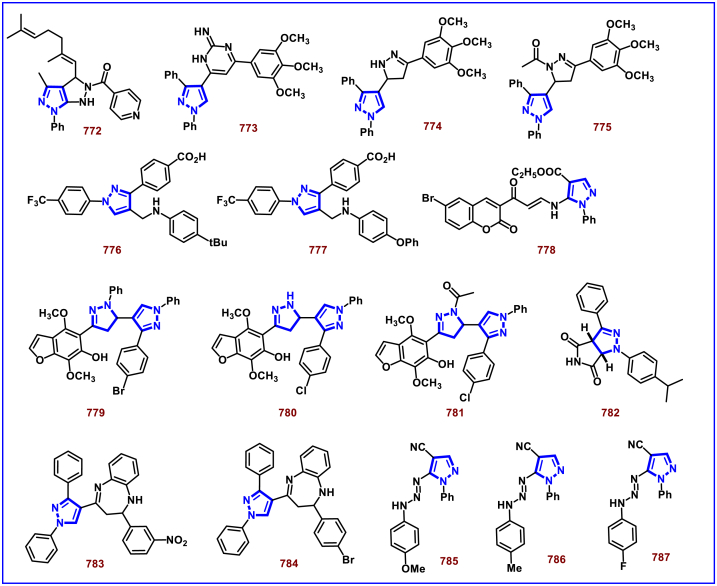
Pyrazoles with antibacterial activities.

**Fig. 9:**
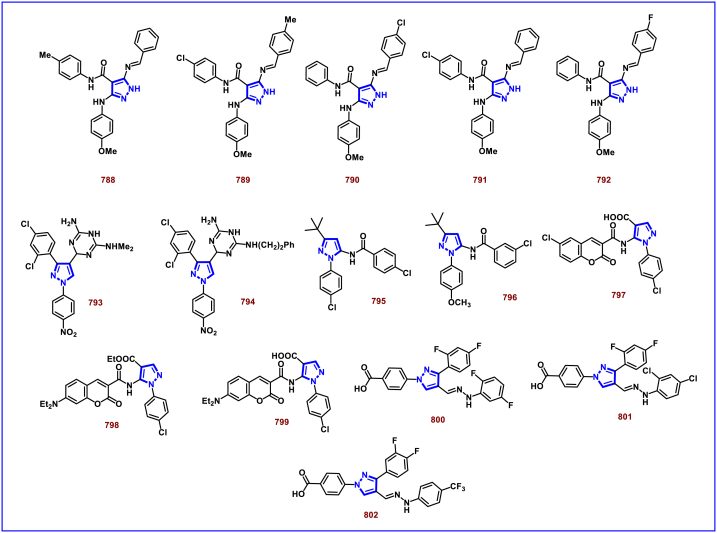
Pyrazoles with antibacterial activities.

**Fig. 10:**
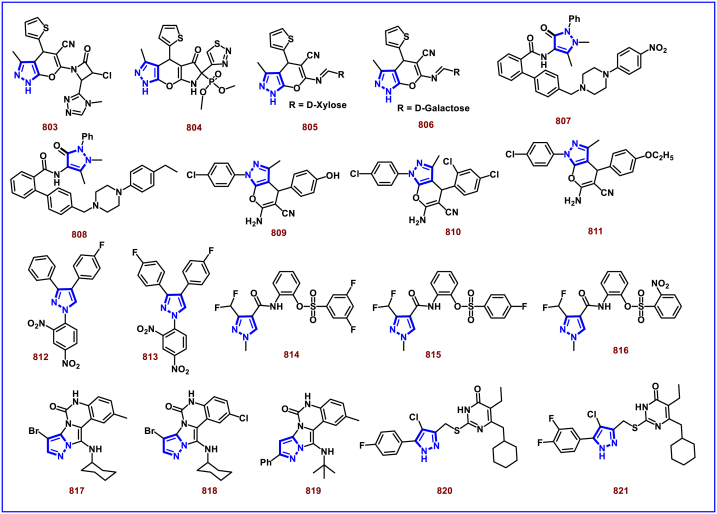
Pyrazoles with antiviral activities.

**Fig. 11:**
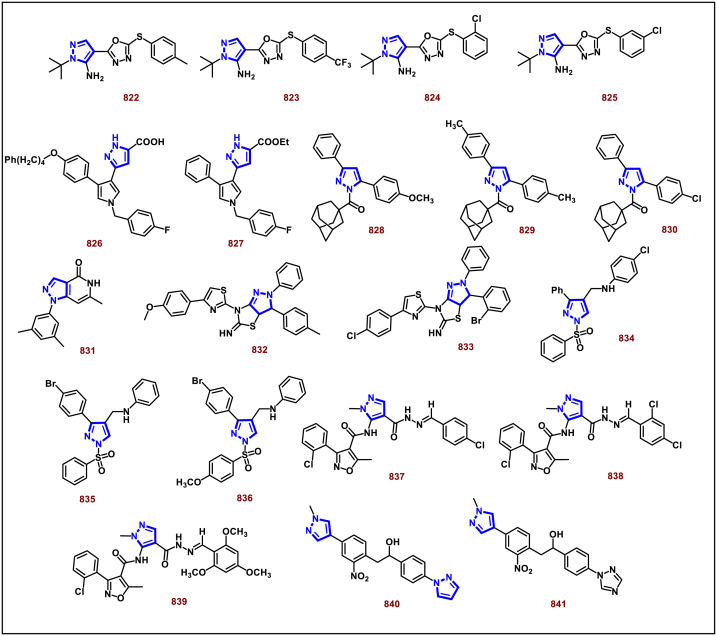
Pyrazoles with antiviral activities.

Pyrazoles are well-known for their broad-spectrum of biological activities including anti-cancer, antimicrobial, antiviral, anticonvulsant, antihypertensive, anti-HIV, antitubercular, anti-inflammatory, anti-fungal activities [[170], [171], [172], [173]]. Variety of pyrazole based commercially available drugs represent the privilege of this heterocyclic core [174,175]. In addition, a large number of pyrazole derivatives are used as NSAID drugs such as, Dipyrone or metamizole (pain killer and spasm reliver), Phenazone (anti-inflammatory), Phenylbutazone (prostaglandin inhibitor, analgesic), Ramifenazone, Lonazolac and Celecoxib [[176], [177], [178]]. In this context, we emphasize on the unique biological activities showcased by pyrazole moiety in recent years (from 2017 onwards, Fig. 4, Fig. 5, Fig. 6, Fig. 7, Fig. 8, Fig. 9, Fig. 10, Fig. 11).

### 4.1 Anticancer activity of pyrazoles

Gundla and Jonnalagadda et al. [179] synthesized a series of 19 pyrazole-4-sulfonamide derivatives and studied their *in vitro* anticancer activities (GI50) on human myeloid leukemia (U937) by the CellTiter-Glo assay. Inhibitory percentage was measured at various concentrations using Mitomycin-C as a standard. Compounds **711** and **712** showed distinct anti-proliferative activity among all the compounds prepared (Fig. 4). These compounds were possessing bulky and bicyclic aromatic hydrophobic units. The antiproliferative activity (GI50) was further studied by Graph Pad Prism analysis. In addition, cytotoxicity activity evaluation (CC_50_) was screened for these novel compounds on human myeloid leukemia (U937) cells. It was observed that, none of these derivatives showed noticeable cytotoxicity in LDH release assay even at high concentration (100 μM). Ibrahim and Ahmed et al. [180] evaluated the *in vitro* anti-cancer activities of novel α-aminophosphonates containing pyrazole moiety against Epdermoid carcinoma (HEP2), colorectal carcinoma (HCT-116) and human lung fibroblast normal cell lines (W138). It was observed that compounds **713, 714** and **715** exhibited excellent activity against colon carcinoma cells (HCT-116) with IC_50_ values of 0.7 ± 9.51, 1.6 ± 19.90, and 1.1 ± 15.06 μM compared with Doxorubicin standard **(**Fig. 4**).** Potential inhibitory activity was displayed for epdermoid carcinoma (HEP2) by compounds **713**, **715** and **716** with IC_50_ values 0.9 ± 12.5, 1.40 ± 17.76, and 1.8 ± 21.28 μM. As confirmed by the electrostatic potential calculations, the excellent interaction of both receptors with **713** was due to its high positive and negative surface potentials. Moreover, molecular docking studies revealed that the interaction of **713** with FGFR1 and VEGFR-2 proteins receptors was mainly hydrophobic interactions (π-cation, H-donor, H-acceptor, salt bridge and halogen bond interactions). In addition, these pyrazoles bearing α-aminophosphonates were found to have low cytotoxic activity on the WI-38 cells. Dong et al. [181] synthesized a series of pyrazole containing catalpol derivatives by modifying C10 hydroxyl group using drug combination principle. Anti-esophageal activity of these novel compounds was evaluated against two human esophageal cancer cell lines Eca-109 and EC-9706. Similarly, anti-pancreatic activity was studied against human pancreatic cancer cell lines PANC-1, BxPC-3 and HPDE6-C7 *via* MTT assay. Results of anti-esophageal and anti-pancreatic cancer cell lines revealed that, the pyrazole structure and was crucial for the structure-activity relationship (SAR). Based on the protein specific docking score, interaction of compound **717** with target enzyme was studied. It was observed that, free energy of binding of **717** was −6.97 kcal/mol which was remarkably higher than catalpol (−4.5 kcal/mol) proving these compounds have superior target-enzyme interaction. Further analysis revealed that, the compound **717** formed hydrophobic interaction with the amino acid residues (LYS920 and CYS919) whereas, the target enzyme interaction was further increased by hydrogen bonding between the hydroxyl group of the catalpol unit and the LEU840/LYS838 amino acid residues. A molecular hybridization based protocol was developed to prepare a series of indole linked pyrazole derivatives and studied for their *in vitro* anti cancer activities against four human cancer cell lines such as human breast adenocarcinoma (MCF-7), human colorectal carcinoma (HCT-116), human lung carcinoma (A549) and human liver carcinoma (HepG2) using MTT assay [182]. All the prepared compounds were displayed good activities, especially **718** and **719** exhibited excellent inhibition against HepG2 cancer cell lines with IC_50_ values of 7.9 ± 1.9 μM and 6.1 ± 1.9 μM compared to the reference drug Doxorubicin (24.7 ± 3.2 μM). In addition, these compounds (**718** and **719**) demonstrated notable inhibition for cyclin-dependant kinase 2 (CDK-2) and MCF-7 cancer cell lines. To understand the binding modes of **718** and **719** with the amino acid residue in the active site of CDK-2, molecular docking studies were carried out. It was found that, **718** and **719** were well-fitted in the enzyme site using Lys89 *via* two arene-cation interactions with the indole NH and N2 of pyrazole moiety. It was also found that, another supporting H-bonding was provided by N1 of pyrazole with the side chain of Lys89. Additionally, both of these inhibitors displayed energy scores of −13.68 and −12.55 kcal/mol demonstrating high binding affinity. Flow cytometry and enzymatic assay studies were also carried out for these two potential anti-cancer compounds. Another molecular hybridization strategy was developed by Metwally et al. [183]. to achieve a differently functionalized series of pyrazole derivatives with thiopyrano[2,3-*d*]thiazoles. These compounds were tested for their *in vitro* anticancer activities against MCF-7 (human breast cancer) and HEPG-2 cancer cell lines (hepatocellular carcinoma) *via* MTT assay method using doxorubicin as a standard. Among the tested compounds **720** showed strong anticancer activity towards MCF-7 (IC_50_ 11.16 ± 1.5 μg mL^−1^). In another series, **721** displayed excellent activity against MCF-7 (IC_50_ 25.40 ± 2.1 μg mL^−1^) and HePG-2 cells (IC_50_ 21.31 ± 1.8 μg mL^−1^). In another series of cycloadducts, **722** and **723** were found to be more potent against MCF-7 (IC_50_ 10.10 ± 2.1 μg mL^−1^) and HePG-2 (IC_50_ 9.11 ± 3.2 μg mL^−1^). In comparison to standard acetazolamide (AZA), **722** efficiently inhibited CAIX (IC_50_ = 0.067 ± 0.003 μM) whereas, **723** selectively inhibited CAXII (IC_50_ = 0.123 ± 0.007 μM). This ability was owing to the electron withdrawing groups (-Br, -Cl) present in their structure. Molecular docking studies-based interaction pattern of potent compounds and their isomers revealed that the carbonyl group on the thiazole unit is crucial for the interaction with the active site of 5U0F.

Recently, a novel series of pyrazolo[3,4-*d*]pyrimidines were reported by Hassaballah et al. [184] and evaluated for their *in vitro* anti-proliferative efficacy against NCI 60 cancer cell lines. Pyrazolo[3,4-*d*]pyrimidines **724** and **725** displayed excellent cytotoxic activity towards full 60-cell panel (GI50 0.018–9.98 μM) (Fig. 5). Notably, cyano pyrazole derivative **724** showed more than 90 % cell growth inhibition against leukemia, colon (KM12), prostate and NCI-H460 cell lines. It also exhibited 100 % inhibition against ovarian, renal, melanoma and breast cancer cell lines. Moreover, candidates **724**, **725** and **726** were exhibited strong EGFR tyrosine kinase inhibition with the IC_50_ 0.135 μM, 0.034 μM and 0.054 μM respectively. In silico molecular docking studies of these active compounds revealed that, the pyrimidine unit was oriented into the adenine pocket *via* hydrophobic interaction with Leu718 and Gly796 amino acid residues. Hydrogen bonding interaction was observed with Met793. In 2022, Doan and Troung et al. prepared a series of 1-aryl-1*H*-pyrazole-fused curcumin analogues and tested for their *in vitro* cytotoxicity towards breast cancer cell lines MDA-MB-231 and liver cancer cell lines HepG2 *via* MTT assay method [185]. Many of the prepared compounds shown excellent growth inhibition towards HepG2 (IC_50_ 4.98–14.65 μM) and MDA-MB-231 (IC_50_ 2.43–7.84 μM). Particularly, **727**, **728**, and **729** were acted as effective microtubule destabilizing agents at 20.0 μM concentration. Additionally, cell cycle analysis of **727**, **728**, and **729** arrested MDA-MB-231 cells in G_2_/M phase at lower concentrations. Molecular docking studies proved that, **728** could efficiently bind at the colchicine-binding site (CBS). The binding ability of **728** with tubulin was studied by molecular docking studies showed that, **728** attained increased stability *via* various hydrophobic and hydrogen bonding interactions with significant binding energy (−10.08 kcal/mol. A series of fused imidazopyrazoles, pyrazolopyrazolopyrido-pyrimidines, pyrazolopyridines were reported by Aziem et al. [186] and screened against various human cancer cell lines using Adriamycin as a positive control. It was observed that, compound **730** showcased high anticancer activity against renal cancer cell line UO-31 (GI, 42.81 %). Compound **731** was exhibited good activity against breast cancer cell line MCF7 (GI, 49.88 %) and T-47D cells (GI, 38.15 %). Product **732** (Fig. 5) resulted excellent activity towards Leukemia CCRF-CEM (GI, 41.37 %) and SR (GI, 44.95 %). A novel class of heterocyclic framework possessing benzimidazole and pyrazole units were synthesized following a multi-step synthetic route [187]. Anticancer evaluation of these compounds *via* CellTiter-Glo luminescent assay method showed that *p*-fluorophenyl possessing hybrid product **733** exhibited superior activity against the human pancreatic cancer cell lines SW1990 (Squamous) and AsPCl (Progenitor). Remarkable binding affinity (−8.65 kcal/mol) was measured between B-cell lymphoma and the conformationally stabilized **733**. According to the docking studies, it was observed that **733** exhibited van der Waals interaction with various amino acids such as TYR 105, LEU 134, GLU 149, ASP 108, GLY 142 and PHE 101. In addition, a hydrogen bonding interaction between imidazole NH and ALA 146 was observed. Moreover, A donor-donor interaction between aryl ring of pyrazole and ARG 143 was also seen. Besides some of the prepared hybrid entities were exhibited high anti-inflammatory activity superior to standard diclofenac sodium. In 2019, Romagnoli et al. reported a series of Combretastatin A-4 analogues of 3-(3′, 4′, 5′-trimethoxyphenyl)-4-substituted-1*H*-pyrazole and its regioisomeric 3-aryl-4-(3′,4′,5′-trimethoxyphenyl)-1*H*-pyrazole derivatives [188]. These compounds were tested for their *in vitro* anticancer against various human cancer cell lines. Inhibition effects on tubulin polymerization was also studied for the selected active compounds. Among the tested compounds, **734** possessing 4′-ethoxyphenyl group at the C-4 position of pyrazole ring and its isomer **735** exhibited pronounced anticancer activity with IC_50_ values of 0.05–4.5 nM and 0.06–0.7 nM. Cytotoxicity effect of these compounds against peripheral blood lymphocytes and normal human astrocytes was observed in the range of GI_50_ > 10 μM indicating their non-toxic nature. These compounds were tested for their tubulin polymerization inhibitory effects. Compound **736** was found to be very active (IC_50_, 0.30 μM) and it was twice compared to CA-4. In addition, **736** was a potent colchicine binding inhibitor. Interaction of the active compounds with the colchicine site of tubulin was studied using molecular docking experiments. It was found that, trimethoxyphenyl ring was immersed into the hydrophobic pocket created by various amino acid residues and involved in a series of hydrophobic and hydrogen bonding interactions. Binding modes of selected compounds were further studied by molecular dynamic simulations and the relative binding energies (ΔG_binding_) were calculated. In 2018, Panda et al. reported a series of Combretastatin-(trifluoromethyl)pyrazole analogues [189]. These compounds were having the structural features of commercially available Celecoxib and Combretastatin. Anti-proliferative effect of these analogues was studied against various cancer cell lines. It was found that, compound **737** strongly inhibited the proliferation of B16F10 (mouse skin cancer, IC_50_ 6.0 ± 0.6 μM), MCF-7 (IC_50_ 1.3 ± 0.8 μM), EMT6/AR1 (MDR-mouse mammary cancer, IC_50_ 14.7 ± 0.3 μM) and HeLa (human cervical cancer, IC_50_ 5.5 ± 0.6 μM). Importantly, **737** showed lower toxicity against non-cancerous cells such as, Epithelial human breast cells (MCF10A) and fibroblast mouse skin cells (L929). IC_50_ of 23.2 ± 0.8 μM was determined for 737 against MCF10A which was 18 times more than the value of MCF-7 cancer lines. On the other hand, 14 ± 1 μM was obtained against L929 which was 2 times more than that of B16F10 cancer lines. In addition, **737** involved in the depolymerization of interphase microtubules, disruption of mitotic spindle formation and arrested MCF-7 cell lines at mitosis, eventually led to cell death. The binding energy of **737** with tubulin was estimated as −8.5 kcal/mol indicating **737** binds to tubulin with stronger affinity. Hydrogen bonding interactions were observed in the binding pocket of docked **737**.

### 4.2 Anti-inflammatory activity of pyrazoles

In 2021, Hadjipavlou-Litina et al. synthesized a series of pyrazoles and pyrazoline derivatives *via* condensation method and evaluated for their antioxidant, lipoxygenase inhibition (*in vitro*) and *in vivo* anti-inflammatory activity *via* the carrageenin-induced rat paw edema assay method [190]. Among all the prepared compounds, pyrazoline derivatives **738** and **739** were found to be most lipophilic and exhibited excellent anti-inflammatory activity (Fig. 6). In particular, **738** was found to be effective agent for both acute and chronic arthritis inflammation and the inhibition showed was higher than indomethacin standard (66.1 % Writhing inhibition). In addition, **738** was also investigated for anti-adjuvant-induced disease (AID). Additionally, **738** and **739** were found more lipophilic which was crucial for the enhanced anti-inflammatory activity. Molecular docking studies revealed the preferred docking poses of **738** and **739** which are bound to LOX-1 enzyme. Pyrazole-thiazolidinone derivatives were prepared and investigated *in vivo* for their anti-inflammatory activities using carrageenan induced rat paw edema method with the reference drugs Celecoxib, Indomethacin, diclofenac by El-Karim et al. [191]. Biological data revealed that, compounds **740**, **741**, **742**, **743**, **744**, and **745** were the most efficient candidates showing high potency at the first hour of administration and long post-administration action (up to 4h). Notably, compared to the reference drugs, these compounds were displayed higher GIT safety profile. In addition, these selected candidates were screened for their ulcerogenic effects, analgesic properties and *in vitro* TNF-α inhibitory effects. Structure-activity relationship (SAR) studies demonstrated that the substitutions on benzylidene unit showed up remarkable influence on the anti-inflammatory potency. Docking of these promising compounds into the active site of TNF-α revealed that, the compounds were well fitted into the binding pocket *via* an arene-arene interaction of pyrazole unit with the **Tyr119** amino acid residue. Superimposition model further concluded that, pyrazole skeleton was responsible for the excellent binding affinity through arene-arene interaction. Novel pyrazole scaffolds bearing amino and methane sulphonyl groups were prepared and screened for their *in vivo* anti-inflammatory effects using carrageenan induced rat paw edema method (Abdellatif et al.) [192]. Compounds **746**, **747**, **748**, and **749** showed best edema inhibition percentage (EIP = 78.9–96 %) than the reference drug Celecoxib (82.8 %) (Fig. 6). In addition, *in vitro* investigation of COX-I and II inhibitory properties revealed that, these selected compounds were possessing superior COX-II inhibitory effects with IC_50_ ranging from 0.034 to 0.052 μM). Molecular docking studies were performed to identify the possible binding patterns of these promising compounds with COX-II receptor. All compounds were found well-fitted in the active pocket with the binding energies from −12.52 to −15.00 kcal/mol. Presence of SO_2_ as a pharmacophoric unit was necessary owing to its H-bond interactions with amino acid residues such as, Arg499, Gln178 and Phe504. A novel series of pyrazole sulfonamide derivatives were prepared and tested for their *in vivo* anti-inflammatory property using carrageenan-induced rat paw edema model utilizing Celecoxib and Indomethacin reference standards (Kassab et al.) [193] Based on the biological data, it was found that carboxylic acid **750** and its ester analogue **751** and **752** exhibited excellent anti-inflammatory effect of 86 %, 78 % and 66 % protection when compared to the reference standard indomethacin (66 %). These compounds displayed rapid action from the time of administration to the 4th hour. In addition, these compounds displayed superior analgesic activity, ulcerogenic effects, *in vitro* COX-I, COX-II and 5-LOX enzyme inhibitory activities. According to docking experiments, it was observed that the COX-II inhibitors shown more negative score of −15.37 kcal/mol compared to COX-I (−12.60 kcal/mol) indicating the selective binding of COX-II. Moreover, promising candidates 750–752 possessed required structural features for the efficient binding with target enzymes. Accordingly, the pyrazole and aryl rings were interacted with active site through hydrophobic interactions whereas, carboxylic acid moiety had an ionic interaction towards various amino acid residues.

A new set of hybrid pyrazole derivatives containing 1,2,4-triazole and oxime unit were synthesized and screened for *in vivo* anti-inflammatory activity using carrageenan-induced rat paw edema model (Abdellatif et al.) [194] All of these compounds exhibited moderate activity (25.53–70.21 %) compared to Celecoxib standard. Similarly, *in vitro* analysis showed that sulphamoyl derivative **753** was the potent candidate (Fig. 7). Few of the prepared compounds displayed higher potencies (ED_50_ = 46.98–54.45 μmol/kg). Moreover, *in vitro* isozyme inhibitory studies revealed that these compounds showed high inhibition against COX-II isozyme (IC_50_ = 0.55–1.88 μM range) with highest COX-II selectivity index. Docking studies on the active binding sites of COX-II and EGFR revealed that, phenyloxime moiety of **753** is bound to the receptor with the oxime unit forming two hydrogen bonds with Thr 830A amino acid residue. Elnagdi et al. reported a set of pyrazole amides, ester and pyrazoline derivatives and studied their anti-inflammatory effects at various time intervals using Indomethacin as a reference drug. Compounds **754**, **755**, **756** displayed remarkable activity with 30.95%–28.57 % of edema inhibition [195]. The length of the methylene units was crucial for the increased anti-inflammatory activity. Compared to the free carboxylic acid group, its analogues amide or ester derivatives were found to be more potent owing to the reduction of acidic nature. Interaction of active compounds with the binding site of COX-II was studied by docking experiments. Among the tested compounds, **754** shown highest binding affinity with the target enzyme with the binding energy of −16.390 kcal/mol. It acted as a H-bond acceptor *via* carbonyl moiety with Arg 120 amino acid residue. In addition, the pyrazole ring of the **754** displayed π-H interaction with Val349 and Ala527 amino acid residues. Refaey et al. assessed a new set of pyrazole derivatives and investigated their *in vitro* COX-I and COX-II inhibitory activities [196]. Compounds **757**, **758**, **759**, **760** and **761** displayed excellent inhibition towards COX-II enzyme with IC_50_ values of 19.87, 39.43, 61.24, 38.73, 39.14 nM (Fig. 7). These selected compounds were subjected further to *in vivo* screening of their anti-inflammatory effect *via* carrageenan-induced rat paw edema assay method. Compound **757** exhibited excellent activity with an edema inhibitory percentage of 46.05 after 6 h of administration. Other compounds were displayed inhibition in the range of 42.96–44.33 %. Compounds **758**, **759**, **760**, **761** were docked into the active binding site of COX-II enzyme receptor. Except **759**, all other compounds formed H-bonds through sulphonamide oxygens, amino group, carbonyl oxygen and -CN group with various amino acid residues. On the other hand, **759** formed hydrogen bonds with -CN group and Arg 120 followed by another H-bond between sulphonamide oxygen and Ser 530 amino acid residue. A new series of pyrazoles containing Shiff base and chalcones were prepared and screened for their *in vitro*/*in vivo* anti-inflammatory activities by Amin et al. [197]. All the synthesized compounds were displayed reasonable edema inhibition (13–93 % and 58–93 % respectively). At three different time intervals, compounds **762**, **763**, **764** and **765** were shown excellent anti-inflammatory effects (ED_50_ = 89.2, 80.4, 72.8, 65.6 μmol/kg) in comparison with reference drug Celecoxib. Compound **764** was found to be the most selective COX-II inhibitor (IC_50_ = 0.068 μM). Mode of binding for these representative compounds was studied by docking them into the active binding sites of COX-II and COX-I enzymes. Compound **764** displayed the best binding free energy for COX-II (ΔG_*b*_ = −10.45 kcal/mol). Importantly, these compounds exerted four H-bonds with the amino acid residues of COX-II active site. In addition, these selected compounds were also investigated for their ulcerogenic properties. Through the structural alteration of Lonazolac drug (Non-selective NSAID associated by gastrointestinal problems), Harras and co-workers designed and evaluated the *in vivo* anti-inflammatory activity of novel 1,3,4-trisubstituted pyrazoles *via* carrageenan rat paw edema method [198]. *In vivo* anti-inflammatory activity of synthesized compounds was studied at five different time intervals and related to Celecoxib. It was observed that compounds **766**, **767**, **768**, **769**, **770**, and **771** were exhibited higher activities (42.2–89.7 %, 54.4–77.1 %, 41.8–69.9 %, 24.7–55.2 %, 15.5–57.7 %). These compounds were also studied for their ulcergenic activities. In addition, chalcone derivatives **766** and **767** displayed remarkable *in vitro* COX-II inhibitory activities together with *in vivo* anti-inflammatory activities. Docking studies described that, **766** and **767** were fitted well into the binding site of COX-II enzyme with the binding energies of −9.461 kcal/mol and −7.962 kcal/mol. Similar binding modes were observed for both the compounds. Two H-bonds were formed between the oxygen atom of chalcone carbonyl and side chains of Arg106 and Tyr341. Additional arene-H bonding was also observed between pyrazole and Ser339 residue. Interestingly, **766** and **767** were also docked with COX-I with a binding energy of −1.938 kcal/mol and −2.193 kcal/mol which was less compared to that of COX-II binding values.

### 4.3 Anti-bacterial activity of pyrazoles

Akbar et al. disclosed a set of pyrazolo[3,4-*c*]pyrazoles and studied their antibacterial activity against various Gram-positive bacteria (Enterococcus Faecalis, Staphylococcus aureus) and Gram-negative bacteria (*Escherichia Coli* and *Klebsiella pneumonia*) *via* disk diffusion method using Ciprofloxacin as a standard drug [199]. Antibacterial screening of these compounds revealed that **772** showed highest activity against Escherichia Coli (MIC: 1 μg mL^−1^) which was superior than the standard drug (Fig. 8). Binding affinity of **772** with 1AJ0 protein showed higher docking score (−3.7 kcal/mol) than standard drug (−5.6 kcal/mol). In addition, some of the compounds were displayed excellent anti-fungal and cytotoxic activities. Most of the studied compounds in this series displayed relatively low cytotoxicity compared to doxorubicin standard (LC_50_: 21.05 ± 0.82 μg mL^−1^) against MCF-7 cancer lines and normal Vero cells. *In vitro* and *In vivo* evaluation against Methicillin-Resistant *Staphylococcus aureus* and *Pseudomonas aeruginosa* were performed with a novel class of pyrazole embedded pyrimidine and pyrazoline compounds (Mansour et al.) [200]. Target compounds were studied *in vitro* against the strains *via* agar diffusion method using standard levofloxacin reference drug. In the case of pyrazole-embedded pyrimidines, **773** displayed best activity against MRSA with low MIC of 521 μM in comparison of standard levofloxacin (MIC = 346 μM). Among the pyrazoline derivatives, unsubstituted **774** showed better activity towards MRSA (Gram-positive) than **775** with an acetyl functionality. Potent inhibitor **773** was evaluated *in vivo* and remarkably reduced the infection (*p* < 0.0001) while treating MRSA induced keratitis in case of rats. Docking studies revealed that, pyrimidine analogue **773** with the TMP binding site of *Staphylococcus aureus* DHFR enzyme exhibited a unique binding profile with various hydrophobic and hydrophilic interactions with the amino acid residues (Binding energy = −13.6169 kcal/mol). On the other hand, pyrazoline analogue **774** displayed good interactions with a binding energy score of −12.0047 kcal/mol, demonstrating the unsubstituted **774** was active than acetyl derivative **775**. *N*-(trifluoromethyl)phenyl bearing pyrazole derivatives were synthesized and investigated for their antibacterial effect against various strains of Gram-negative and positive bacteria (Alam et al.) [201]. It was found that, these compounds shown better activities against various Gram-positive bacterial strains but not against Gram-negative strains. Among the several prepared compounds, **776** and **777** exhibited excellent inhibitory effects. Especially, the phenoxy derivative **777** better inhibited the growth of staphylococcal strains with the MIC values of 1.56–3.12 μg mL^−1^. Growth of *E. faecalis* and *E. faecium* strains were inhibited with the MIC values of 1.56 μg mL^−1^ and 3.12 μg mL^−1^. B. subtilis was also effectively inhibited with the MIC values of 1.56 μg mL^−1^. Potent compounds **776** and **777** showed least toxicity to human embryonic kidney cells (HED293) with IC_50_ values of 8 and 23.5 μg mL^−1^. Time kill assay (TKA) showed that **777** eradicated 99 % bacteria by 6 h whereas, the vancomycin positive control took 10 h. Additionally, **777** showed two log reduction against persister cells compared to gentamicin which reduced by one log. Phenoxyphenyl derivative (**777**) inhibited the biofilm growth of *E. faecalis* efficiently. Synthesis and antibacterial evaluation of coumarin linked pyrazoles have been described by (Aziem et al.) [202]. Variety of Gram-positive and negative bacterial strains were evaluated by using standard drug *Penicillin G* (Gram-positive) and Ciprofloxacin (Gram-negative). Pyrazole derivative **778** was the most potent against Bacillus pumilis with the MIC of 7.69 μg mL^−1^. In addition, **778** showed excellent activity towards Streptococcus faecalis with MIC of 15.36 μg mL^−1^. Most of the tested compounds shown moderate MIC values (4.73–45.46 μmol/mol) against *E. Coli.* A novel series of highly substituted pyrazole and pyrazoline derivatives were synthesized and tested for their *in vitro* antibacterial potency against various pathogenic bacterial strains using standard reference drug (Hassan et al.) [203]. Compound **779** displayed higher inhibitory effect against *B.subtilis* (IZ = 20 mm) in comparison to the reference drug Vancomycin (IZ = 21 mm). In case of Gram negative strain (*E.coli*), **780** and **781** showed excellent inhibitory effect (IZ = 20 mm) in comparison to the reference drug Negram (IZ = 16 mm). In addition, few of the synthesized candidates were also tested for their potential anti-fungal activity. Drug likeliness calculations revealed that, compounds **780** and **781** possessed a maximum of drug-like score (DLS) of 0.75 and 0.83. A new collection of pyrazoles, pyrazoline and indazole drug-like compounds were described by Baumann et al. [204] *via* continuous flow method and their anti-bacterial activity was studied against 18 bacterial strains including Gram-positive and negative species. Most of the prepared compounds were active towards Gram-positive strains but not very active against Gram-negative species. Among all the pyrazole derivatives, **782** possessing pyrazoline ring and a fused imide unit was found to be very active against most common genus staphylococcus, with low MIC values (4 μg mL^−1^). To investigate the specific mechanism of action of **782**, time kill experiments were carried out on each strain of S. aureus. Furthermore, pharmacokinetics properties and drug-likeness calculations by using Swiss ADME tools revealed that compound **782** could act an efficient inhibitor of CYP3A4 isoform. Pyrazole derivatives clubbed benzodiazepines were prepared *via* a one-pot method and their effect was studied against various bacterial strains such as *P. aeruginosa*, *E. coli*, *S. aureus* and *S. pyogenes via* serial dilution method (Desai et al.) [205]. Compound **783** in displayed excellent effect towards *E. coli* and *P. aeruginosa* with MIC of 12.5 μg mL^−1^ whereas, **784** exhibited potential activity against S. aureus. As demonstrated by the molecular docking studies, the active sites of DNA gyrase target were effectively bound by these compounds with excellent docking scores (−8.298 to −6.738) and binding energies of −55.628 kcal/mol and −48.342 kcal/mol. Key interacting amino acid residues and their influencing type of thermodynamic interactions were identified by using *in silico* per-residue interaction analysis. SAR studies demonstrated that the introduction of electron-withdrawing substituents on the aryl unit enhanced the antimicrobial activity. In addition, some of these compounds were displayed excellent anti-fungal activities. A novel series of pyrazole derivatives tethering triazines (azo pyrazoles) were prepared and evaluated for their antibacterial effects (Al-Azmi et al.) [206]. All the synthesized compounds displayed moderate inhibition against *Bacillus subtilis*. Triazine bearing pyrazole derivative **785**–**787** inhibited *Pseudomonas aeruginosa* but were not active against *E. coli* (Fig. 8). On the other hand, towards Gram-positive *B. subtilis* moderate activity was exerted but no significant activity against *S. aureus*.

By treating aromatic aldehydes and 5-aminopyrazoles a series of Schiff bases were prepared and evaluated for their *in vitro* antibacterial effects towards several Gram-positive and negative bacterial strains such as *Staphylococcus aureus*, *Staphylococcus epidermis*, *Enterococcus faecalis*, *Acinetobacter baumannii*, *Enterobacter cloaca*, *Escherichia coli* (Hassan et al.) [207]. Compounds **788**, **789**, **790**, **791** and **792** were shown excellent activities with minimal inhibitory concentration in the range of 7.81 μg mL^−1^ to 31.25 μg mL^−1^ (Fig. 9). Further studies revealed that, all the selected compounds had good drug score by fulfilling Lipinski's rule. *In vitro* enzyme inhibitory assay for **788** was performed towards S. aureus DNA gyrase, DHFR and topoisomerase IV and revealed that, excellent inhibitory activity was exerted for DNA gyrase and DHFR in comparison to the standard reference Methotrexate. The interactions and binding modes of **788** with DNA gyrase and DHFR was further analysed by molecular docking studies. Arene-arene interaction, arene-cation interactions and two hydrogen bonds were established between **788** and amino acid residues of *S. aureus* DNA gyrase. In case of DHFR, two H-bond interactions were formed between N1 and N2 of pyrazole skeleton with Ser59. Novel series of dihydrotriazines possessing 1,3-diaryl pyrazole derivatives were synthesized and investigated towards various Gram negative and positive bacterial strains. Most of the compounds in this report were active towards the bacterial species including multidrug-resistant isolates (Piao and Zhang et al.) [208]. Compounds **793** and **794** exhibited potential activity against *S. aureus* and *E. coli* with MIC of 1 or 2 μg mL^−1^ (Fig. 9). Antibacterial activity of **794** through DHFR inhibition was implied by *in vitro* enzyme study. The interaction of **793** and **794** with the active site of S. aureus DHFR enzyme was explained by the preliminary docking studies. Accordingly, a π-alkyl interaction between the aryl ring and Phe92 was established. Alkyl bond between pyrazole N atom and Leu20, H-bonding interaction between NO_2_ group and Gly94 and Gln95 and H-bonding between dihydrotriazin ring aand Gln19 amino acid residues were also established between the active compounds and target enzyme. The cytotoxicity of active compounds was observed as IC_50_ > 100 μmol/L against L02 cells proving the observed antibacterial activity was not due to their toxicity. A series of *N*-pyrazolyl benzamides were prepared and studied for their antibacterial activity against various strains (Bikshapathi et al.) [209]. Compounds **795** and **796** were shown higher activity against *Bacillus subtilis*, *Klebsiella pneumoniae* and *Staphylococcus aureus* with minimum inhibitory concentration of 3.12 μg mL^−1^. Compound **796** was found to have excellent antitubercular effect with MIC of 6.25 μg mL^−1^towards H37Rv mycobacterial strain. Moreover, **796** exhibited MIC of 3.12 μg mL^−1^ towards VRSA and MRSA and MIC of 6.25 μg mL^−1^ towards E. faecium and MTB. In addition, these compounds were displayed significant anti-inflammatory activity. 70 new coumarin-pyrazole carboxamide hybrid structures under six series were prepared and evaluated for their *in vitro* antibacterial effect using agar dilution method [210]. Four important bacterial strains such as *Staphylococcus aureus*, *Escherichia coli*, *Listeria monocytogenes* and *Salmonella*. It was found that, **797** shown significant inhibition against E. coli (MIC = 0.25 mg/L). **798** exhibited remarkable activity against Salmonella (MIC = 0.05 mg/L) in comparison with ciprofloxacin. Moreover, **797**, **798** and **799** were shown excellent enzyme inhibition towards *Topoisomerase II* and *Topoisomerase IV* with IC_50_ in the range of 9.4–25 mg/L. Docking studies revealed that, potential compounds effectively bind to the amino acid residues of Topo II enzyme receptor suggesting that, these active candidates have a perfect shape complementary to the binding pockets of the enzyme. Both **797** and **798** shown similar docking results. A new set of 30 pyrazole derivatives were prepared using readily available precursors and investigated for their antibacterial activity against four bacterial species such as methicillin-resistant *S. aureus*, *Staphylococcus aureus*, *Bacillus subtilis* and *Acinetobacter baumannii* (Alam et al.) [211] Compound **800** with bis-fluoro substitution exhibited excellent activity towards *S. aureus*, *MRSA* and *Acinetobacter baumannii* with an MIC of 12.5 μg mL^−1^. Against *B. subtilis* activity was exerted with MIC 3.12 μg mL^−1^. Bis-chloro compound **801** displayed higher activity against all the tested Gram-positive strains with MIC of 0.78 μg mL^−1^. Out of all compounds, **802** with 4-CF_3_ unit showed highest activity with MIC of 0.78 μg mL^−1^against Gram-positive species (Fig. 9). Selected compounds were tested for the cytotoxicity towards the growth inhibition of human embryonic kidney (HEK-293) cells *via* resazurin assay (25 and 50 μg mL^−1^). It was found that, none of these active compounds displayed growth inhibition even after incubating for 24 h. More than 80 % cell viability was observed.

### 4.4 Anti-viral activity of pyrazoles

A sequence of pyrano[2,3-*c*]pyrazoles were tested for their cytotoxicity and antiviral effects towards human coronavirus 229E Vero-E6 cell lines (HCoV-229E) (Saleh et al.) [212] Compound **806** exhibited excellent inhibitory capacity towards human Covid virus 229E with high selectivity index (SI = 12.6). It was also found that, **803**, **804**, **805** and **806** inhibited SARS-CoV2 protease enzymes with IC_50_ values of 44.78, 359.5, 70.3, and 27.8 μg/mL compared to tipranavir (IC_50_ = 13.32 μg/mL) (Fig. 10). Best interaction and binding score were observed for the docking of active compound **806** with SARS-CoV2 main protease enzyme. The binding pattern include four H-bonds and one hydrophobic interaction were established between the active compound and various amino acid residues of enzyme target. Importantly, the active compounds shown least cytotoxicity against Vero-E6 cell lines. Diversely substituted pyrazole-biphenyl derivatives were prepared in good yields *via* a multi-step procedure and investigated for inhibitory efficiency of entry level SARS-CoV2 infection (Dangate et al.) [213]. These molecules inhibited the association of SARS-CoV2 protein spike (SC2SP) and human angiotensin-converting enzyme-2 (hACE2). Based on the molecular docking studies, binding energies, ki values and ligand efficiency **807** and **808** were identified as potential compounds with the binding energies ranging from −7.3 kcal/mol to −9.7 kcal/mol. Through MTT assay and live-dead assay, potential compounds were tested for their cytotoxicity against human embryonic kidney cells. It was observed that even at the concentration levels of 75 μg mL^−1^ these compounds were non-toxic. Pyranopyrazoles bearing diaryl groups at N-1 and C-4 were prepared and tested for their activity against Coronaviruses and related inflammations (Abulkhair et al.) [214]. These compounds were investigated *in vitro* against SARS-CoV affected Vero cells. Compounds **809**, **810** and **811** wer shown higher inhibition towards the viral main protease with the IC_50_ values of 2.01, 1.83 and 4.60 μM compared to the standard drug lopinavir. Docking studies revealed that, selected compounds interacted non-covalently with SARS-CoV main protease. *In vivo* pharmacokinetic profiles of **810** revealed its ADME properties and stability. The interaction between the active pyranopyrazoles with SARS-CoV-2M^pro^ target protein was studied by docking studies. Compound **810** oriented in the active pocket of SARS-CoV-2M^pro^ with a docking score of −16.459 kcal/mol and binding energy of −6.8 kcal/mol. Excellent binding affinity was exerted through various bonding and non-bonding interactions of amino-acid residues. On the other hand, cytotoxicity studies revealed that, **811** was highly cytotoxic to Vero E6 cell lines (IC_50_ = 18.1 μM) whereas, the **809** and **810** were moderately cytotoxic with IC_50_ > 60 μM. Additionally, *in vivo* anti-inflammatory studies were also performed. Anti-JEV activity (*in vitro* and *in vivo*) was explored with a new set of di-nitroaryl possessing pyrazole derivatives (Kumar and Desai et al.) [215]. Neuro2a cells were treated with JEV at 0.01 MOI and post-treated with the hit compounds **812** and **813**. Dose response studies and *in vitro* experiments revealed that these compounds were displaying over 70 % and 90 % inhibition. On the other hand, *in vivo* studies of **812** and **813** reasonably reduced the viral RNA by 41 % and 70 % in spleen tissue. In case of brain tissue reduction was 33 % and 43 %. Quantification of viral mRNA from cells was done by qRT-PCR. Hit compounds (**812** or **813**) did not show any toxicity when administered intraperitoneally at a dose of 100/kg/day. A series of pyrazole carbamide derivatives containing sulfonate moiety were synthesized and tested *in vivo* for their activity against TMV by using half leaf blight spot method at 500 mg/L (Lei et al.) [216]. Target compounds exhibited significant protective effects against TMV. Compound **814** displayed curative activity similar to standard ningnanmycin (55.3 %). In terms of protective effects, **815** and **816** shown noticeable activities (50.4 % and 50.2 %). Compared to the standard Chitosan oligosaccharides, few compounds exhibited reasonable inactivation activities. Additionally, these compounds were also screened for *in vitro* antifungal activity against plant pathogens. A set of fused pyrazolo[5′,1’:2,3]imidazo[1,5-*c*]quinazolin-6(5*H*)-ones were accessed through a one-pot multicomponent reaction and their affinity towards the COVID-19 main protease (Rahmati et al.) [217]. Detailed *in silico* investigations revealed that various parameters such as, resonance effects, electronegativity, hydrophobic interaction, hydrogen bonding had influencing effects on the inhibition and binding ability of the compounds. Among the obtained products, **817**, **818** and **819** were shown excellent binding energies (−8.77 kcal/mol, −8.73 kcal/mol and −8.63 kcal/mol) and proper affinity towards COVID-19 main protease compared to the standard drugs. A series of 2-aryl-1*H*-pyrazole-S-DABOs as non-nucleoside reverse transcriptase inhibitors (NNRTIs) were developed with C6-structural variations and their anti-DENV (Dengue virus) and anti-HIV effects were studied (He and Zheng et al.) [218]. Most of the compounds in this series were displaying low cytotoxicity (EC_50_ = 0.058–0.0966 μM). Compounds **820** and **821** were efficiently inhibited the DENV(I-IV) replication at the cellular level (Fig. 10). Especially, EC_50_ of **820** and **821** against DENV-II were observed in the range of 13.2 ± 5.03 μM and 9.23 ± 4.71 μM, which was higher than the standard ribavirin (EC_50_ = 40.78 ± 1.02 μM). Structural modification revealed that, cyclohexyl group at C6 was mandatory for the anti-DENV activity. In addition, these compounds were also displayed potential anti-HIV activities with low cytotoxicity with EC_50_ values in the range of 0.051–0.141 μM.

1,3,4-oxadiazole tethered 1-*tert*-butyl-5-amino-4-pyrazoles derivatives were prepared and tested for their protective activity against TMV (Wu et al.) [219]. Among the compounds tested, **822**, **823**, **824** and **825** were displayed excellent activities with EC_50_ values of 165.8, 163.2, 159.7, 193.1 mg/L compared to ningnanmycin reference standard (EC_50_ = 271.3 mg/L) (Fig. 11). Compound **823** significantly increased the defense enzyme activities and chlorophyl contents. *In vivo* studies revealed that spread of TMV in inoculated tobacco was efficiently inhibited by **823**. TEM based morphological studies of **823** revealed that it can lead to a distinct breakage of the rod-shaped TMV. A new series of non-DKA derivatives bearing pyrrolyl-pyrazole carboxylic acids were synthesized and tested *in vitro* towards recombinant HIV-1 RT associated inhibition of RNase H (Costi et al.) [220]. Most of the compounds in this series were shown higher selectivity for RNase H. Particularly, oxy-phenylpyrrolyl-pyrazole sets displayed superior inhibitory effects. **826** was the promising compound inhibited RNase H with IC_50_ = 0.27 μM. Mode of binding was rationalized *via* molecular docking studies and site-directed mutagenesis experiments. *In vitro* anti-integrase (IN) activity was also assayed using HTRF-based solution assay method in presence of WT IN and LEDGF. It was observed that **827** shown 5-fold selectivity to IN with IC_50_ value of 75 μM. Docking studies of active compounds within the site of RNase H indicated that, H-bonding, cation-π interaction and chelations were established during the specific interactions. Few compounds of this series were not cytotoxic up to 200 μM concentration. Pyrazole derivatives clubbed with adamantane were tested for their *in vitro* antiviral activity towards Food and Mouth Disease (FMD) viruses using baby hamster kidney (BHK-21 cells) (El-Din et al.) [221]. Compounds **828**, **829** and **830** displayed promising antiviral activities and suppressed FMDV replication with a therapeutic index of 30 (Fig. 11). Cytotoxicity concentration (CC_50_) of these selected derivatives on BHK-21 cells were in the range of 500–3000 μg/mL. *In vivo* studies on baby mice revealed that **828** and **829** led to 100 % survival at 50 μg/mL, whereas **830** gave at 50 μg/mL compared to amantadine standard. Potential compounds were docked into the active site of 3C protease enzyme target efficiently with the binding energies of −15.41 to −20.0 kcal/mol compared to that of standard drugs Amantadine (−6.15 kcal/mol) and Ribavirin (−10.77 kcal/mol). High binding energy values of these compounds demonstrate their 3C protease inhibition capacity. *In vitro* anti-HIV activity of pyrazolo[4,3-*c*]pyridin-4-one derivatives was investigated towards HIV-1_VB59_ (R5, subtype C) and HIV-1_UG070_ (X4, subtype D) in TZM-b1 cell line (Singh et al.) [222]. Compound **831** exhibited excellent activity against both the viral strains with IC_50_ of 3.67 μM and 2.79 μM, and high therapeutic indices (TI) of 185 and 243. In addition, **831** was also potent towards nevirapine drug resistant isolates such as, HIV-1_N119_ (X4/R5, subtype B) and HIV-1_NARI-DR_ (R5, subtype C) with IC_50_ of 3.24 ± 0.9 μM and 2.53 ± *μ*M. Binding of active compound **831** within the pocket of HIV-1 reverse transcriptase was analysed by docking studies. Protein ligand interactions showed that, important hydrophobic interactions were found between the pyridinone of **831** and amino acid residues (Y181 and Y188). Calculated binding energy for **831** was −10.5 kcal/mol. A novel class of phenyl-2*H*-pyrazolo[3,4-*d*]thiazol-5(6*H*)-imines were prepared and investigated for anti-HIV-1 activity *via* enzymatic and cell based assay methods (Bhusare et al.) [223]. At 100 μg mL^−1^ the inhibition activity for HIV-1 RT was observed in the range of 60–94 %. Assay methods and priliminary docking studies reavealed that **832** and **833** exhibited excellent inhibitory effects (90.57 % and 89.80 %). Enzyme based assay shown that these candidates were highly effective towards reverse transcriptase (RT) enzyme. It was also demonstrated that, HIV-1 IIIB replication was effectively inhibited by **832** and **833** with the EC_50_ values of 0.74 and 1.08 μg mL^−1^ respectively. In addition, replication of HIV-1 ADA5 was inhibited with EC_50_ values of 1.08 and 0.34 μg mL^−1^. Upon docking the selected compounds **832** and **833** to the active site of HIV-1 reverse transcriptase (target protein) excellent binding energies of −7.849 kcal/mol and −7.594 kcal/mol was observed. Further studies confirmed that, π-π interactions between phenyl-thiazolyl unit and aromatic side chains of Trp229 and lys103 amino acid residues occurred. New class of 1-(phenylsulfonyl)-1*H*-pyrazol-4-yl-methylaniline compounds were accessed and their antiviral effects were described towards a large array of RNA (ssRNA^+^ and ssRNA^−^) and DNA viruses using cell-based assay method (Desideri et al.) [224]. Variously substituted compounds were tested and found that, compound **834** was found to be potent YFV inhibitor (EC_50_ = 3.6–11.5 μM. Compound **835** series displayed good inhibition towards RSV replication (EC_50_ = 8.5–24.0 μM). In addition, compound **836** was effectively inhibited BVDV replication (EC_50_ = 5.6 μM). Most of the studied derivatives, including the potential compound (**835)** displayed low cytotoxicity against BHK-21 cell lines up to 100 μM concentration. However, **834** and **836** showed significant toxicity towards Vero cells (CC50 = 10.0 and 3.0 μM). Pyrazole-hydrazone hybrid derivatives bearing isoxazole unit were prepared and their anti-viral activities was investigated against tobacco mosaic virus (TMV) using a half-leaf method (Yang et al.) [225]. *In vivo* biological evaluation indicated that, compounds **837**, **838** and **839** displayed significant curative effect towards TMV with 55.6 %–56.2 % inhibition rates at 500 μg/mL which was comparable to Ningnamycin (54.6 %) (Fig. 11). These compounds were shown the EC_50_ values in the range of 240.8–267.4 μg/mL. In addition, some of the prepared compounds were displayed moderate protection activity towards TMV with good inhibition rates (64.2 %–66.2 %). It was also found that, some of these compounds were possessing remarkable inactivation activities towards TMV with 91.4%–92.6 % inhibition rates. A new group of compounds bearing 4-pyridinyl, 1*H*-triazol-1-yl and 1*H*-pyrazol-1-yl were prepared and investigated for *in vitro* anti-rhinoviral activity towards RV-B14 using Pleconaril as a reference binder (Vanelle et al.) [226]. Compared to other analogues, pyrazole derivatives **840** and **841** were found to be more potent against RV-B14 with EC_50_ values in the range of 0.2 μM. It was also realized that, chirality influenced the pharmacological activity of this class of compounds. (*R*)-**840** enantiomer displayed similar activity as the racemic compound but (*S*)-**840** exhibited 5.7-fold potency. Similarly, (*S*)-**841** shown three-fold more activity compared to its racemic counterpart. Binding modes of **840** and **841** with the active pockets of viral protein (VP1) differed between the (*R*) and (*S*) enantiomers. Compound cytotoxicity studies uninfected-compound treated cells explained that, CF_3_-substituted analogues were shown significant cytotoxicity (CC50 = 14–21 μM).

## 5 Conclusion

Pharmaceutical and material applications of pyrazole skeletons have enabled extensive progress in their synthesis and biological studies. Owing to this excellence, numerous synthetic approaches were reported based on transition metal catalysis, Lewis acid promoted reactions, cascade annulations, dipolar cycloadditions, photoredox catalysis. Last decade has evidenced exponential progress in the atom-economic development of multi-functionalized pyrazoles in one-pot process using novel reactants. In this detailed review, we have particularly included the recent synthetic insights of various pyrazole-based compounds from alkynes, α,β-unsaturated carbonyl compounds, nitrilimines, diazo compounds, diazonium salts and 1,3-dicarbonyl compounds. Utility of these substrates resulted in regioselective and/or stereoselective pyrazoles in a mechanistically interesting pathway. In addition, we have also included the reports for pyrazolines, pyrazolones and fused pyrazoles. Important biological studies such as, anticancer, anti-inflammatory, antibacterial and antiviral activities of various pyrazole derivatives reported in recent years also included to showcase the potential of these ubiquitous cores. In future perspective, owing to the amplified utility of pyrazole derivatives in medicinal chemistry (NSAIDs), material chemistry (metal organic frameworks), organometallic chemistry, and catalysis these cores are important choice of N-heterocyclic templates for the chemists. We believe that, the innovative approaches discussed herein, would enable the synthetic community to develop more insights and contribute to the synthesis of broad-spectrum N -heterocycles.

## Funding Statement

Ziad Moussa is grateful to the 10.13039/501100006013United Arab Emirates University (UAEU) and to the Research Office for supporting the research developed in his laboratory and reported herein (SUREPLUS Grant code G00004305, UPAR Grant code G00004605).

## Data and code Availability statement

No data was used for the research described in the article.

## CRediT authorship contribution statement

**Ziad Moussa:** Writing – review & editing, Writing – original draft, Supervision, Software, Funding acquisition, Data curation, Conceptualization. **Mani Ramanathan:** Writing – review & editing, Writing – original draft, Data curation. **Shaikha Mohammad Alharmoozi:** Writing – review & editing, Methodology, Data curation. **Shahad Ali Saeed Alkaabi:** Writing – review & editing, Methodology, Data curation. **Salamah Hamdan Mohammed Al Aryani:** Writing – review & editing, Investigation, Data curation. **Saleh A. Ahmed:** Writing – review & editing, Investigation, Data curation. **Harbi Tomah Al-Masri:** Writing – review & editing.

## Declaration of competing interest

The authors declare that they have no known competing financial interests or personal relationships that could have appeared to influence the work reported in this paper.
